# Review of Plasma-Synthesized/Modified Polymer and Metal Nanoparticles for Biomedical Applications Using Cold Atmospheric Pressure Plasma

**DOI:** 10.3390/polym17212856

**Published:** 2025-10-26

**Authors:** Eun Young Jung, Bhum Jae Shin, Habeeb Olaitan Suleiman, Heung-Sik Tae, Choon-Sang Park

**Affiliations:** 1 The Institute of Electronic Technology, College of IT Engineering, Kyungpook National University, Daegu 41566, Republic of Korea; eyjung@knu.ac.kr; 2 Department of Electronics Engineering, Sejong University, Seoul 05006, Republic of Korea; hahusbj@sejong.ac.kr; 3 School of Electronic and Electrical Engineering, College of IT Engineering, Kyungpook National University, Daegu 41566, Republic of Korea; suleiman.habeeb16@knu.ac.kr; 4 Department of Electrical Engineering, Milligan University, Johnson City, TN 37682, USA

**Keywords:** cold atmospheric pressure (CAP), atmospheric pressure plasma (APP), dielectric barrier discharge (DBD), plasma jet, polymer film, metal nanoparticles (NPs), plasma synthesis, plasma surface treatment

## Abstract

This review presents recent advancements in cold atmospheric pressure (AP) plasma (CAP) processes for the synthesis and surface treatment of polymer films and metal nanoparticles (NPs) in biomedical applications. We discuss the properties and applications of atmospheric pressure plasma (APP) processes, including dielectric barrier discharge (DBD) and plasma jet methods, highlighting their effectiveness in controlling surface characteristics such as wettability and functionalization.

## 1. Introduction

In general, plasma is a partially ionized state of matter composed of ions, electrons, and neutral particles, and it is classified into two main types: thermal plasma and non-thermal cold plasma (NTCP) [1,2,3,4]. Thermal plasma exists in thermal equilibrium, where the temperature of electrons and ions is comparable, and is primarily employed in high-temperature industrial applications, making it unsuitable for biomedical applications [5]. In contrast, NTCP operates under non-equilibrium conditions, where electron temperatures are significantly higher than ion temperatures [1,2,3,4]. NTCP enables the synthesis and surface treatment of polymers and metal nanoparticles (NPs) at room temperature (RT) through reactions involving energetic electrons during plasma discharge, thereby enhancing properties such as wettability, biocompatibility, antimicrobial resistance, and drug delivery, as well as supporting applications in sterilization, dentistry, dermatology, and implant treatments [6,7,8,9,10,11,12,13]. Atmospheric pressure (AP) –generated NTCP, commonly referred to as cold AP plasma (CAP), produces reactive species including reactive oxygen species (ROS), reactive nitrogen (N_2_) species (RNS), UV photons, and charged particles, which are highly advantageous for biomedical applications, such as wound healing, decontamination, implant surface treatment, dentistry, sterilization in dermatology, and cancer therapy [14,15,16,17,18]. CAP can be generated under vacuum, low–pressure, or AP plasma (APP). As a result, APP has received significant attention in recent years for plasma synthesis and surface modification of polymer films and metal NPs across various industrial applications. Current research has investigated APP–based plasma synthesis and surface modification of polymers and metal NPs using APP devices such as dielectric barrier discharge (DBD) and plasma jets [15,19,20,21,22,23,24]. Notably, CAP treatment is widely applied using plasma jets under ambient air at room temperature for clinical treatments in plasma biomedicine, including wound healing, tissue treatment, and cancer therapy, as it enables selective targeting of localized areas while minimizing damage to surrounding healthy cells. In direct CAP treatment, reactive oxygen and nitrogen species (RONS), which are radical species, interact directly with biological cells, thereby promoting cell growth, enhancing wound healing, and inducing selective apoptosis in cancer cells [25,26,27,28,29,30]. For desirable clinical treatments, the CAP intensity acting on biological cells needs to be appropriately controlled. Here, CAP intensity is expressed in terms of plasma dosage, which corresponds to the density of RONS formed in the plasma. This plasma dosage depends on various parameters such as plasma source, voltage, current, gas type, gas flow rate, treatment time, and distance between electrodes [31,32,33]. Consequently, CAP processes for plasma synthesis and surface modification are increasingly employed in biomedical applications. This review examines recent studies on polymer films and metal NPs, focusing on CAP–based synthesis and surface modification methods. The objective is to provide a comprehensive reference on recent plasma processes for the synthesis and surface treatment of polymer films and metal NPs in biomedical applications. This review is organized into three sections. Section 2.1 addresses plasma polymer synthesis, Section 2.2 discusses plasma surface modification of polymers, and Section 3 summarizes plasma synthesis and surface treatment of metal NPs. All figures are reproduced with copyright permission from the respective publishers.

## 2. Plasma Process

### 2.1. Plasma Synthesis of Polymer Films for Biomedical Applications

**Figure 1:**
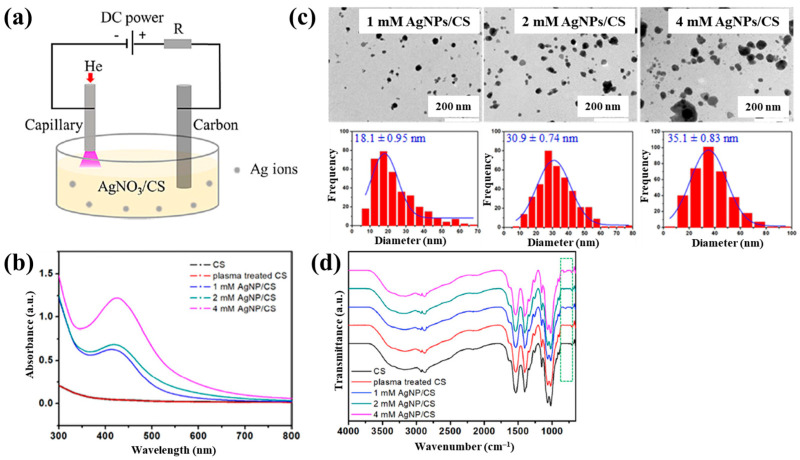
(**a**) Experimental setup for synthesis of AgNP/chitosan (AgNPs/CS) composites using AP microplasma (APM) by Sun et al. (**b**) UV–vis spectra of CS and plasma–treated CS. (**c**) Transmission electron microscopy (TEM) images and size distribution of AgN–/CS composites prepared with 1, 2, and 4 mM AgNO_3_/CS mixtures. (**d**) FT–IR spectra of CS, plasma–treated CS, and AgNP/CS composites synthesized with 1, 2, and 4 mM AgNO_3_/CS mixtures. Reproduced with copyright permission from Ref. [34].

Sun et al. [34] investigated the synthesis of silver NP/chitosan (AgNP/CS) composites for antibacterial applications. Figure 1a shows the experimental setup for the synthesis of AgNP/CS composites using AP microplasma (APM), where a carbon rod served as the anode electrode and was immersed in the AgNO_3_/CS solution, while a stainless steel capillary with an inner diameter of 250 μm acted as the cathode electrode, positioned 2 mm above the solution surface. Helium (He) gas at a flow rate of 25 sccm was injected through the capillary to initiate plasma discharge. A voltage of 4 kV was applied to the cathode electrode, enabling AgNP/CS composite synthesis in the liquid plasma environment. Upon plasma ignition in the AgNO_3_/CS solution, the voltage dropped to 2.8 kV, and a discharge current of 5 mA was maintained throughout plasma synthesis for 10 min. The optical properties of plasma-treated solutions were analyzed by UV–Vis spectroscopy. As shown in Figure 1b, no absorption peak was detected in either untreated or plasma-treated CS solutions, despite slight color changes. In contrast, plasma-treated AgNO_3_/CS solutions exhibited more pronounced color changes, which correlated with increasing AgNO_3_ concentration. Transmission electron microscopy (TEM) images (Figure 1c) revealed that the synthesized AgNPs were well dispersed and that particle size increased with higher AgNO_3_ concentrations. The chemical structures of all AgNO_3_/CS samples were characterized by Fourier-transform infrared (FT–IR) spectroscopy. As shown in Figure 1d, the broad bands at 3200–3500 cm^−1^ originated from –OH and –NH_2_ stretching vibrations in the plasma-treated AgNO_3_/CS samples. The peak at 1647 cm^−1^ corresponded to C–O stretching of the O–C–NHR amide functional group. During plasma-assisted composite formation, ROS, such as –OH and C–O, were incorporated into the composite. Additional peaks at 2927, 2884, 1411, 1380, 1321, 1260, and 1078 cm^−1^ were assigned to C–H and N–H vibrations, indicating interactions between AgNPs and CS [34].

**Figure 2:**
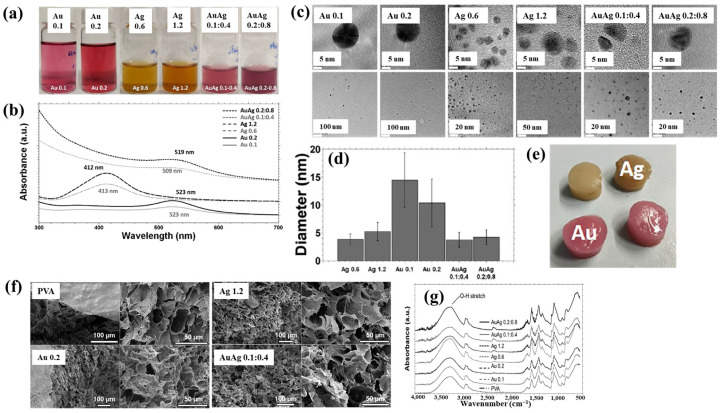
(**a**) Photographic images and (**b**) UV–vis absorption spectra of plasma–treated polyvinyl alcohol (PVA) solutions containing AuNPs, AgNPs, and AuAgNPs. (**c**) TEM images and (**d**) particle size distribution of synthesized metal NPs. (**e**) Photographic images, (**f**) scanning electron microscopy images (SEM), and (**g**) FT–IR spectra of metal NP/PVA hydrogels prepared under different metal NPs conditions. Reproduced with copyright permission from Ref. [35].

Nolan et al. [35] investigated AuAg NP/polyvinyl alcohol (PVA) hydrogels synthesized via non-thermal APP for antibacterial applications [35]. The hydrogels were synthesized using a similar plasma system (Figure 1a) to that proposed by Sun et al. [34]. For hydrogel synthesis, He gas at a flow rate of 25 sccm was injected through the capillary, which was connected to a power supply via a platinum (Pt) wire acting as the anode in the PVA/salt solution. Plasma discharge was achieved by applying a voltage of 2 kV and a current of 2 mA. As shown in Figure 2a, plasma treatment induced distinct color changes in gold(III) chloroauric acid trihydrate (H[AuCl_4_]·3H_2_O)/PVA and AgNO_3_/PVA solutions. The Ag-treated solution exhibited a yellow color due to colloidal Ag, whereas the plasma-treated H[AuCl_4_]·3H_2_O solution shifted to red or purple, indicating AuNP formation. The presence of Au and Ag NPs in plasma-treated solutions was confirmed by UV–vis absorption spectra (Figure 2b). Specifically, plasma-treated H[AuCl_4_]·3H_2_O/PVA solutions showed an absorption peak at 523 nm, characteristic of the surface plasmon resonance (SPR) of AuNPs. Similarly, plasma-treated AgNO_3_/PVA solutions displayed SPR peaks at 413 and 412 nm for Ag 0.6 and Ag 1.2 samples, respectively, confirming AgNP formation. TEM analysis (Figure 2c) revealed that all synthesized metal NPs were well dispersed, spherical, and free from agglomeration. The particle size distributions (Figure 2d) showed the distribution of Ag0.6 and Ag1.2 samples to be 4–5 nm, with Au0.1 and Au0.2 NPs ranging from 10 to 15 nm, and bimetallic AuAg0.1:0.4 and AuAg0.2:0.8 NPs averaging ~4 nm. To evaluate antibacterial performance, NP/PVA hydrogels were fabricated. For AgNO_3_/PVA hydrogels, AgNO_3_/PVA solutions were poured into a polytetrafluoroethylene (PTFE) mold, frozen at −20 °C for 14 h, and thawed at 18 °C for 10 h over four freeze–thaw cycles. Figure 2e shows the photographic images of the resulting NP/PVA hydrogels, each with dimensions of 9 × 14 mm. SEM analysis (Figure 2f) revealed that untreated and plasma-treated PVA hydrogels displayed similar morphologies. FT–IR spectra (Figure 2g) confirmed a broad peak at 3300 cm^−1^ in plasma-treated PVA hydrogels, corresponding to O–H bonds attributed to hydrogen peroxide (H_2_O_2_) and ROS radicals (O, H, and OH) generated during plasma treatment. These results demonstrate that AuAg NPs were successfully incorporated into PVA hydrogels by APP, producing composite materials with potential for biomedical applications [35].

**Figure 3:**
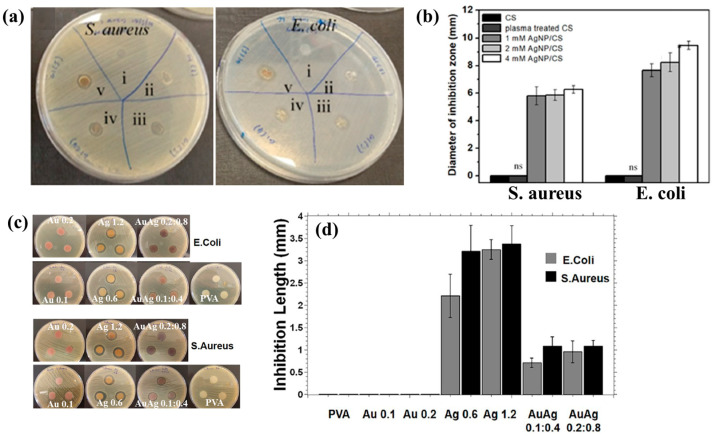
(**a**) Antibacterial effects against *S. aureus* and *E. coli* under five conditions: (i) untreated CS, (ii) plasma–treated CS, (iii) 1 mM AgNPs/CS, (iv) 2 mM AgNPs/CS, and (v) 4 mM AgNPs/CS. (**b**) Comparisons of average inhibition zone diameters against *S. aureus* and *E. coli* under five conditions. (**c**) Antibacterial activity and (**d**) inhibition zone lengths of AgAu NP/PVA hydrogels against *E. coli* and *S. aureus*. Reproduced with copyright permission from Refs. [34,35].

Figure 3a,b illustrate the antibacterial properties and average inhibition zone diameters of two bacterial strains (*Escherichia coli* (*E. coli*) and *Staphylococcus aureus* (*S. aureus*)), tested with different composite samples: (i) untreated CS, (ii) plasma−treated CS, (iii) 1 mM AgNPs/CS, (iv) 2 mM AgNPs/CS, and (v) 4 mM AgNPs/CS [34]. No antibacterial activity was observed in the untreated or plasma−treated CS samples, whereas all AgNP/CS composites exhibited clear inhibition zones against both bacterial strains. Notably, in the case of *E. coli*, the inhibition zone diameter increased with higher Ag content. The strong antimicrobial properties of AgNPs are attributed to ROS generated during plasma treatment. When these ROS species interact with bacteria in plasma−treated composite films, they disrupt cellular functions and ultimately induce bacterial death [34].

Additionally, Figure 3c,d present the antibacterial activity and inhibition zone lengths of various AuAg metal NP/PVA hydrogels against *E. coli* and *S. aureus*. The inhibition zones are most prominent around the AgNP/PVA composite sample, indicating that this sample exhibits superior antibacterial activity. The strong antimicrobial properties of AgNPs are attributed to their enhanced surface electronic property and the generation of ROS induced by plasma treatment. When these ROS interact with bacteria within the plasma-treated composite hydrogels, they disrupt cellular functions and ultimately cause bacterial death [35]. Table 1 summarizes the experimental synthesis and antibacterial results corresponding to Figure 1, Figure 2 and Figure 3 [34,35].

**Figure 4:**
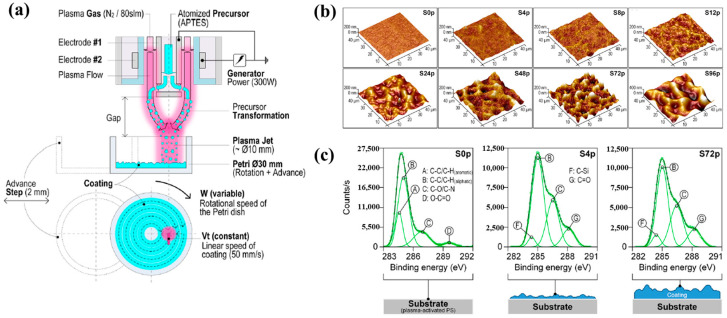
(**a**) APPJ system for pAPTES polymer film deposition with rotational pattern by Sainz–García et al. (**b**) AFM images and (**c**) XPS C 1s high-resolution spectra of prepared samples. Reproduced with copyright permission from Ref. [12].

Sainz–García et al. [12] investigated the adhesion of bacterial samples on plasma-polymerized polymer film coatings prepared by using an atmospheric pressure plasma jet (APPJ) on polystyrene (PS) Petri dishes with (3–aminopropyl)triethoxysilane (APTES). Figure 4a illustrates the plasma polymerization process with rotational patterns generated by the APPJ system which consists of two coaxial electrodes. Plasma is generated due to the gas flow between the electrodes, with the internal electrode grounded and the external electrode excited by a high–voltage power source at 68 kHz. The vaporized precursor is injected into the internal electrode within the plasma reactor, where it undergoes plasma-induced reactions and is subsequently deposited as polymer material during multiple plasma passes with APTES. As shown in Figure 4b, increasing the number of plasma polymerization passes transforms the pAPTES film surface morphology from smooth to rough, resulting in a granular topographical pattern with high surface roughness. Figure 4c presents the high–resolution C 1s peaks in the XPS spectra for different polymer films (S0p, S4p, and S72p). For plasma-treated PS surfaces, the uncoated sample (S0p) shows carbon-related functional groups inherent to PS, including C–C, C–H, C–O/C–N, C–O, and O–C=O. In contrast, the plasma–treated PS substrate and plasma-deposited pAPTES films exhibit oxygenated carbon functional groups, namely, C–O, O–C=O, and C=O. Comparison of the XPS spectra confirms that, relative to the uncoated PS Petri dish (S0p), the deposited pAPTES films (S4p, and S72p) introduce additional oxygenated carbon functional groups onto the plasma-treated PS surfaces [12].

**Figure 5:**
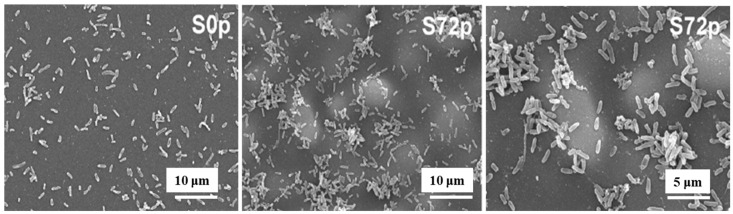
SEM images of sessile bacteria on pAPTES film surfaces of samples (S0p and S72p). Reproduced with copyright permission from Ref. [12].

Figure 5 shows SEM images of sessile bacteria on deposited pAPTES film surfaces for different samples (S0p and S72p). When inoculated with the same initial bacterial concentration on uncoated (S0p) and coated (S72p) samples, a higher density of sessile bacteria accumulated in the valley structures of the pAPTES film, which provided a large surface area and rough morphology. Because the plasma-deposited polymer film was formed from an APTES precursor, it contained amine (–NH_2_) functional groups, which became positively charged due to their ability to accept protons. As a result, negatively charged bacterial (*P. aeruginosa*) cells were electrostatically attracted to these amine groups and attached to them. Thus, both the chemical functionality and morphological characteristics of plasma–deposited polymer films play a critical role in enhancing high–density bacterial cell adhesion [12]. Table 2 summarizes the experimental plasma synthesis and bacterial results corresponding to Figure 4 and Figure 5.

**Figure 6:**
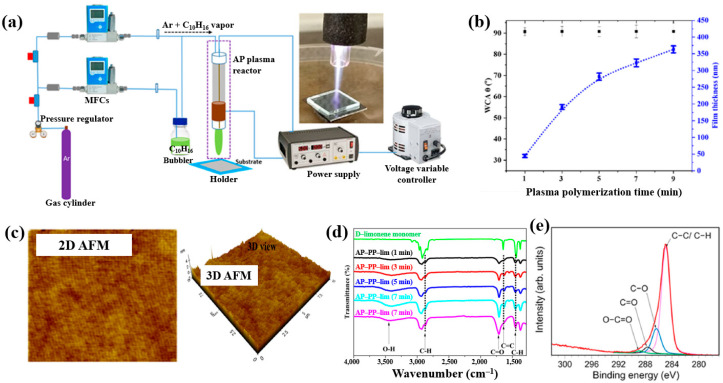
(**a**) Schematic of APP polymerization system by Masood et al. (**b**) Average water contact angle (WCA) values after different plasma polymerization durations. (**c**) 2D and 3D atomic force microscopy (AFM) images of AP–PP–lim film deposited for 1 min. (**d**) FT–IR spectra of D–limonene monomer and AP–PP–lim films deposited for different plasma durations. (**e**) XPS C 1s high-resolution spectra of AP–PP–lim films. Reproduced with copyright permission from Ref. [36].

Masood et al. [36] investigated plasma polymerization under AP conditions using a D–limonene monomer to deposit polymer films for antibacterial applications. Figure 6a schematically depicts the AP plasma polymerization system, in which a copper (Cu) rod electrode was inserted into a quartz tube and connected to a high–voltage (HV) supply. A Cu metal ring, serving as the outer electrode, was positioned around the quartz tube and electrically grounded. Plasma discharge within the plasma jet was generated by applying a voltage of 3 kV. The quartz tube had inner and outer diameters of 3.0 mm and 5.0 mm, respectively. The distance between the HV electrode tip and the nozzle was fixed at 20 mm, with the nozzle positioned 15 mm above the glass substrate. AP plasma-polymerized D–limonene (AP–PP–lim) films were deposited on 5 cm × 5 cm glass substrates using vaporized D–limonene carried by argon (Ar) gas at a flow rate of 130 sccm [36]. Figure 6b shows the average water contact angle (WCA) of AP–PP–lim films deposited for different plasma polymerization durations along with images of water droplets on control and coated surfaces. Film thickness measured using a surface profiler increased with plasma exposure times (1, 3, 5, 7, and 9 min), corresponding to a deposition rate of 0.8 nm/s. The control glass substrate exhibited a hydrophilic surface with a very low WCA, whereas all AP–PP–lim–coated samples treated for more than 1 min displayed high WCA values exceeding 90.7°. Notably, the WCA of the AP–PP–lim films remained constant beyond this point [34]. Additionally, the atomic force microscopy (AFM) results in Figure 6c show that an AP–PP–lim film deposited for 1 min exhibited a smooth surface with an average roughness (R_a_) of 0.23 nm and a root mean square roughness (R_q_) of 0.27 nm. The chemical structure of the D–limonene monomer and AP–PP–lim films was analyzed using FT–IR spectroscopy. As shown in Figure 6d, the FT–IR spectrum of D–limonene displays characteristic peaks corresponding to symmetric C−H stretching (2834 and 2856 cm^−1^), asymmetric C–H stretching (2920 and 2964 cm^−1^), unsaturated C–H bonds (3010, 3046, 3072, and 3083 cm^−1^), C=C stretching (1644 cm^−1^), asymmetric C–H bending (1436 and 1451 cm^−1^), symmetric C–H bending (1375 cm^−1^), and out–of–plane C–H bending (1310 cm^−1^). These peaks were retained in the AP–PP–lim film; however, peak broadening and the appearance of additional bands indicates cross-linking induced by plasma polymerization relative to the D–limonene monomer. The chemical composition of the AP–PP–lim film was further examined by XPS, and the C 1s spectrum is presented in Figure 6e. The spectrum contains four components: hydrocarbons bonds (C–C/C–H) of polymer chain and oxygenated carbon groups (C–O–H, C–O–C, C–O, C=O, and O–C=O). The presence of these oxygenated carbon groups is attributed to the partial oxidation of the AP–PP–lim film [36].

**Figure 7:**
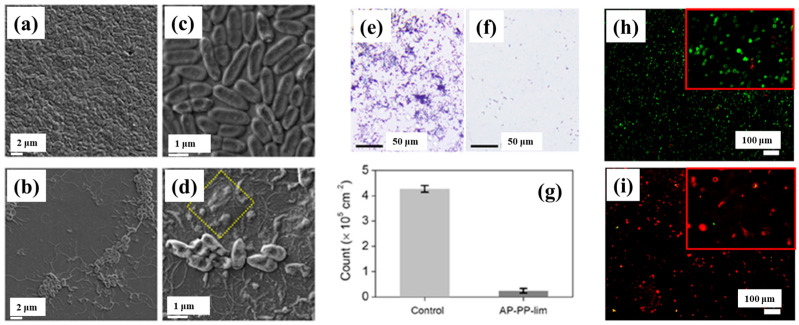
Antimicrobial performance of *E. coli* on AP–PP–lim film: (**a**,**c**) FE–SEM images of *E. coli* on control surfaces. (**b**,**d**) FE–SEM images on AP–PP–lim films. (**e**,**f**) Fluorescence microscopy of crystal violet–stained *E. coli* on control and AP–PP–lim films. (**g**) Bacterial counts on control and AP–PP–lim films. (**h**,**i**) Live–dead fluorescence assay images of *E. coli* adhered to glass and AP–PP–lim films. Reproduced with copyright permission from Ref. [36].

To verify the antibacterial performance of the film, the morphology of *E. coli* attached to control and AP–PP–lim films was examined using field emission scanning electron microscopy (FE–SEM). Figure 7a–d show FE–SEM images of *E. coli* on the uncoated sample (control) and AP–PP–lim films [36]. In Figure 7a,c, rod–shaped *E. coli* with lengths of approximately 2 μm and widths of 0.46 μm are observed on the control sample. In contrast, Figure 7b,d, reveal withered and isolated *E. coli* on the AP–PP–lim film, with a substantial reduction in bacterial colonies, and dead cells are clearly visible within the yellow box in Figure 7d. The antibacterial effect was further assessed using fluorescence microscopy. Figure 7e,f present fluorescence images of treated *E. coli* on control and AP–PP–lim films after 24 h of incubation, while Figure 7g quantifies a reduction in bacterial adhesion exceeding 94% on the AP–PP–lim film compared to the control. Likewise, Figure 7h,i show fluorescence images of E. coli attached to a glass substrate and an AP–PP–lim film following 24 h of cultivation, confirming a markedly lower number of attached bacteria on the AP–PP–lim film [36]. The antibacterial properties of plasma–deposited films are attributed primarily to the hydrocarbon groups (C–C, C–H) from the D–limonene monomer and the oxygenated carbon groups (C–O, C=O, and O–C=O) generated through free radical reactions under plasma conditions in ambient air. Table 3 summarizes the experimental synthesis and antibacterial results corresponding to Figure 6 and Figure 7 in this section.

**Figure 8:**
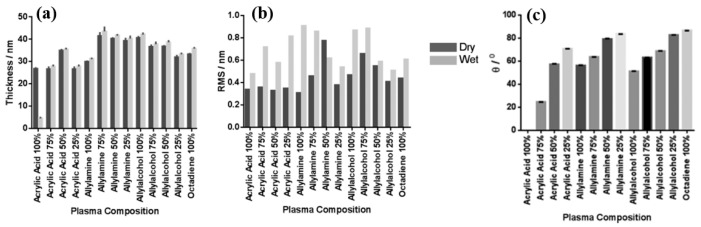
(**a**) Film thickness, (**b**) surface roughness, and (**c**) WCA of deposited PP films under dry and wet conditions. Reproduced with copyright permission from Ref. [37].

Smith et al. investigated plasma polymerization and copolymerization to prepare plasma polymer (PP) surfaces from four organic monomers: acrylic acid (ppAAc), allyl amine (ppAAm), allyl alcohol (ppAAOH), and copolymers with the diluent hydrocarbon 1, 7, octadiene, (ppOD) [37]. In this study, PP layers of AAc and AAOH were deposited using a radio frequency (13.56 MHz) plasma generator under varying power conditions (3 W for AAc, AAOH, and OD, and 5 W for AAm). All deposited PP layers, except ppAAc, were sufficiently thick [37]. As shown in Figure 8a,b, all monomers exhibited a fast deposition rate and the resulting films displayed smooth surfaces with a roughness (R_q_) of less than 1 nm [37]. The thickness of the PP film formed at a deposition rate of 1.3 nm/min using AAc increased with higher concentrations of octadiene in the plasma.

Similarly, the thickness of the PP film formed at a rate of 1 nm/min using AAm also increased with increasing OD content. In contrast, although the initial deposition rate using AAOH was 2 nm/min, the resulting PP film thickness decreased when octadiene was introduced into the plasma. Contact angle measurements (Figure 8c) indicated that the ppAAc–OD film exhibited a hydrophilic surface. When the AAc monomer ratio decreased from 75% to 25%, the contact angle increased from 25° to 72°. Additionally, the contact angles for ppAAm and ppAAOH surfaces were measured at 56° and 51°, respectively. As the octadiene content increased, the PP films became more hydrophobic due to a reduction in hydrophilic functional groups. The pure ppOD polymer film exhibited a hydrophobic surface with a contact angle of 86.5° [37].

**Figure 9:**
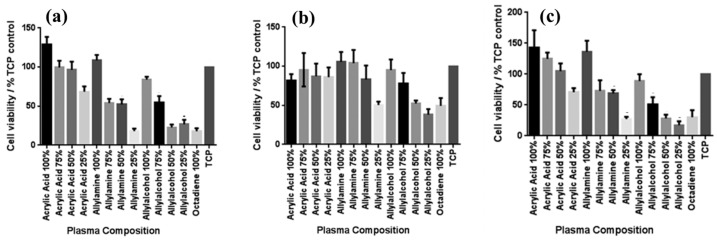
Cell viability on PP surfaces after incubation using the MTT–ESTA assay with (**a**) keratinocytes, (**b**) fibroblasts, and (**c**) endothelial cells. Reproduced with copyright permission from Ref. [37].

To examine the effect of functional groups and their densities on the viability of various skin cells, including fibroblasts, keratinocytes, and endothelial cells after 4 days in culture, MTT–ESTA assays were performed. The experimental results are presented in Figure 9a–c, showing cell viability on different PP surfaces. In all cases, tissue culture polystyrene (TCP) was used as a reference without a PP layer. As shown in Figure 9a, keratinocyte viability was highest on surfaces with 100% ppAAc and ppAAm. As shown in Figure 9b, fibroblast viability decreased under low functional group concentrations, particularly on 25% ppAAm and ppAAOH surfaces. Similarly, endothelial cells responded in a manner comparable to keratinocytes across the PP surfaces. (Figure 9c), showing improved viability on ppAAc compared with ppAAm and ppAAOH. In cases of high functional group concentrations (100% AAc, 100% AAm, and 100% AAOH), all three cell types exhibited enhanced growth on PP surfaces. Conversely, endothelial cell viability decreased with decreasing functional group concentration [37]. Table 4 summarizes the experimental plasma synthesis parameters and biological results corresponding to Figure 8 and Figure 9 in this section.

**Figure 10:**
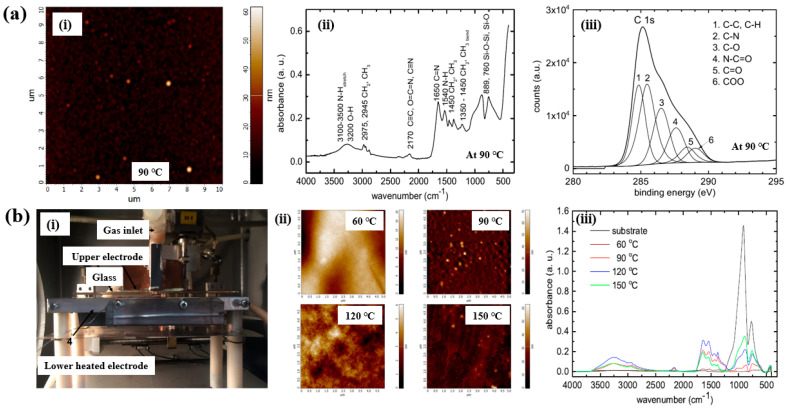
(**a**(**i**)) AFM image of POx thin film, (**a**(**ii**)) FT–IR spectrum, and (**a**(**iii**)) high–resolution XPS C 1s spectrum of POx thin films deposited at 90 °C by Mazánková [23]. (**b**(**i**)) Photo image of plasma deposition setup; (**b**(**ii**)) AFM images; (**b**(**iii**)) FT–IR spectra of POx thin films deposited at 60, 90, 120, and 150 °C by St’ahel et al. Reproduced with copyright permission from Refs. [21,38].

As shown in Figure 10a,b, Mazánková and St’ahel et al. examined polyoxazoline (POx) thin films produced by AP Townsend–like discharge (APTD) polymerization on glass substrates using 2–ethyl–2–oxazoline monomers as precursors [21,38]. The plasma device used by Mazánková and St’ahel et al. for POx deposition is shown in Figure 10b(i). The POx thin films were synthesized via a DBD–type plasma under AP with an N_2_ gas flow. As shown in Figure 10a(i), the surface roughness (R_q_ and R_a_) of the POx thin films was measured to be approximately 3.2 nm and 2.1 nm, respectively [21]. Figure 10b(ii) shows that the surface morphology of the POx films changed with increasing substrate temperatures (60, 90, 120, and 150 °C) [21]. The FT–IR spectra of the POx films in Figure 10a(i),b(iii) reveal a broad absorption band in the 3000–3600 cm^−1^ range, corresponding to overlapping signals from OH, NH and NH_2_ bonds. Peaks at 2975, 2945, 1450, and 1370 cm^−1^ are associated with CH_3_ and CH_2_ stretching and bending vibrations. The peak at 2170 cm^−1^ is attributed to C≡C, O=C=N, or C≡N functionalities. The absorption band between 1790 and 1590 cm^−1^ corresponds to the C=N bond of the oxazoline ring, while the peak at approximately 1550 cm^−1^ indicates N–H bonding. These FT–IR results confirm the formation of various functional groups through fragmentation and recombination reactions of oxazoline monomers during plasma polymerization [21,38]. Furthermore, as shown in Figure 10a(iii), XPS analysis of the POx film revealed six deconvoluted peaks corresponding to the following bonding states: C–C/C–H (284.7 eV), C–N (285.4 eV), C–O (286.3 eV), N–C=O (287.7 eV), C=O (288.2 eV), and COO (289.1 eV) [21].

**Figure 11:**
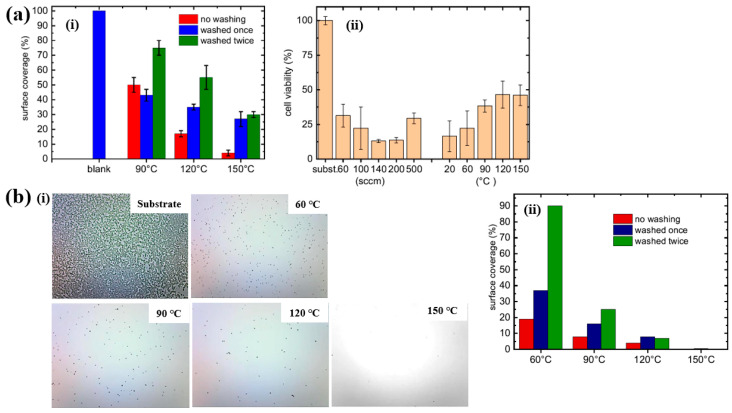
(**a**(**i**)) Surface coverage (%) of *S. epidermidis* on POx thin films at different substrate temperatures. (**a**(**ii**)) In vitro cytocompatibility for POx thin films prepared under varying substrate temperatures and monomer flow rates by Mazánková et al. [21]. (**b**(**i**)) Imges of POx thin films exposed to *S. epidermidis*. (**b**(**ii**)) Surface coverage (%) of *S. epidermidis* on POx thin films deposited at different substrate temperatures by St’ahel et al. Reproduced with copyright permission from Refs. [21,38].

Based on the experimental results in Figure 11a,b, the antibacterial and in vitro cytocompatibility properties of POx thin films were investigated under varying substrate temperatures and monomer vapor flow rates [21,38]. Figure 11a(i) shows that the surface coverage area (%) of *S. epidermidis* on POx thin films deposited at 90 °C was approximately 50%. In vitro cytocompatibility tests were conducted over 48 h using mouse embryonic fibroblast (NIH/3T3) cells cultured on POx thin films deposited at different substrate temperatures and monomer flow rates. According to Figure 11a(ii), cell viability on POx thin films was significantly lower than that on the pure glass substrate [21].

St’ahel et al. further evaluated the antibacterial performance of POx thin films against S. epidermidis, focusing on the influence of deposition temperature (pure substrate, 60, 90, 120, and 150 °C) [38]. The optical images in Figure 11b(i) reveal that the pure glass substrate was fully covered by bacterial colonies, whereas the POx–coated surfaces exhibited minimal bacterial adhesion, with only isolated cells or small clusters observed. Figure 11b(ii) quantifies the bacterial surface coverage on POx thin films deposited at different substrate temperatures. The POx films deposited at 60 and 90 °C displayed partial water solubility, resulting in reduced antibiofouling performance after water washing. In contrast, POx thin films deposited at 120 and 150 °C were water–insoluble, and their antibiofouling properties remained stable following washing. Furthermore, in vitro cytocompatibility tests were extended to 72 h using NIH/3T3 cells on POx films deposited at various temperatures. The results indicate that oxazoline-based thin films influence cell attachment and that cell viability increases with higher deposition temperatures. Table 5 presents a summary of the plasma synthesis parameters and antibacterial test results corresponding to Figure 10 and Figure 11 in this section.

**Figure 12:**
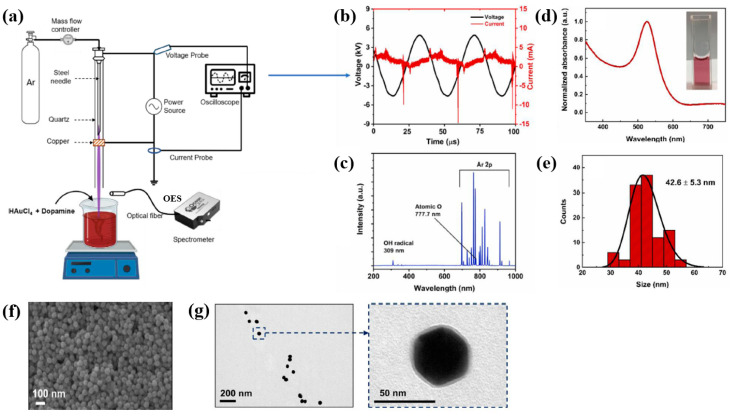
(**a**) Schematic of plasma jet setup for Au NP/polydopamine (PDA) synthesis in liquid solution. (**b**) Current–voltage profile and (**c**) optical emission spectroscopy (OES) spectrum of Ar plasma jet. (**d**) UV–vis absorbance spectrum and (**e**) size distribution of plasma–treated solution containing Au NPs/PDA. (**f**) SEM and (**g**) TEM images of Au NP/PDA nanocomposites. Reproduced with copyright permission from Ref. [39].

Plasma treatment was conducted using a plasma jet setup consisting of a stainless steel needle electrode and a quartz tube, as illustrated in Figure 12a [39,40]. The 12 cm long needle electrode was centrally positioned within a 20 cm long quartz tube, and a high voltage (HV) was applied. A Cu layer was attached to the exterior of the quartz tube, positioned 1 cm below the needle tip, and grounded for stabilization. Argon (Ar) gas was introduced at a flow rate of 700 sccm to generate plasma discharge. Prior to plasma synthesis, a 2 mL aqueous solution containing 0.2 mM H[AuCl_4_]·and 0.05 mM dopamine hydrochloride was prepared in deionized water. This solution was exposed to the plasma jet for 5 min, resulting in the formation of Au NPs/polydopamine (PDA) [39,40]. Figure 12b,c show the current–voltage waveform and the optical emission spectroscopy (OES) spectrum recorded during plasma treatment. An alternating current (AC) with a bipolar voltage, a peak amplitude of 4.8 kV, and a frequency of 28 kHz was applied to the needle electrode. The OES spectrum revealed reactive species, including Ar (700–1000 nm), hydroxyl radicals (309 nm), and atomic oxygen (777 nm). These species interacted with the solution surface during plasma exposure, facilitating the formation of Au NPs/PDA. Following plasma treatment, the Au NPs/PDA were collected by centrifugation at 8000 rpm for 10 min and stored at 4 °C. Figure 12d presents the UV–Vis absorbance spectrum of the plasma-treated HAuCl_4_–DA solution. After 5 min of plasma exposure, the solution color changed from colorless to red wine, indicating the formation of Au NPs/PDA. A strong absorbance peak at approximately 525 nm was observed, corresponding to the SPR of the Au colloids [39]. The average particle size was determined to be 42.6 nm from SEM images using ImageJ software (ver. 1.54) (Figure 12e). The morphology of the synthesized Au NPs/PDA was characterized by SEM and TEM. As shown in Figure 12f, the NPs exhibited spherical morphology, and a 4 nm thick PDA coating layer was observed on the outer shell of each particle (Figure 12g) [39,40].

**Figure 13:**
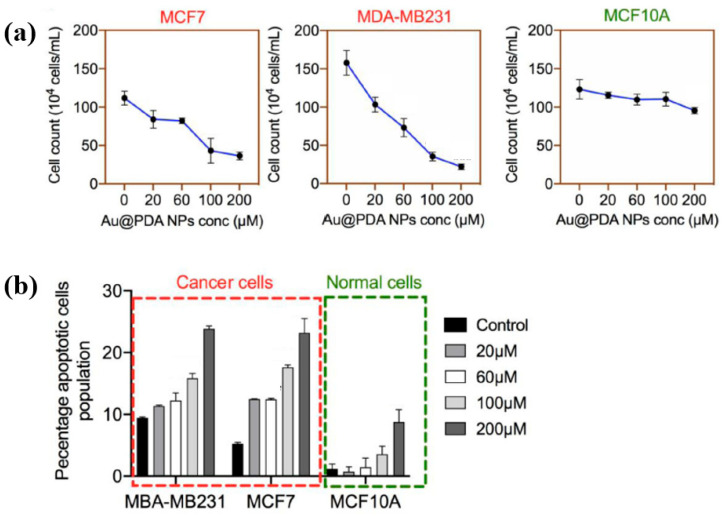
(**a**) Cell counts of breast cancer and normal cells exposed to plasma–treated Au NP/PDA solutions at 20, 60, 100, and 200 mM. (**b**) The total apoptotic population in each cell type as a function of plasma–treated solution concentration. Reproduced with copyright permission from Ref. [39].

To evaluate the impact of Au NPs/PDA on cancer cell growth inhibition, four concentrations (20, 60, 100, and 200 mM) of the plasma–treated solution were applied to breast cancer cell lines (MCF7 and MDA–MB231) and normal epithelial cells (MCF10A). As shown in Figure 13a, cancer cell viability decreased significantly with increasing concentration, particularly at 200 mM, while the viability of normal cells remained largely unaffected after 48 h of exposure. Additionally, cell death was assessed using annexin–V/propidium iodide (PI) staining across the range of plasma-treated concentrations. As illustrated in Figure 13b, treatment with 200 mM of the plasma-treated solution induced cancer cell death rates of up to 23% and 25% in MCF7 and MDA–MB231 cells, respectively, confirming the cancer cell growth inhibition potential of Au NPs/PDA [39,40]. Table 6 presents a summary of the synthesis conditions and biological results corresponding to Figure 12 and Figure 13 in this section.

**Figure 14:**
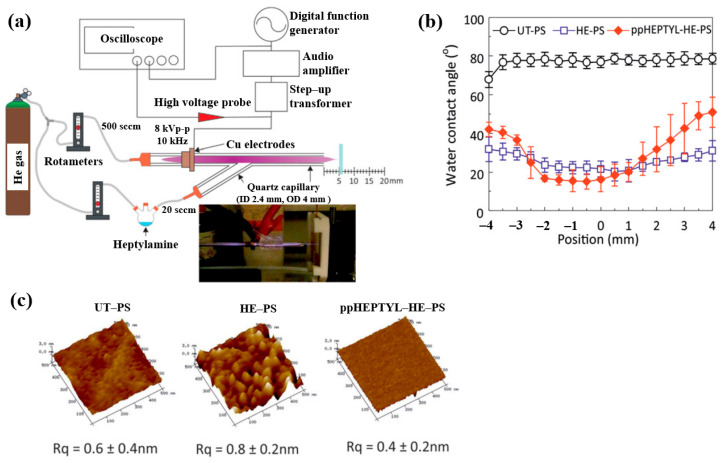
(**a**) Schematic of APPJ experimental setup for heptylamine monomer by Doherty et al. [38]. (**b**) WCA results and (**c**) AFM images with surface roughness (R_q_) of UT–PS, HE–PS, and ppHEPTYL–HE–PS. Reproduced with copyright permission from Ref. [41].

To inject N_2_ species into a polymer surface for cell growth, this study investigated the plasma polymerization of a heptylamine monomer as a precursor using an APP jet (APPJ) in air [41]. Figure 14a shows the APPJ experimental setup by Doherty et al. [41]. A sinusoidal voltage of 8 kV_p–p_ at a fixed frequency of 10 kHz was applied to the electrode to generate plasma, while He gas was supplied at 500 sccm. The liquid heptylamine was vaporized at RT by a He carrier gas flow of 20–100 sccm and subsequently introduced into the APPJ. A polystyrene (PS) substrate of 10 × 10 mm^2^ was positioned 5 cm from the nozzle and treated for 10 min under different conditions, such as untreated PS (UT–PS), He-treated PS (HE–PS), and plasma-polymerized heptylamine (ppHEPTYL–HE–PS) on He-treated PS. As shown in Figure 14b, the UT–PS surface exhibited an average water contact angle (WCA) of 77.2°, whereas both HE–PS and ppHEPTYL–HE–PS displayed significantly lower WCA values, ranging from 20.3° to 40° across all measured positions, indicating enhanced hydrophilicity. The surface roughness (R_q_) of all samples (UT–PS, HE–PS, and ppHEPTYL–HE–PS) remained comparable, in the range of 0.4 nm to 0.8 nm, as shown in Figure 14c [41].

**Figure 15:**
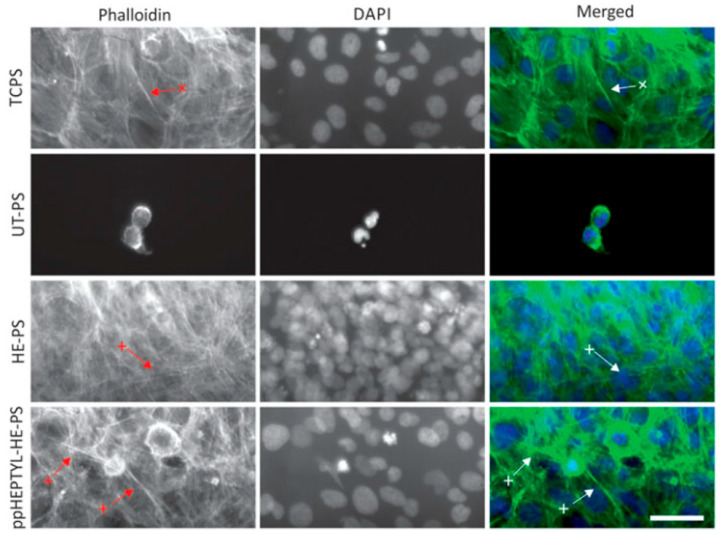
Optical images of human B3 lens epithelial cells (LECs) cultured for 7 d on TCPS, UT–PS, He-treated PS, and heptylamine–treated PS. Reproduced with copyright permission from Ref. [41].

For cell cultivation, human B3 lens epithelial cells (LECs) were grown on various samples, including tissue culture PS (TCPS), UT–PS, HE–PS, and ppHEPTYL–HE–PS. Figure 15 shows optical images of LECs on TCPS, UT–PS, HE–PS, and ppHEPTYL–HE–PS after 7 d. Cells cultured on all samples, except UT–PS, exhibited a fibrotic morphology [41]. Table 7 summarizes the experimental synthesis and biological results corresponding to Figure 14 and Figure 15 in this section.

**Figure 16:**
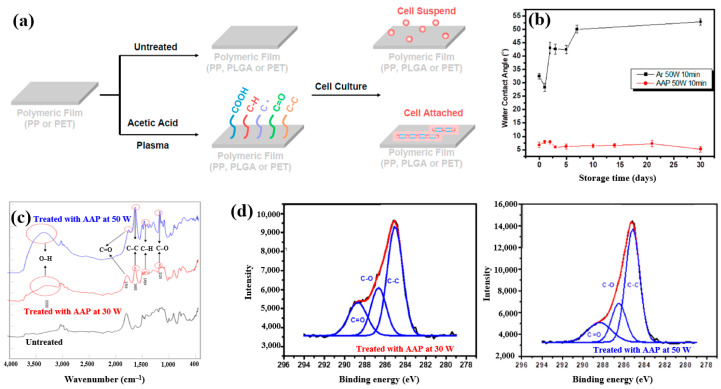
(**a**) Schematic of polymeric film preparation on plasma–treated polyethylene terephthalate (PET) using acetic acid plasma (AAP) with poly(DL–lactide–co–glycolide) (PLGA) coating and subsequent cell culture, adapted from Liao et al. [39]. (**b**) WCA measurements of untreated and AAP–treated films at 30 W and 50 W for 10 min. (**c**) FT–IR spectra of same films; (**d**) XPS C 1s high-resolution spectra of AAP–deposited films at 30 W and 50 W for 10 min. Reproduced with copyright permission from Ref. [42].

Plasma polymerization was performed in a vacuum chamber using radio frequency (RF) at 13.56 MHz. To introduce carboxylic acid groups onto polyethylene terephthalate (PET), an acetic acid monomer was injected and deposited through CAP proposed by Liao et al. [42]. Figure 16a shows a schematic of polymeric film preparation on plasma-treated PET using acetic acid plasma (AAP), followed by cell cultivation. AAP–deposited films were produced by applying plasma for 10 min at three different powers, namely 10, 30, and 50 W. The untreated PET exhibited an initial WCA of 67.5°. After AAP and Ar plasma treatments at 50 W for 10 min, the WCA decreased to 8.2° for AAP and 32° for Ar. Figure 16b shows the variation in WCA of AAP–deposited poly(DL–lactide–co–glycolide) (PLGA) films with storage time. While the WCA of Ar plasma–deposited films increased over time, that of AAP–deposited PLGA films remained stable. FT–IR spectra of PLGA treated with AAP at 30 and 50 W are shown in Figure 16c, where several peaks associated with acetic acid polymer can be detected. As plasma power increased, the FT–IR peaks corresponding to functional groups in AAP-deposited PLGA films became stronger. Figure 16d presents the XPS high–resolution C 1s spectra of AAP–deposited PLGA films treated for 10 min at 30 and 50 W. In all cases, the spectra consisted of three peaks corresponding to C–C, C–O, and C=O functional groups. Notably, the relative intensity of C=O increased as plasma power decreased [42]. These oxygen–containing functional groups impart hydrophilic properties to the AAP–deposited film surface.

**Figure 17:**
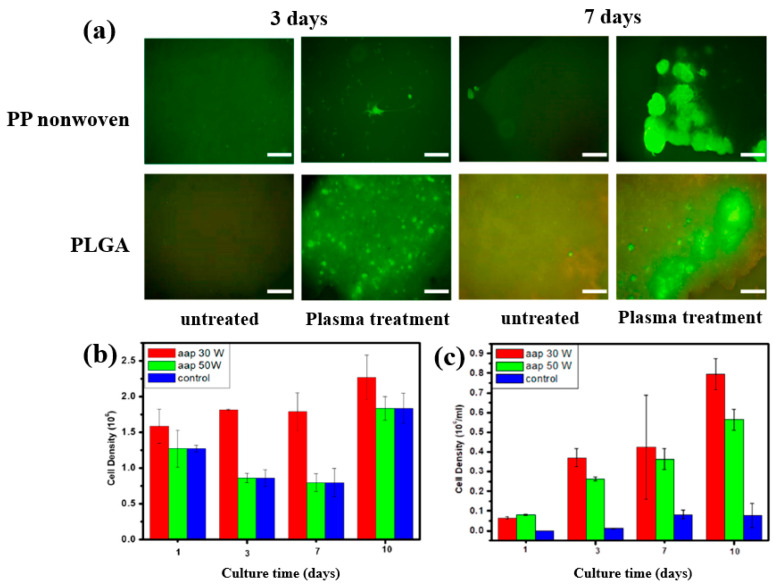
(**a**) Fluorescence microscopy images of embryonic stem (ES) cells cultured for 3 and 7 d on PP nonwoven substrates and AAP–deposited PLGA films with or without AAP treatment. Cell density of ES cells on (**b**) PP nonwoven substrate and (**c**) PLGA films treated with 30 and 50 W, evaluated at 1, 3, 7, and 10 d. Reproduced with copyright permission from Ref. [42].

To further investigate cell adhesion on AAP–deposited films, mouse embryonic stem (ES) cells were cultivated for 3 and 7 d on PP nonwoven substrates and AAP–deposited PLGA films. Figure 17a shows fluorescence microscopy images of cells grown on PP and PLGA substrates with and without AAP treatment. ES cells adhered poorly to the untreated PP and PLGA, whereas they exhibited strong adhesion on the AAP–deposited PLGA film. The attached cells were uniformly distributed and proliferated across the PLGA surface. As shown in Figure 17b,c, the measured cell density confirmed significantly higher proliferation on the AAP–deposited PLGA film compared to untreated PP and PLGA substrates [42]. These enhancements in adhesion and proliferation are attributed to increased surface hydrophilicity induced by oxygen–containing functional groups introduced during AAP treatment. Table 8 summarizes the experimental synthesis and biological results corresponding to Figure 16 and Figure 17 in this section.

**Figure 18:**
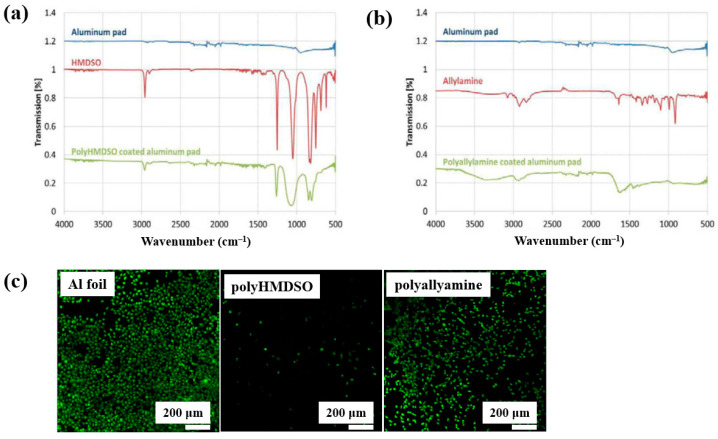
FT–IR spectra of plasma–deposited (**a**) polyHMDSO and (**b**) polyallylamine films on aluminum (Al) foils. (**c**) Confocal microscope images of L929 mouse fibroblasts (green fluorescence) cultured on uncoated aluminum (Al) foil, polyHMDSO films, and polyallylamine films. Reproduced with copyright permission from Ref. [43].

Teske et al. [43] investigated polymer films deposited from allylamine and hexamethyldisiloxane (HMDSO) monomers using plasma–enhanced chemical vapor deposition (PECVD). The films were deposited on aluminum (Al) substrates with an RF generator operating at 13.56 MHz. FT–IR spectra of the polyHMDSO film (Figure 18a) showed characteristic bonding structures in the ranges of 1300–700 and 3000 cm^−1^, while the polyallylamine film (Figure 18b) displayed peaks associated with allylamine in the ranges of 4200–2800 cm^−1^ and 1750–1300 cm^−1^. The adhesion of mouse fibroblast (L929) cells was examined on three samples, namely Al foil, polyHMDSO, and polyallyamine films, using confocal microscopy. As shown in Figure 18c, L929 cells adhered and proliferated on Al foil, while adhesion and growth on polyallyamine films were slightly reduced. In contrast, no cell adhesion was observed on polyHMDSO films [43]. Table 9 summarizes the experimental synthesis and biological results corresponding to Figure 18 in this section.

**Figure 19:**
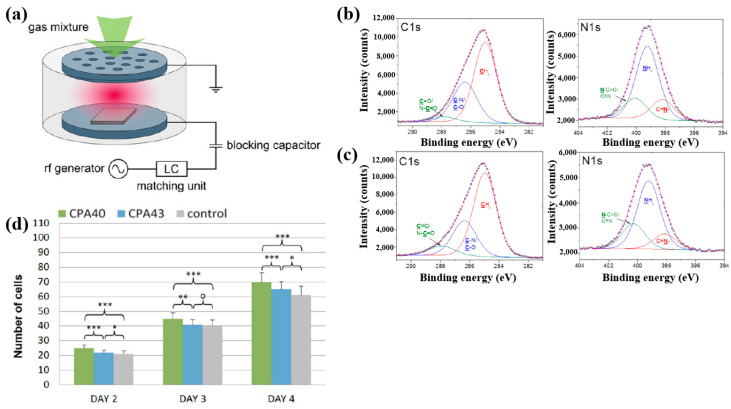
(**a**) Schematic of RF capacitively coupled plasma setup. XPS C 1s and N 1s spectra of CPA40 (**b**) before and (**c**) after immersion in water. (**d**) Cell counts from cultures grown on CPA40, CPA43, and control sample. Reproduced with copyright permission from Ref. [44].

Štrbková et al. [44] investigated the adhesion, viability, and proliferation of cells on amine-rich polymer thin films deposited using RF capacitively coupled plasma with a cyclopropylamine (CPA) precursor, producing films with different N_2_ and amine group (NH_x_) concentrations. Figure 19a shows a schematic of the RF capacitively coupled plasma setup for CPA thin-film deposition under low pressure [44]. XPS analysis (Figure 19b,c) revealed that, after soaking in water, the surface composition of CPA films changed slightly. Oxygen concentration in the CPA40 and CPA43 films increased due to N_2_ loss, leading to enhanced C=O/N–C=O and N–C=O contributions at the expense of C–N/C–O and NHx groups. To assess cell adhesion and growth on plasma-deposited CPA thin films, human dermal fibroblast (HDF) cells were cultured on control, CPA40, and CPA43 samples, and their behavior was observed using coherence-controlled holographic microscopy (CCHM). As shown in Figure 19d, cell adhesion was higher on CPA40 and CPA43 films compared to the control. These results demonstrate that amine-rich CPA thin films are biocompatible, and that cell adhesion and proliferation increase with higher concentrations of amine groups [44]. Table 10 summarizes the experimental synthesis and biological results corresponding to Figure 19a–d in this section.

**Figure 20:**
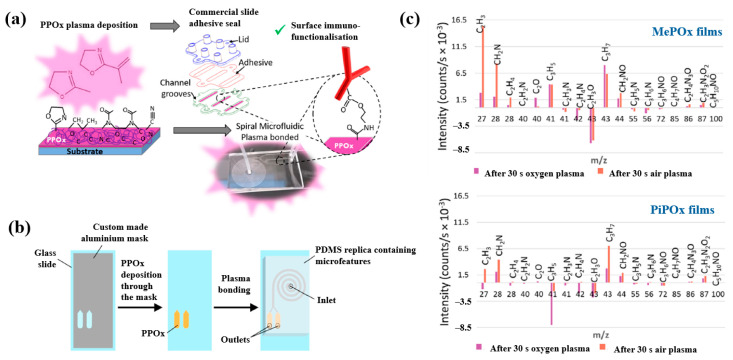
Schematic of (**a**) biosensor fabrication and (**b**) plasma–deposited polyoxazoline (PO_x_) film and device assembly. (**c**) ToF–SIMS spectra showing changes in molecular fragments of MePOx and PiPOx films after O_2_ or air plasma treatment. Reproduced with copyright permission from Ref. [45].

Gheorghiu et al. [45] investigated the chemistry and functionality of plasma–deposited polyoxazoline (POx) films derived from methyl oxazoline (MePOx) and isopropenyl oxazoline (PiPOx), aiming to develop POx–based immunosensors for cancer cell applications. Prior to deposition, substrates were treated with air plasma at 30 W for 3 min. For assembly into spiral microfluidic chips (Figure 20a,b), the POx films were deposited on glass microscope slides at 30 W for 30 and 50 s [45]. The time–of–flight secondary ion mass spectrometry (ToF–SIMS) spectra of MePOx and PiPOx films treated with O_2_ and air plasma are shown in Figure 20c. All POx films contained higher amounts of aliphatic fragments (C_2_H_3_ and C_3_H_7_) and lower amounts of heteroatomic fragments (C_2_H_4_N and C_2_H_3_O). The key distinction between MePO_x_ and PiPO_x_ films was the behavior of the C_3_H_5_ fragment, associated with the pendent group in the PiPOx polymer structure. After plasma treatment, the C_3_H_5_ fragment decreased more significantly in PiPO_x_ under O_2_ plasma, indicating that isopropenyl groups on the PiPOx surface were preferentially removed by ROS compared to air plasma.

**Figure 21:**
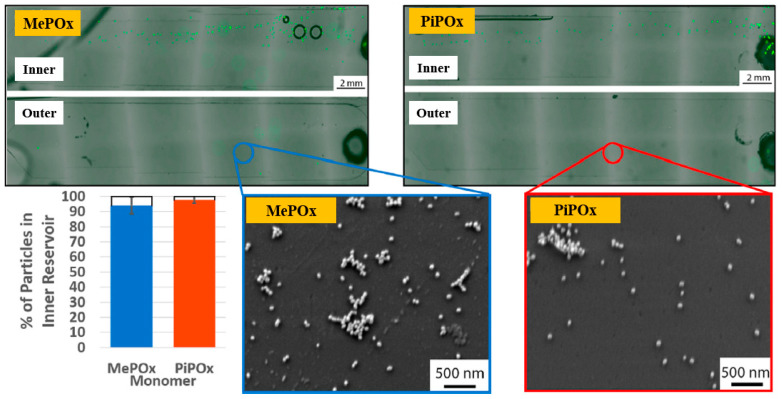
Fluorescence microscopy and SEM images of inner and outer reservoirs functionalized with MePOx and PiPOx films. Reproduced with copyright permission from Ref. [45].

As shown in Figure 21, the reactivity of POx was preserved within the microfluidic reservoirs. Furthermore, both MePOx and PiPOx films directed more than 90% of the microparticles into the inner reservoir [45]. Table 11 summarizes the experimental synthesis and biological results corresponding to Figure 20 and Figure 21 in this section.

**Figure 22:**
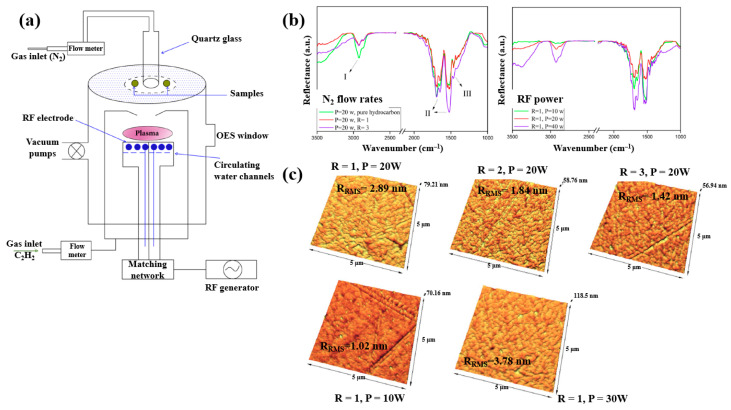
(**a**) Schematic of PE–CVD for L–PPA:N film deposition. (**b**) FT–IR spectra and (**c**) AFM images of plasma–polymerized films prepared with different N_2_ flow rates and RF powers. Reproduced with copyright permission from Ref. [46].

Plasma polymerization was performed in a plasma-enhanced chemical vapor deposition (PE–CVD) reactor with an inductive capacitively coupled electrode by Ghafouri et al., as shown in Figure 22a. The plasma was generated from a mixture of N_2_ gas and an acetylene monomer using an RF of 13.56 MHz [46]. Figure 22b shows the FT–IR spectra of L–PPA:N films deposited under different conditions of N_2_ flow and RF power. The spectra indicate that the plasma-deposited L–PPA:N films consist of three bonding structures, namely bond I (3000–2800 cm^−1^ C–H stretch), II (1800–1500 cm^−1^ C=C and C=N stretch), and III (1500–1300 cm^−1^ CH_2_ and CH_3_ bending). As shown in Figure 22c, the surface of the L–PPA:N film becomes smoother with increasing N_2_ flow, whereas the surface roughness increases with increasing RF power from 10 to 30 W due to the etching effect caused by the higher concentration of reactive plasma species [46].

**Figure 23:**
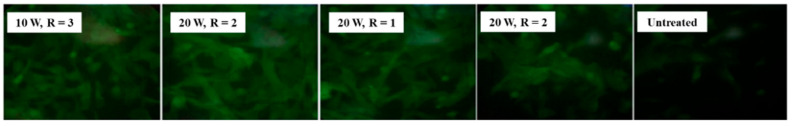
Fluorescence images of stem cells after 1 week of cultivation on plasma–treated samples. Reproduced with copyright permission from Ref. [46].

Figure 23 shows fluorescent images of stem cells after 1 week of cultivation on different plasma-treated samples [46]. The cells are well attached and proliferating on the plasma–treated surface compared with the untreated sample. Table 12 summarizes the experimental synthesis conditions and bacterial results corresponding to Figure 22 and Figure 23 in this section.

**Figure 24:**
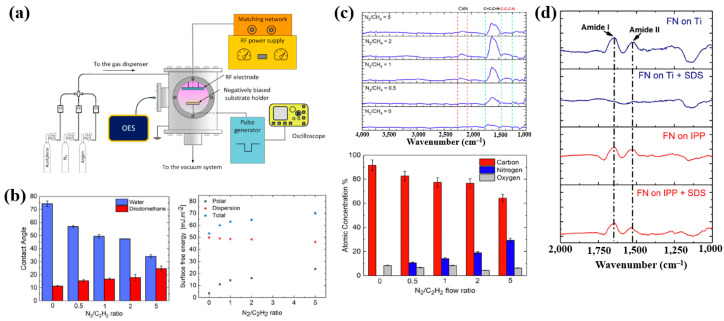
(**a**) Experimental setup of ion–assisted plasma polymerization (IPP) system for acetylene polymer film deposition. (**b**) WCA and surface energy, (**c**) FT–IR spectra, and (**d**) XPS composition of IPP films prepared with different N_2_/C_2_H_2_ flow ratios. Reproduced with copyright permission from Ref. [47].

Sharifahmadian et al. [47] investigated ion-assisted plasma polymer (IPP) films containing N_2_ species for the biofunctionalization of implantable devices. Figure 24a shows the experimental setup for IPP film deposition from a mixture of N_2_ and acetylene gases. Deposition was carried out at an RF power of 50 W with varying N_2_ concentrations and N_2_/C_2_H_2_ ratios. As shown in Figure 24b, the WCA decreased with increasing N_2_/C_2_H_2_ gas flow ratio. To evaluate the effect of this ratio on the chemical composition and structure of IPP films, FT–IR and XPS analyses were performed. Figure 24c shows FT–IR spectra with peaks in the ranges of 1200–1500, 1500–1800, and 2100–2250 cm^−1^. The peak at 1200–1500 cm^−1^ is attributed to C–C and C–O vibrations, while the broad peak at 1500–1800 cm^−1^ corresponds to C=C, C=O, C=N, or N–H vibrations, and this band broadened with increasing N_2_/C_2_H_2_ ratio. These spectral changes are attributed to the enhanced formation of CN radicals during plasma deposition under higher N_2_/C_2_H_2_ ratios. The X-ray photoelectron spectroscopy (XPS) results in Figure 24d show that the N_2_ concentration increased from 0 to 29.4% as the N_2_/C_2_H_2_ ratio increased from 0 to 5, while the carbon concentration decreased from 91.6 to 64.3% [47].

**Figure 25:**
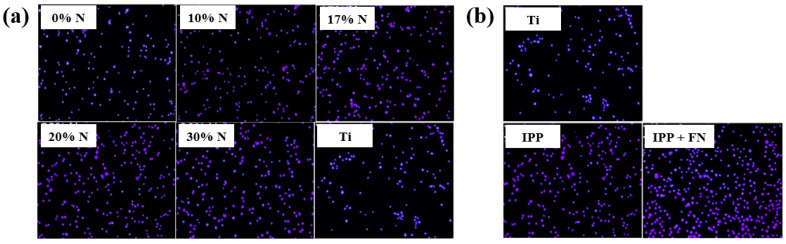
Fluorescence images of osteoblasts on (**a**) IPP films prepared with different N_2_ concentrations and (**b**) after 1 h cultivation on bare Ti, IPP, and IPP with FN films. Reproduced with copyright permission from Ref. [47].

To evaluate cell adhesion on the deposited IPP films, osteoblast cells were seeded onto films prepared with different N_2_ concentrations and examined by fluorescence optical microscopy. As shown in Figure 25a,b, cell attachment and proliferation were enhanced on both the IPP film and the IPP film with FN compared with the Ti substrate [47]. Table 13 summarizes the experimental synthesis conditions and bacterial results corresponding to Figure 24 and Figure 25 in this section.

**Figure 26:**
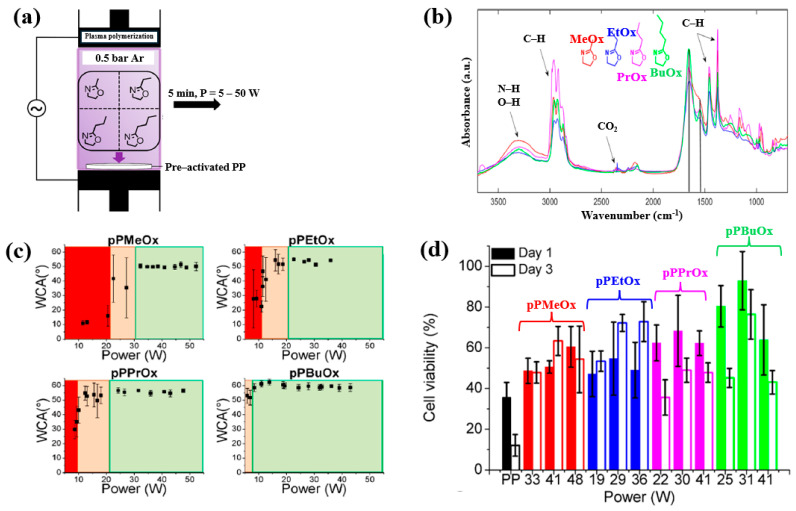
(**a**) Experimental setup of plasma–polymerized film deposition using four monomers (MeO_x_, EtO_x_, PrO_x_, and BuO_x_). (**b**) FT–IR spectra and (**c**) WCA results of four plasma–polymerized films. (**d**) Cell viability of human foreskin fibroblast (HFF) cells after 1 and 3 d of cultivation on four polymer films. Reproduced with copyright permission from Ref. [48].

Van Guyse et al. [48] investigated the chemical structure and cell viability of polymer films prepared by using the LPP–DBD plasma process with four monomers, namely 2–methyl–2–oxazoline (MeO_x_), 2–ethyl–2–oxazoline (EtO_x_), 2–n–propyl–2–oxazoline (PrO_x_), and 2–n–butyl–2–oxazoline (BuO_x_). The plasma polymer films were deposited on polypropylene (PP) substrates in a DBD reactor, as shown in Figure 26a. This cylindrical reactor consists of two electrodes separated by a gap of 8 mm. The bottom Cu electrode is coated with a ceramic material, while the upper electrode is a woven stainless steel electrode. The bottom electrode is connected to a 50 kHz AC power supply, and the upper electrode is grounded [48]. The chemical structure and hydrophilicity of polymer films were investigated by using WCA and FT–IR measurements. As shown in Figure 26b, all polymer films exhibited similar FT–IR peaks at 3500–3000 cm^−1^ (N−H or O−H stretching), 3000–2800 cm^−1^ (C−H stretching), and 1700–1500 cm^−1^ (C=O stretching or N−H bending). Figure 26c shows the WCA results as a function of power for the four monomers. The WCAs were approximately 50° for pPMeO_x_, 54° for pPEtO_x_, 56° for pPPrO_x_, and 60° for pPBuO_x_. Figure 26d presents the adhesion and viability of human foreskin fibroblast (HFF) cells after 1 and 3 d of cultivation on the four polymer films. The pristine PP substrate exhibited low cell viability, whereas all plasma polymer films enhanced cell adhesion. The results suggest that cell adhesion strength increases with polymer chain length [48]. Table 14 summarizes the experimental synthesis conditions and bacterial results corresponding to Figure 26 in this section.

**Figure 27:**
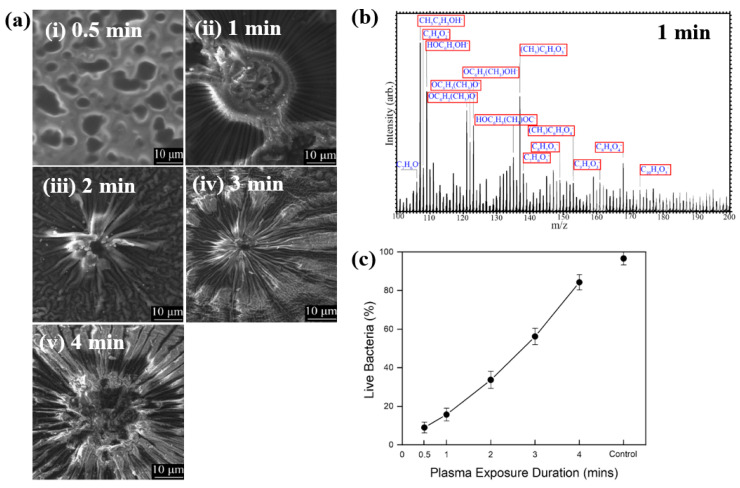
(**a**) FE–SEM images of M–cresol films on Si wafers at plasma exposure times of (i) 0.5, (ii) 1, (iii) 2, (iv) 3, and (v) 4 min. (**b**) ToF–SIMS spectrum of M–cresol film deposited by plasma for 1 min. (**c**) Percentage of live E. coli cells on plasma–synthesized M–cresol films with different plasma treatment durations. Reproduced with copyright permission from Ref. [49].

Hartl et al. [49] investigated the structure and bacterial cell response to 3–methylphenol (M–cresol) films prepared by an AP DBD–plasma process on silicon substrates under RT conditions. To form the M–cresol films, the Si (silicon) substrate was positioned 7 mm below the top of the plasma reactor. The films were deposited in open air with plasma exposure times of 0.5, 1, 2, 3, and 4 min. Plasma discharge was applied with a peak–peak voltage of 30 kV at a frequency of 40 kHz. Figure 27a shows FE–SEM images of the deposited M–cresol films on Si wafers after different plasma durations. At 0.5 min, the films exhibited a smooth morphology, whereas with increasing plasma time, a sunburst pattern developed, and the number of sunbursts increased progressively. The chemical composition of the M–cresol film synthesized after 1 min of plasma exposure was analyzed using ToF–SIMS. The spectrum reveals fragment peaks associated with benzene ring, including C_7_H_7_O_2_, C_10_H_5_O_3_, C_8_H_8_O_4_, and C_9_H_5_O_3_. (Figure 27b), suggesting oligomerization or polymerization during plasma synthesis [49].

To evaluate antimicrobial properties, M–cresol films were prepared via plasma synthesis, and a pristine Si substrate served as the control. As shown in Figure 27c, *E. coli* cell survival decreased by approximately 91% after 15 min of seeding on the M–cresol film prepared with 0.5 min plasma treatment. This film exhibited the highest antimicrobial activity, while longer plasma exposure durations resulted in increased survival of live bacterial cells [49]. Table 15 summarizes the experimental synthesis conditions and bacterial results corresponding to Figure 27 in this section.

**Figure 28:**
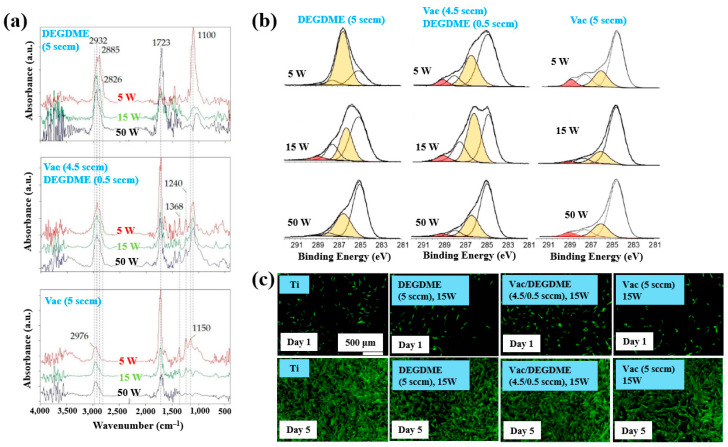
(**a**) FT–IR spectra and (**b**) XPS C 1s spectra of polymer films prepared by using LP method. (**c**) Fluorescence images of NHDF cells attached after 24 h cultivation on bare Ti, DEGDME, VAc, and DEGDME–Vac films. Reproduced with copyright permission from Ref. [50].

Sardella et al. [50] investigated polyethylene oxide (PEO)–like films deposited via low pressure (LP) and aerosol–assisted APP–enhanced chemical vapor deposition (AP PE–CVD) on titanium (Ti) and polycarbonate (PC) substrates. Figure 28a presents the FT–IR spectra of polymer films deposited using the LP method from DEGDME, VAc, and a mixed solution (Vac/DEGDME = 4.5/0.5) under varying plasma power conditions. Based on the FT–IR spectra in Figure 28a, a peak at 1100 cm^−1^ corresponding to ether functional groups, characteristic of PEO, can be observed in polymer films deposited from the DEGDME monomer. These films exhibited structural features similar to PEO polymers, including CH_2_ stretching peaks at 2940–2810 cm^−1^ and a carbonyl peak at 1723 cm^−1^. As the plasma power increased from 5 to 50 W, the intensity of the C–O–C ether peak decreased, while the carbonyl peak intensity increased, suggesting fragmentation or inter–chain crosslinking of polymer chains during plasma deposition. Notably, films formed under lower plasma power and retained structures more closely resembling PEO. In contrast, polymer films deposited from the VAc monomer exhibited CH_3_ peaks at 1240 cm^−1^ and 1368 cm^−1^, along with a C=O stretching peak at 1723 cm^−1^. Films deposited from the mixed monomer solution displayed characteristic peaks of both DEGDME and VAc. As plasma power increased from 5 to 15 W, the intensity of the C=O stretching peak at 1723 cm^−1^ initially decreased, but increased again at 50 W. The XPS results in Figure 28b show that the chemical composition of the deposited PEO–like polymer films was influenced by plasma power and the monomer feed ratio. A higher plasma power resulted in a decrease in the XPS peak associated with PEO–like functional groups, indicating degradation of PEO–like characteristics under harsher plasma conditions.

To assess biocompatibility, polymer films were deposited onto Ti substrates via the LP method using DEGDME, VAc, and the mixed solution. Figure 28c illustrates cell adhesion and growth behaviors on the three different polymer surfaces. After 1 d of cultivation, minimal cell attachment was observed on all polymer surfaces. However, after 5 d, extensive cell coverage was evident across all polymer film surfaces. This enhanced cell growth is attributed due to the presence of CH_2_CH_2_O ester functional groups formed on the surface during LP deposition [50].

Furthermore, the cell adhesion characteristics of polymer samples fabricated by AP PE–CVD were investigated. Cell-repellent films were deposited on PC substrates using aerosol-assisted APP in a DBD reactor, employing a mixture of He and triethylene glycol dimethyl ether (TEGDME). The experiments were conducted using either a single-step or multi-step process to enhance cell adhesion properties. In the single-step process, PEOA was deposited at a He flow rate of 4.85 standard liters per minute (SLM), while PEOB was deposited at a higher He flow rate of 6.85 SLM. In both cases, the He–TEGDME aerosol gas was maintained at 3.15 SLM. The detailed experimental conditions for the three-step AP PE–CVD processes used for PEOA and PEOB deposition are provided in Table 16 [50].

**Figure 29:**
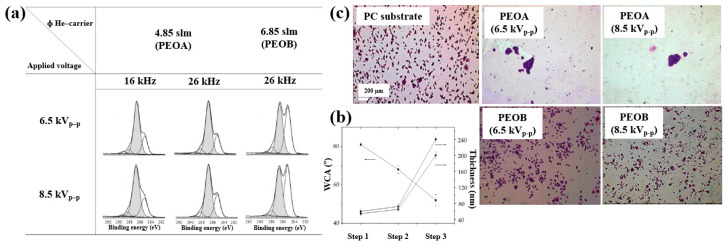
(**a**) XPS C 1s spectra of PEOA and PEOB films prepared by AP PE–CVD with three steps at 8.5 kV_p–p_ and frequencies of 16 and 26 kHz. (**b**) WCA values of PEOA films under same conditions. (**c**) Fluorescence images of osteoblasts attached to bare PC, PEOA, and PEOB films after 24 h of cultivation. Reproduced with copyright permission from Ref. [50].

XPS analysis (Figure 29a) confirmed that the chemical structure of the deposited polymer films varied with He–TEGDME gas flow compared to plasma voltage and frequency conditions. Figure 29b presents the thickness and WCA values of PEOA films under two frequency conditions. In step 1, a polyethylene (PE)–like hydrocarbon film with a thickness of 60 nm was deposited using He and C_2_H_4_ precursors, exhibiting hydrophobic properties with a WCA of 81°. In step 2, a 20 nm PE-like film was deposited using TEGDME, He, and C_2_H_4_ precursors, yielding a WCA of 68°. Finally, in step 3, a PEOA film with a thickness of 180 nm was deposited, exhibiting enhanced hydrophilicity with a WCA of 53°.

To evaluate cell adhesion on the deposited PEOA and PEOB films, biological experiments were conducted using various fibroblast cell types, including human dermal fibroblasts (HDFs), Saos–2 osteoblast cells, and normal HDFs (NHDFs). These cells were seeded onto the PEOA and PEOB films, and adhesion was assessed using fluorescence optical microscopy. After 24 h of cultivation (Figure 29c), a significant number of osteoblast cells were observed to adhere to the PEOB film surfaces prepared with a low PEOB content (<70%) at a He gas flow rate of 6.85 SLM, showing adhesion levels comparable to those observed on untreated PC substrates [50]. Table 17 summarizes the experimental synthesis conditions and corresponding biological results for Figure 28 and Figure 29 presented in this section.

**Figure 30:**
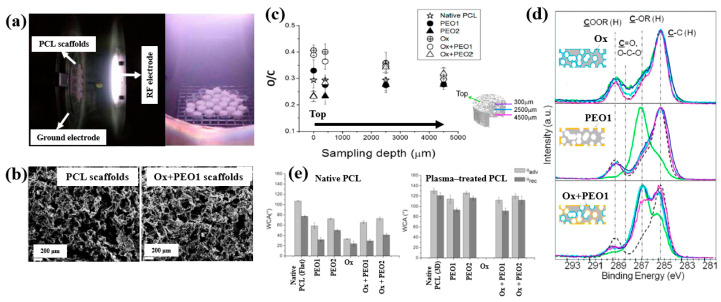
(**a**) Photographic image of plasma discharge during deposition in reactor. (**b**) FE–SEM images of PCL and Ox+PEO 1 scaffolds. (**c**) O/C ratio of native and plasma–treated PCL and PEO scaffolds. (**d**) XPS C 1s spectra at different depths for Ox, PEO1, and Ox+PEO1 scaffolds. (**e**) WCA results of native and plasma–treated PCL and PEO scaffolds. Reproduced with copyright permission from Ref. [51].

To enhance cell adhesion and proliferation properties, Sardella et al. [51] investigated poly(ethylene oxide) (PEO) coatings deposited on poly–ε caprolactone (PCL) scaffolds using a double-step cold plasma process with Ar and O_2_ gas mixtures (Figure 30a). The detailed experimental conditions for PEO deposition are summarized in Table 18 [51].

According to the FE–SEM results in Figure 30b, both the native PCL and Ox+PEO1 scaffolds exhibited interconnected pore structures with pore sizes ranging from 100 to 300 μm. Furthermore, as shown in Figure 30c, the O/C ratio at a depth of 2500 μm from the surface in plasma–treated scaffolds (PEO2 and PEO1) was similar to that of native PCL, indicating limited oxygen penetration into the scaffold core. However, the XPS results in Figure 30d demonstrate that PEO and Ox+PEO scaffolds possessed uniform oxygen-containing functional groups up to a depth of 3.5 mm along the vertical axis. Figure 30e presents the WCA measurements for native and plasma–treated PCL and PEO scaffolds. The plasma–treated samples exhibited enhanced hydrophilicity compared to the native PCL sample. Among these, PEO2 and Ox+PEO2 showed slightly greater hydrophobic behavior than PEO1 and Ox+PEO1. Notably, the Ox sample exhibited the highest degree of hydrophilicity among all samples tested [51].

**Figure 31:**
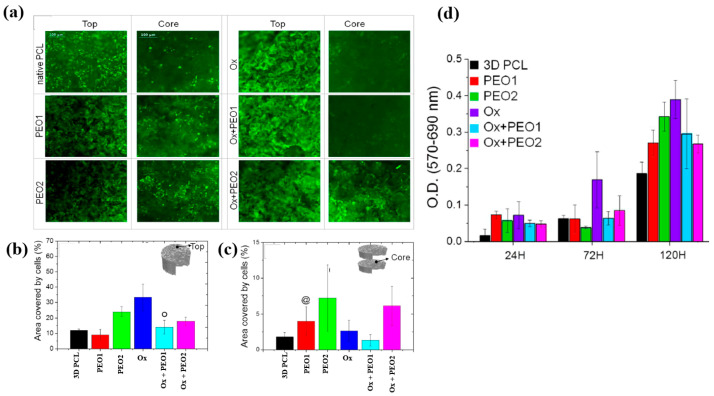
(**a**) Fluorescence images of osteoblasts attached to top and core regions of native and plasma–treated PCL scaffolds after 120 h of cultivation. Area coverage of cells on scaffold (**b**) top and (**c**) core regions. (**d**) MTT assay results for native and plasma–treated PCL and PEO scaffolds after 1, 3, and 5 d of cultivation. Reproduced with copyright permission from Ref. [51].

To examine cell adhesion and proliferation on native and plasma–treated PCL scaffolds, human osteoblast cells (Saos–2) were seeded onto the respective scaffolds and evaluated using fluorescence optical microscopy and the MTT colorimetric assay. As shown in Figure 31a, for all scaffolds, the number of cells attached to the scaffold core was lower than that on the top surface [51]. This is likely due to limited cellular diffusion into the interior of the scaffolds. The number of cells adhering to both the top and core regions of the scaffolds was quantified. According to Figure 31b,c, the Ox sample exhibited the highest cell density on the top surface. Additionally, after 120 h of cultivation, the PEO2 scaffold showed a high number of attached cells in both the top and core regions. Cell proliferation on the native and plasma–treated scaffolds was further evaluated using the MTT assay after 24, 72, and 120 h of cultivation. As shown in Figure 31d, no significant difference in cell proliferation was observed among the samples at 24 h. However, at 72 h, a significant increase in cell proliferation was observed only for the Ox sample. By 120 h, all plasma–treated scaffolds exhibited significantly enhanced cell proliferation compared to the native PCL [51]. This improvement in cell adhesion and proliferation is attributed to surface modifications induced by plasma treatment, including increased hydrophilicity and the incorporation of oxygen–containing polar functional groups. Table 19 presents a summary of the experimental synthesis and bacterial results for Figure 30 and Figure 31 in this section.

**Figure 32:**
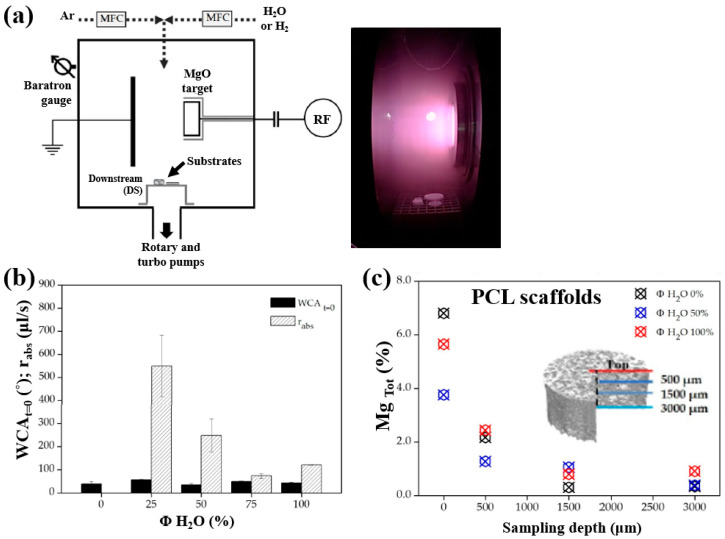
(**a**) Schematic of plasma reactor and photographic image of plasma discharge during deposition. (**b**) Water absorption rate and WCA values of plasma–treated PCL scaffolds with different gas mixtures (Ar, Ar/H_2_O, and H_2_O). (**c**) XPS surface composition of magnesium (Mg) (Mg _Tol_ %) in plasma–treated 3D PCL scaffolds at different surface depths. Reproduced with copyright permission from Ref. [52].

Armenise et al. [52] investigated the surface composition and wettability of poly–ε caprolactone (PCL) scaffolds containing magnesium (Mg), with a particular focus on their influence on cell adhesion. The Mg–containing coatings were prepared using a plasma-assisted technique via RF sputtering. Before MgO deposition, three-dimensional (3D) PCL scaffolds were fabricated using a solvent casting method with a PCL solution composed of chloroform (CHCl_3_) and NaCl as a porogen. Additionally, two–dimensional (2D) PCL flat samples were prepared by spin–coating using a mixed polymer solution, consisting of 1 g PCL dissolved in 6 mL CHCl_3_, onto polyethylene terephthalate (PET) substrates. Subsequently, the PCL samples were treated using an RF sputtering plasma–assisted technique, with a magnesium oxide (MgO) target and various injected gas mixtures. Figure 32a illustrates the experimental setup for MgO film deposition using RF sputtering. The plasma reactor was configured with two vertically aligned electrodes spaced 60 mm apart. The MgO target was placed on the powered electrode, while the opposing electrode was grounded. The PCL flat samples and scaffolds were positioned on a stainless steel grid located downstream (DS) from the plasma discharge region. Plasma treatment was conducted for 60 min at a power of 50 W. Figure 32b presents the WCA and water absorption rate of plasma–treated PCL scaffolds under Ar/H_2_O and pure H_2_O gas conditions. The results show that the water absorption rate decreased with increasing H_2_O concentration in the Ar/H_2_O mixtures. To assess elemental composition across the depth of the 3D–PCL scaffolds, XPS analysis was performed on cross–sections at varying distances from the surface. As shown in Figure 32c, the Mg concentration decreased from the top surface to a depth of 3000 μm, with the highest concentration observed near the surface at a depth of approximately 500 μm [52].

**Figure 33:**
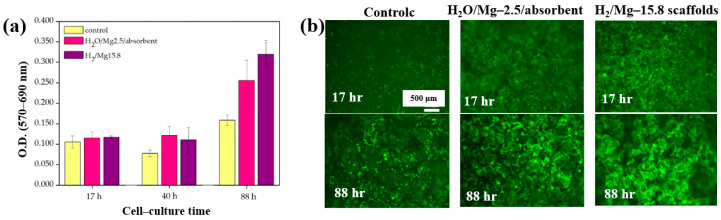
(**a**) Cell viability and (**b**) fluorescence microscopy images of Saos–2 cells on untreated (control) and plasma–treated 3D PCL scaffolds exposed to two gas mixtures (H_2_O/Mg–2.5/absorbent, H_2_/Mg–15.8) at cultivation times of 17, 40, and 88 h. Reproduced with copyright permission from Ref. [52].

Furthermore, cell adhesion and viability of Saos–2 osteoblast cells cultured on three different PCL scaffolds were assessed at various cultivation times (17, 40, and 88 h) by using fluorescence microscopy. As shown in Figure 33a, no significant differences in cell adhesion or growth were observed after 17 and 40 h of cultivation. However, after 88 h, cell proliferation was evident across all scaffolds. In particular, enhanced adhesion and proliferation were observed on the two 3D–PCL scaffolds, such as on H_2_O/Mg 2.5/absorbent and H_2_/Mg 15.8 scaffolds. Fluorescence microscopy images (Figure 33b) further illustrate the adhesion and proliferation behavior of Saos–2 cells after 17 and 88 h of cultivation on these two scaffold types [52]. Table 20 summarizes the experimental synthesis and bacterial assay results corresponding to Figure 32 and Figure 33.

**Figure 34:**
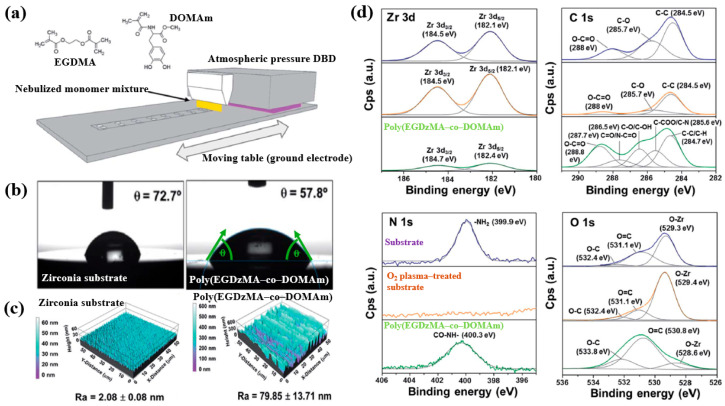
(**a**) Schematic of liquid-assisted APP-induced polymerization (LA–APPiP) for poly(EGDMA–co–DOMAm) copolymer deposition. (**b**) WCA results, (**c**) AFM images, and roughness (R_a_) of zirconia substrate and poly(EGDMA–co–DOMAm) films. (**d**) XPS high–resolution spectra of untreated zirconia and O_2_ plasma–treated zirconia and poly(EGDMA–co–DOMAm) copolymer. Reproduced with copyright permission from Ref. [53].

Hod’asov’a et al. [53] investigated the deposition of a poly(EGDMA–co–DOMAm) copolymer film on a zirconia substrate via liquid–assisted APP-induced polymerization (LA–APPiP) using two monomer mixtures of methyl–DOPA methacrylamide (DOMAm), and ethylene glycol dimethacrylate (EGDMA) [53]. The experimental setup for copolymer film deposition is shown in Figure 34a. The film was fabricated in two steps. First, the mixed liquid solutions were sprayed to form a thin liquid layer on the substrate. Then, the layer was polymerized using plasma generated by a sinusoidal waveform at 10 kHz, with an Ar gas flow of 20 SLM. Deposition was carried out for 8 s, resulting in a copolymer film approximately 250 nm thick. As shown in Figure 34b, the WCA value for the zirconia substrate and the copolymer film was 72.7 and 57.8, respectively. Figure 34c presents the AFM images and surface roughness (R_a_) values. The untreated zirconia substrate exhibited a low R_a_ of 2.1 nm, indicating a flat surface. In contrast, after deposition by LA–APPiP, the R_a_ of the copolymer film increased substantially to 79.9 nm. Figure 34d shows the XPS high-resolution spectra for three samples, namely untreated zirconia, O_2_ plasma–treated zirconia, and the deposited copolymer film. No significant difference was observed between the untreated and O_2_ plasma–treated zirconia surfaces. However, new functional groups such as C=O/N–C=O, C–O/C–OH, and C–COO/C–N were detected in the deposited copolymer film [53].

**Figure 35:**
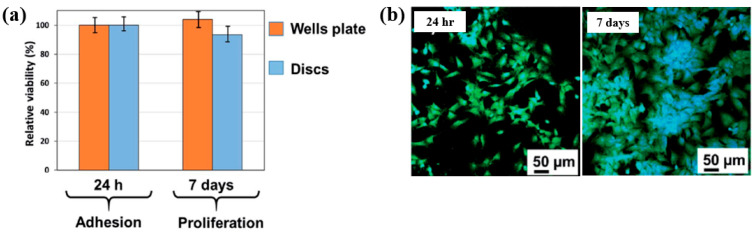
(**a**) Cell viability of MG–63 cells after 24 h and cell proliferation after 7 d. (**b**) Fluorescence microscopy images of osteogenic MG–63 cells attached to poly(EGDMA–co–DOMAm) films after 24 h and 7 d of cultivation. Reproduced with copyright permission from Ref. [53].

To examine cell adhesion on the poly(EGDMA–co–DOMAm) film, osteoblast-like MG–63 cells were seeded onto the deposited films and analyzed using fluorescence optical microscopy. As shown in Figure 35a,b, cell attachment and proliferation were enhanced on the poly(EGDMA–co–DOMAm) film after 24 h and 7 d of cultivation, respectively [53]. This enhancement is attributed to the presence of functional groups and the increased surface roughness of the plasma-deposited poly(EGDMA–co–DOMAm) copolymer film. Table 21 presents a summary of the experimental synthesis and bacterial assay results corresponding to Figure 34 and Figure 35 in this section.

Solovieva et al. [54] investigated the viability and proliferation of mesenchymal stem cells (MSCs) on plasma–deposited polymer films coated on PCL nanofibers (NFs). First, the nanofibers were fabricated by electrospinning using a PCL solution, and the resulting fibers were designated as PCL–ref. A polymer film containing COOH functional groups was then deposited onto the PCL NFs via capacitively coupled RF plasma under vacuum using a precursor gas mixture of Ar, CO_2_, and C_2_H_4_. These plasma–coated nanofibers were designated as PCL–COOH. To evaluate different modes of platelet–rich plasma (PRP) adsorption and bonding on the PCL NFs, three additional treatments were performed. For the physical adsorption of PRP, PCL–ref samples were immersed in PRP solution at RT for 15 min and then rinsed with phosphate-buffered saline (PBS). These samples were designated as PCL–P1. For ionic bonding, PCL–COOH samples were dipped in a PRP solution for 15 min and washed with PBS solution, and designated as PCL–COOH–P2. Finally, for covalent bonding, PCL–COOH samples were treated with N–dicyclohexylcarbodiimide (DCC) solution (2 mg/mL in water) for 15 min, followed by PBS washing. These were designated as PCL–COOH–P3. The detailed identification of all five sample types is provided in Table 22.

**Figure 36:**
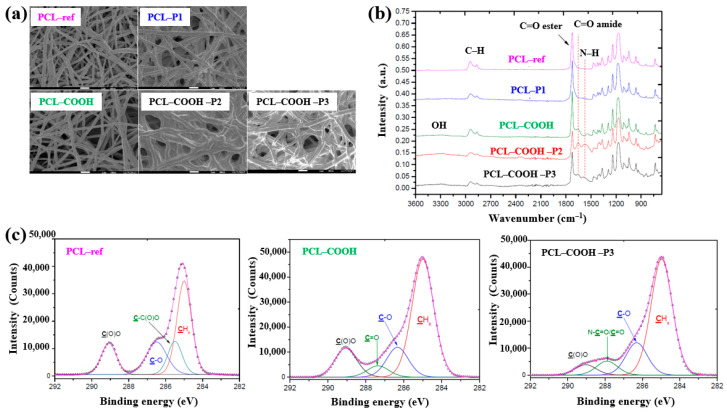
(**a**) FE–SEM images and (**b**) FT–IR spectra of polymer samples, namely polycaprolactone nanofibers (PCL–ref), PCL–P1, PCL–COOH, PCL–COOH–P2, and PCL–COOH–P3. (**c**) XPS C 1s high-resolution spectra of PCL–ref, PCL–COOH, and PCL–COOH–P3. Reproduced with copyright permission from Ref. [54].

As shown in the FE–SEM analysis in Figure 36a, the morphology of the PCL–P1 sample was similar to that of PCL–ref, both before and after plasma polymer coating. Additionally, the diameters of all electrospun PCL NF samples were consistently found to be approximately 270 nm. The FT–IR spectra in Figure 36b reveal new peaks at 1652 and 1598 cm^−1^, corresponding to C=O stretching and the NH_2_ bond of amide groups, respectively, on the surface of the plasma polymer-coated PCL NFs. These peaks are attributed to the introduction of COOH functional groups from the plasma polymer layer and the presence of molecules containing peptide bonds. Furthermore, XPS analysis (Figure 36c) shows that PCL–COOH consisted of 72.1 at% carbon and 27.9 at% oxygen. After immobilization with PRP via ionic bonding, PCL–COOH–P2 exhibited a composition of 70.5 at% carbon, 18.8 at% oxygen, and 10.7 at% N_2_. Similarly, PCL–COOH–P3, treated for covalent immobilization of PRP, showed a comparable elemental composition. Notably, in the C1s spectrum of PCL–COOH–P3 NFs, a shift in the carbon peak was observed after PRP immobilization. The intensity of the C(O)O peak decreased, while a new peak corresponding to amide groups (N–C=O) emerged [54].

**Figure 37:**
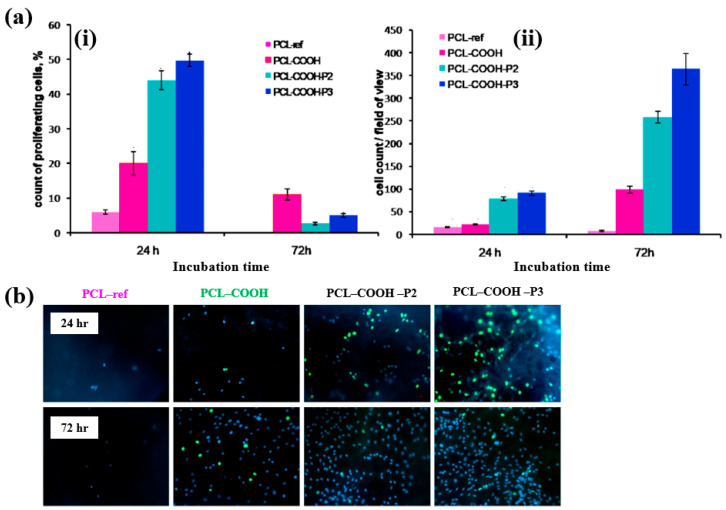
(**a**(**i**)) Count percentage and (**a**(**ii**)) average number of proliferating cells after 24 and 72 h of cultivation with different plasma polymer-coated PCL nanofibers (NFs). (**b**) Fluorescence images of human MSCs on plasma polymer–coated PCL NFs after 24 and 72 h of cultivation. Reproduced with copyright permission from Ref. [54].

To evaluate cell adhesion and proliferation of human bone marrow (BM) mesenchymal stromal cells (MSCs) on various plasma polymer-coated PCL NFs, human MSCs were seeded onto the coated NFs and analyzed using fluorescence optical microscopy. As shown in Figure 37a,b, the PCL–COOH sample exhibited limited cell adhesion, with cells displaying a smaller spreading area compared to the PCL–ref sample. In contrast, the PCL–COOH–P2 and PCL–COOH–P3 samples showed uniformly attached cells with high density. Furthermore, after 72 h of cultivation, the attached cells remained viable, and cell proliferation was notably enhanced [54]. Table 23 presents a summary of the experimental synthesis and bacterial assay results corresponding to Figure 36 and Figure 37.

Furthermore, additional information related to plasma synthesis and biomedical experiments from published papers without copyright permission approval is summarized and presented in Table 24 [55,56,57,58].

Table 25 summarizes the plasma-based synthesis of polymer films for biomedical applications discussed in this section.

### 2.2. Plasma Surface Treatment of Polymer Films for Biomedical Applications

**Figure 38:**
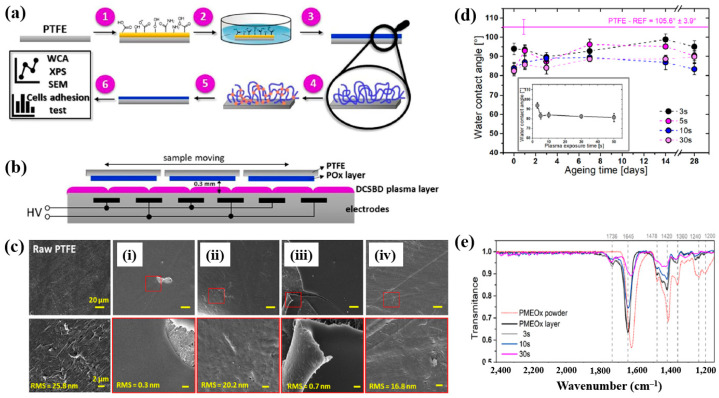
(**a**) Experimental setup for PMEOx film plasma deposition and PTFE surface treatment. (**b**) Side view of DCSBD plasma device. (**c**) SEM images of (i) raw PTFE, (ii) PMEOx deposited on pre–treated PTFE, (iii) PMEOx on pre–treated PTFE post washing, and (iv) air plasma–treated PMEOx post washing. (**d**) WCA results of air plasma–treated PTFE after 28 d of storage. (**e**) FT–IR spectra of PMEOx powder, untreated PMEOx layer, and plasma–treated PMEOx layer after 3, 10, and 30 s exposure without washing. Reproduced with copyright permission from Ref. [59].

To deposit the PMEOx layer, the polytetrafluoroethylene (PTFE) surface must first be modified with reactive functional groups. Figure 38a,b illustrate the experimental procedures for the plasma deposition of poly(2–oxazolines) (POx) films and the surface treatment of PTFE using a diffuse coplanar surface barrier discharge (DCSBD) device based on DBD technology [59]. Both the pre–treatment of PTFE and the post–treatment of the deposited poly(2–methyl–2–oxazoline)–stat–(2–(3–butenyl)–2–oxazoline) (PMEOx) layer were carried out using the DCSBD system. The DCSBD apparatus comprises 16 pairs of parallel-aligned Ag electrodes embedded in 0.6 mm thick alumina ceramics, operating at a peak–to–peak voltage (V_p-p_) of 20 kV and a frequency of 15 kHz. This configuration enables the generation of a uniform plasma across a flat dielectric ceramic surface between the electrodes. Initially, the PTFE substrate was treated with ambient air plasma using a sinusoidal voltage for varying durations (3, 5, 10, 30, and 50 s). Following plasma activation, the samples were immersed in a mixed solution of chloroform and 5 wt% PMEOx for 1 min via dip–coating. After removal from the solution, the samples were dried for 24 h under ambient conditions. Subsequently, the PMEOx–coated PTFE underwent a 3 s post-plasma treatment in ambient air at 15 kHz. During this process, the sample was placed in a holder mounted on a moving stage within the DCSBD chamber. Before plasma activation, the chamber was pumped and purged with Ar gas at a flow rate of 1.0 L·min^−1^ for 5 min [59]. The surface morphology and roughness were analyzed using atomic force microscopy (AFM) and scanning electron microscopy (SEM). The untreated PTFE exhibited a rough surface with a roughness of 25.8 nm (Figure 38c) [59]. In contrast, the PMEOx-deposited surface was notably smoother, with a roughness of 0.3 nm (Figure 38c(i)). After air plasma treatment, the surface roughness slightly increased to 0.7 nm, indicating minor surface modification compared to the untreated PMEOx layer (Figure 38c(iii)). Evidence of plasma–induced surface damage was observed on the air plasma–treated PMEOx layer (Figure 38c(i,iii). Despite plasma exposure and subsequent washing, both pristine and air plasma–treated PMEOx layers remained well adhered to the PTFE substrate, as shown in Figure 38c(ii,iv)) [59]. The raw PTFE exhibited high hydrophobicity due to the strong electronegativity of fluorine atoms, with a WCA of 105.6°. To facilitate PMEOx deposition, plasma treatment was applied to introduce reactive functional groups onto the PTFE surface. After 3 s of air plasma treatment, the WCA decreased from 105.6° to 93.9° (Figure 38d). With extended plasma exposure beyond 50 s, the WCA further decreased to 81.5°, the lowest observed value (Figure 38c). This reduction in WCA is attributed to the DCSBD system, which promotes surface functionalization by generating reactive species that disrupt chemical bonds, thereby forming new functional groups on the PTFE surface. The chemical structures of both the raw PMEOx powder and the air plasma-treated PMEOx film were analyzed using FT–IR spectroscopy. As shown in Figure 38e, the raw PMEOx powder displays a broad peak at 1624 cm^−1^ corresponding to amide functional groups and a peak at 1478 cm^−1^ associated with CH_2_ deformations within the polymer backbone. Additional features include a broad shoulder at 1450 cm^−1^ and a peak at 1360 cm^−1^, corresponding to asymmetric and symmetric CH_3_ deformations, respectively, along with a broad peak in the 1240–1200 cm^−1^ range attributed to C–N stretching vibrations. In the deposited PMEOx film, the amide peak shifts to 1645 cm^−1^, and a new peak at 1736 cm^−1^ appears, corresponding to C=O bonds. The POx film also exhibits new functional groups, such as isocyanates (2170 cm^−1^) and nitriles (2250 cm^−1^). As plasma treatment time increases, the intensity of these FT–IR peaks decreases, indicating that the upper layer of the PMEOx film undergoes etching during plasma exposure [59]. Consequently, plasma treatment not only modifies the chemical structure of the PMEOx film but also enables the formation of new functional groups on the underlying PTFE surface, enhancing its chemical reactivity and suitability for subsequent coating applications.

**Figure 39:**
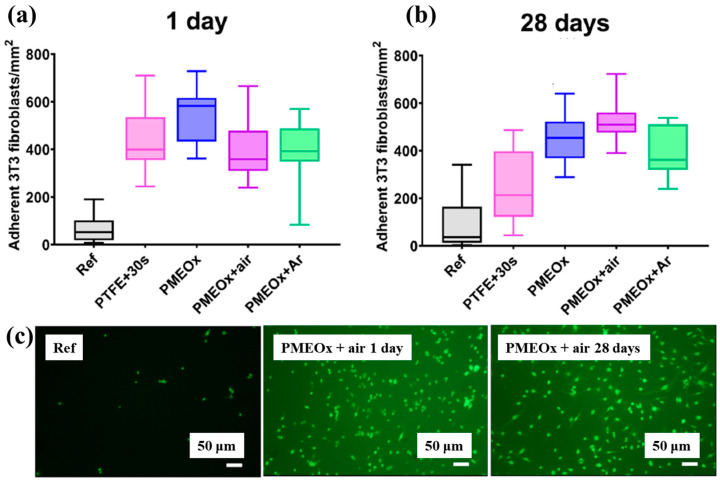
Adhesion of fibroblasts (3T3) on PTFE and POx surfaces after 1 d of incubation. Density of adherent cells (**a**) 1 d post preparation and (**b**) after 30 d of aging. (**c**) Fluorescence images of adherent cells stained with FDA (scale bar: 50 μm). Reproduced with copyright permission from Ref. [59].

To assess biomaterial properties, this study examined the adhesion of mouse fibroblast cells (3T3). As shown in Figure 39a, the number of adherent fibroblasts after 1 d was relatively low on the untreated PTFE surface, with a median of 69 cells mm^−2^ [59]. In contrast, the air plasma–treated PTFE surface exhibited significantly enhanced adhesion, reaching 434 cells·mm^−2^, attributed to the introduction of polar functional groups via plasma treatment. After 28 d of storage, fibroblast adhesion further increased across all POx–coated samples, as illustrated in Figure 39b. To validate these adhesion properties, fluorescence imaging of adherent cells was conducted. Figure 39c confirms that, even after 28 d of storage, the cell–adhesive properties remained stable, owing to the durable formation of the POx layer on the air plasma-treated PTFE surface [59]. Table 26 summarizes the experimental surface treatments and bacterial results presented in Figure 38 and Figure 39.

**Figure 40:**
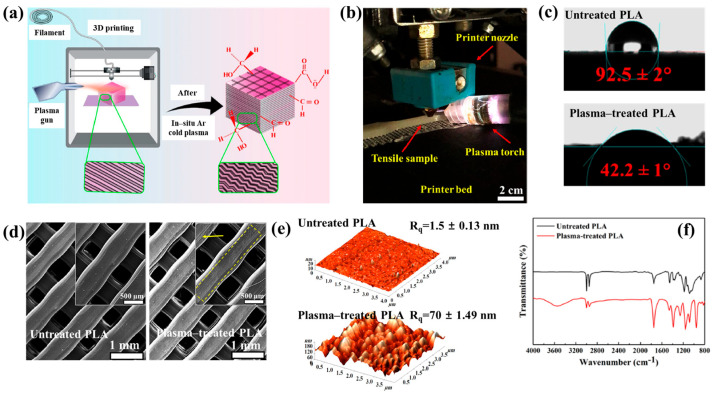
(**a**) Schematic of 3D–printed PLA scaffold treatment with in situ Ar APP system, adapted from Zarei et al. (**b**) Photographic image of plasma–treated scaffold. (**c**) WCA, (**d**) FE–SEM images, (**e**) AFM images, and (**f**) FT–IR spectra of untreated and plasma–treated PLA scaffolds. Reproduced with copyright permission from Ref. [60].

Figure 40a,b present a schematic diagram and photograph of 3D-printed polylactic acid (PLA) treated using an in situ Ar APP system developed by Zarei et al. [60].

In this setup, PLA samples were treated with Ar plasma simultaneously during the 3D printing process. The plasma source consists of a torch–type electrode configuration comprising Cu foil wrapped around the outside of a quartz tube and a 0.5 mm diameter Cu wire positioned inside. The torch was fabricated using a quartz tube with an inner diameter of 2.5 mm and a wall thickness of 1 mm, with the dielectric quartz insulating the electrodes. Upon application of a high–voltage power supply and Ar gas injection, a DBD plasma was generated between the electrodes. Plasma discharge was maintained by injecting Ar gas at a flow rate of 1.5 L/min through a 0.7 mm diameter nozzle. Operating conditions included a voltage of 15 kV and a current of 3.4 mA. During operation, Ar gas flow was regulated to sustain a plasma plume length of approximately 12–15 mm. The nozzle–to–nozzle distance between the plasma torch and the 3D printer was maintained at 10 mm, enabling effective in situ plasma treatment of the PLA surface during printing [60]. As shown in Figure 40c, the untreated PLA scaffold is hydrophobic, exhibiting a WCA of 92.5°. Following Ar plasma treatment, the WCA decreased by 42.2°, indicating a transition to hydrophilic behavior. For enhanced cell attachment, scaffolds must exhibit suitable surface roughness and an open, interconnected structure. Figure 40d displays FE–SEM images of untreated and plasma–treated PLA surfaces. The untreated 3D-printed PLA scaffold exhibits a generally smooth surface with pores approximately 500 μm in diameter.

In contrast, Ar plasma treatment induces increased surface roughness due to the formation of a nano–patterned surface morphology. Quantitative analysis of surface roughness was performed using 2D and 3D AFM images measured over a 4.0 μm × 4.0 μm scan area, as shown in Figure 40e. The untreated scaffold presents a smooth surface with a root mean square roughness (R_q_) of 1.5 nm, whereas the plasma–treated scaffold develops a groove–like structure with a significantly increased R_q_ of 70 nm [60]. To further investigate hydrophilic modifications, FT–IR spectroscopy was conducted. For the untreated PLA scaffold, the peak at 1080 cm^−1^ corresponds to –O–C=O bonding, while the peak at 1175 cm^−1^ is attributed to –CH–O stretching. Additionally, the peak at 1265 cm^−1^ is assigned to C–O bonding within the ester group of PLA, and the peak at 1745 cm^−1^ is associated with C=O bonding, as shown in Figure 40f. After Ar plasma treatment, the intensities of these oxygen–containing functional groups, including –O–C=O, –CH–O, C=O, and C–O, were enhanced, indicating surface structural modification. This enhancement results from reactive plasma species interacting with the PLA surface, disrupting the polymer matrix and subsequently forming new oxygen–containing compounds through reactions with ambient air. These newly introduced functional groups increase the hydrophilicity of the plasma-treated PLA surface [60].

**Figure 41:**
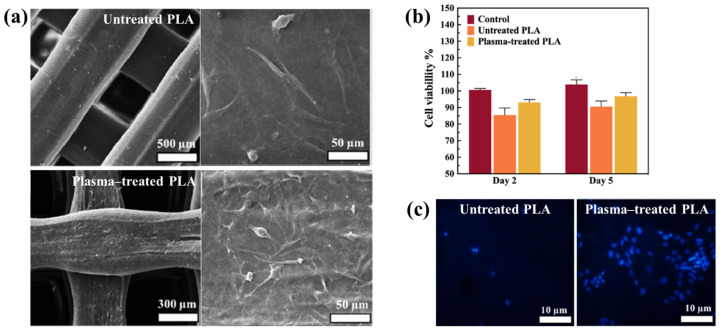
(**a**) FE–SEM images of untreated and plasma-treated 3D-printed PLA scaffolds after 5 d cultivation. (**b**) Cell viability analysis of untreated and plasma–treated scaffolds after 5 d of cultivation. (**c**) Fluorescence images of DAPI-stained cells on untreated and plasma–treated scaffolds assessed with MTT assay. Reproduced with copyright permission from Ref. [60].

To verify the effect of in situ plasma treatment on 3D–printed PLA scaffolds in promoting the adhesion of cultivated human adipose–derived stem cells (hADSCs), FE–SEM analysis was performed. As shown in Figure 41a, hADSCs on untreated PLA scaffolds exhibit smooth morphology with spindle–like patterns, whereas cells on plasma–treated PLA scaffolds display a more irregular distribution and predominantly polygonal shapes [60]. After a 5 d cultivation period, the number of hADSCs on plasma–treated scaffolds was higher than that on untreated scaffolds. Cell viability was assessed using an MTT assay after 2 and 5 d of cultivation. As illustrated in Figure 41b, viability was significantly greater on the plasma–treated PLA scaffolds compared to the untreated ones. To evaluate cell adhesion, DAPI staining was employed as a fluorescent marker. Following cell seeding on the PLA scaffolds, DAPI staining enabled visualization of cell morphology, as shown in Figure 41c. The results confirm enhanced adhesion of hADSCs on the plasma–treated PLA surface [60]. Table 27 summarizes the experimental surface treatment and bacterial results corresponding to Figure 40 and Figure 41.

**Figure 42:**
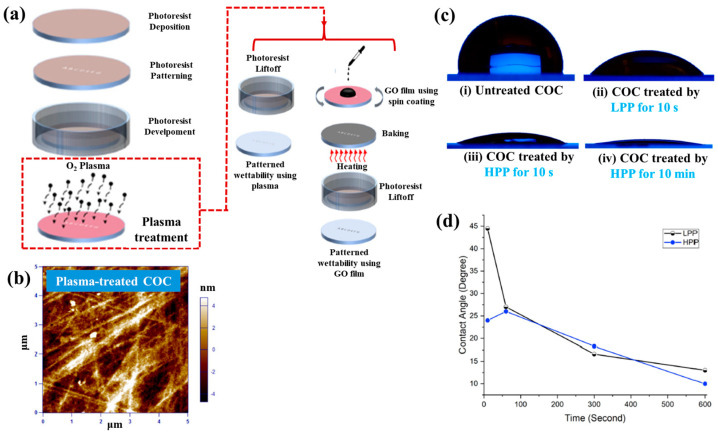
(**a**) Schematic of plasma-based patterning procedure on cyclic olefin copolymer (COC) surface. (**b**) AFM images of plasma–treated COC surface. (**c**) Photographic images of water droplets on (i) untreated COC, (ii) COC treated with LPP for 10 s, (iii) COC treated with HPP for 10 s, and (iv) COC treated with HPP for 10 min. (**d**) WCA measurements for COC treated under different LPP and HPP conditions. Reproduced with copyright permission from Ref. [61].

Figure 42a presents the experimental procedure for patterning the cyclic olefin copolymer (COC) substrate with a graphene oxide (GO) film and applying plasma surface treatment to enhance surface wettability [61]. The COC substrate was patterned using commercial photolithography and O_2_ plasma treatment. The substrate was initially cleaned via sonication in isopropanol and deionized water for 5 min, dried with N_2_ gas, and heated to 70 °C for 10 min. Photoresist (PR) was then deposited using a spin–coater, followed by heating and photolithographic patterning. Plasma treatment was applied using a PDC–002 system (Harrick Plasma, USA) for 5 min at 700 mTorr under two power conditions, namely low–power plasma (LPP, 7.2 W) and high–power plasma (HPP, 29.6 W). Finally, the PR layer was removed by ultrasonic washing in acetone [61]. The surface roughness of plasma-treated and GO–coated COC substrates was analyzed using AFM over a 5 μm × 5 μm scan area at 1 Hz. As shown in Figure 42b, the roughness values were 4.3 nm for plasma-treated COC and 2.5 nm for GO-coated COC [61]. Figure 42c displays images of water droplets on various plasma–treated COC samples, namely (i) untreated COC, (ii) COC treated with LPP for 10 s, (iii) COC treated with HPP for 10 s, and (iv) COC treated with HPP for 10 min. The untreated surface (Figure 42c(i)) exhibited a WCA of 110°, whereas plasma–treated surfaces showed reduced WCAs of 45°, 24°, and 10°, as shown in Figure 42c(ii), Figure 42c(iii), and Figure 42c(iv), respectively. To further evaluate the influence of plasma power and exposure time on wettability, Figure 42d presents WCA measurements for LPP and HPP treatments. HPP treatment reduced the WCA from 110° to 24° after 10 s and to 10° after 10 min. Similarly, LPP treatment decreased the WCA to 45° after 10 s and to 13° after 10 min [61].

**Figure 43:**
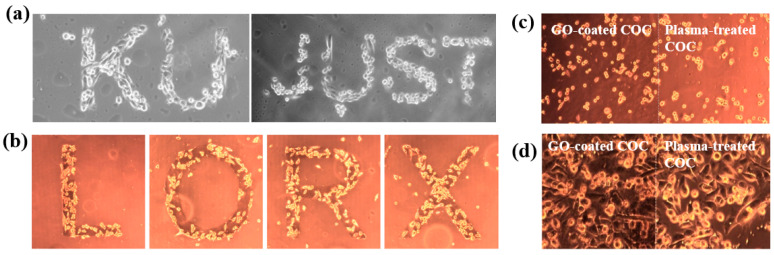
(**a**) Microscopic images of breast cancer cells (MDA–MB–231) seeded in patterned shapes of “KU” and “JUST” on plasma–treated COC substrates. (**b**) Cancer cells patterned into different alphabet letters. Microscopic images of cells on GO-coated and plasma–treated COC after (**c**) 12 h and (**d**) 5 d. Reproduced with copyright permission from Ref. [61].

Figure 43a presents microscopic images of breast cancer cells (MDA–MB–231) seeded onto patterned KU and JUST shapes on COC substrates [61]. Due to the inherent hydrophobicity of the original COC surface, the alphabet-shaped regions were treated with O_2_ plasma to enhance hydrophilicity and thereby improve cell adhesion. As shown in Figure 43b, cells adhered and proliferated effectively on the GO-coated and plasma–treated COC surfaces with patterned lettering. To compare cell adhesion between plasma-treated and GO-coated COC surfaces, MDA–MB–231 cells were cultured on both. As illustrated in Figure 43c, after 5 d of incubation, cells on the GO-coated surface appeared elongated prior to attachment to the plasma-treated COC. Figure 43d shows that cell proliferation occurred effectively on both types of substrates [61]. Table 28 summarizes the experimental surface treatments and bacterial results corresponding to Figure 42 and Figure 43.

**Figure 44:**
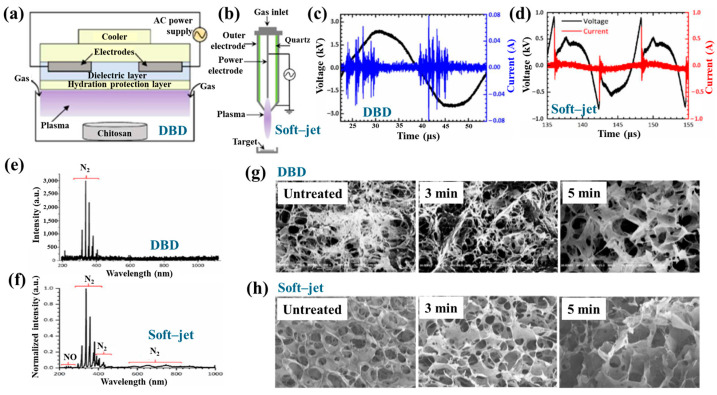
Schematic of (**a**) DBD device and (**b**) soft–jet plasma device. Current–voltage waveforms during plasma discharge of (**c**) DBD and (**d**) soft–jet plasma. OES spectra of (**e**) DBD and (**f**) soft–jet plasma. SEM images of CS scaffold after (**g**) DBD and (**h**) soft–jet plasma treatment. Reproduced with copyright permission from Ref. [62].

**Figure 45:**
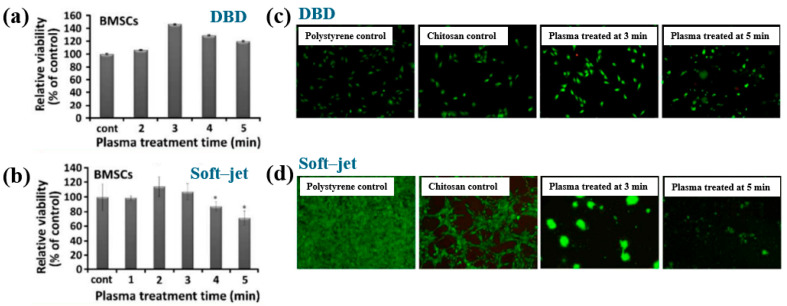
(**a**) Viability of BMSCs on scaffolds treated with DBD plasma. (**b**) Viability of BMSCs on scaffolds treated with soft–jet plasma. (**c**) Fluorescence images of cells cultured on untreated polystyrene (control), untreated CS scaffold (control), and DBD–treated scaffolds (3 and 5 min). (**d**) Fluorescence images of cells on scaffolds treated with soft–jet plasma. Reproduced with copyright permission from Ref. [62].

Han et al. investigated the effects of plasma treatment on 3D porous CS scaffolds using DBD and soft–jet plasma systems, focusing on cell adhesion and the growth performance of bone marrow (BM) –derived stem cells (BMSCs), as shown in Figure 44 and Figure 45 [62]. Plasma treatment was conducted using two distinct sources, including a DBD and soft jet plasma source. Figure 44a,b present schematic diagrams of the DBD and soft–jet plasma devices, respectively. In the DBD setup, an AC sinusoidal peak voltage of 2 kV was applied to the powered electrode, with N_2_ gas supplied at a flow rate of 1.5 L per minute (LPM) to generate plasma. Figure 44c illustrates the voltage–current waveform signals of the DBD device during plasma discharge. CS scaffolds were treated at a 2 cm distance for varying durations of 1, 2, 3, 4, and 5 min. Similarly, the soft jet plasma device, shown in Figure 44d, used N_2_ gas with an applied AC peak voltage of 500 V and a current of 13 mA. CS scaffolds were treated using the soft jet plasma for the same durations (1, 5 min) [62]. Figure 44e,f display the optical emission spectroscopy (OES) spectra of the plasma generated by the DBD and soft jet devices. Notably, the OES spectrum of the soft jet plasma showed the presence of N_2_ and hydroxyl (OH) radicals, along with higher peak intensities of nitric oxide (NO) and nitrogen dioxide (NO_2_) compared to the DBD plasma. The untreated CS scaffolds exhibited a highly porous, 3D sponge–like microstructure, as shown in Figure 44g,h. Figure 44g presents the SEM images of untreated scaffolds and those treated with DBD plasma for 3 and 5 min, while Figure 44h displays SEM images of scaffolds treated with soft jet plasma for the same durations.

After 5 min of DBD plasma treatment, the CS scaffolds demonstrated a more uniform pore distribution and increased inter–pore spacing (Figure 44g) compared to those treated with the soft jet plasma (Figure 44h). Additionally, surface roughness increased following 5 min of DBD plasma exposure [62]. These changes in surface composition and topography are attributed to cell adhesion.

To verify the effect of plasma treatment on cell viability, BMSCs cultured on CS scaffolds were evaluated under various DBD and soft–jet plasma treatment conditions. As shown in Figure 45a, BMSC viability increased on scaffolds treated with DBD plasma [62]. A similar trend was observed for scaffolds treated with soft–jet plasma as demonstrated in Figure 45b, where cell viability also improved following treatment. Figure 45c presents fluorescence images of live/dead stained cells cultured on untreated polystyrene, untreated CS scaffolds, and CS scaffolds with DBD plasma for 3 and 5 min. In both the untreated control and DBD–treated groups, green fluorescence indicates the presence of live cells. The proliferation of BMSCs was notably enhanced on DBD–treated scaffolds, as evidenced in Figure 45c. In contrast, as shown in Figure 45d, scaffolds treated with soft–jet plasma for 3 and 5 min exhibited no significant improvement in cell attachment compared to those treated with DBD. These findings confirm that DBD–treated CS scaffolds promote higher cell attachment relative to soft jet–treated scaffolds [62]. The enhanced cell attachment is attributed to modifications in CS surface composition, specifically the introduction of amino and OH functional groups, as well as increased surface roughness induced by RONS present in the N_2_ plasma. In particular, the amino groups, being positively charged, may electrostatically interact with negatively charged cell membranes, thereby facilitating improved attachment. Table 29 summarizes the experimental synthesis procedures and biological results corresponding to Figure 44 and Figure 45.

Sabrin et al. [63] investigated a plasma–activated hydrogel therapy (PAHT) process for fabricating dressings with high concentrations of H_2_O_2_ and antibacterial activity.

**Figure 46:**
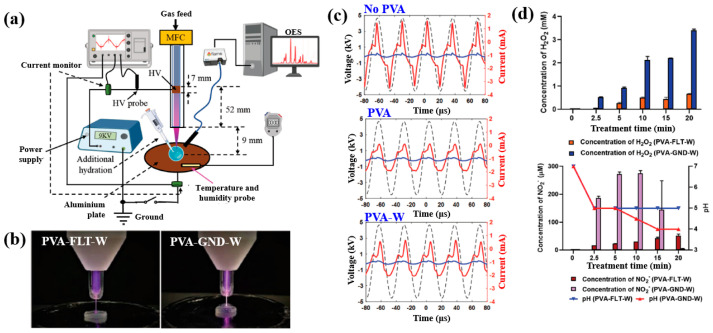
(**a**) Experimental setup for poly(vinyl alcohol) (PVA) surface treatment using a He plasma jet and photograph of plasma jet during treatment. (**b**) Photographic images of He plasma jet under PVA–FLT–W and PVA–GND–W conditions. (**c**) Voltage and current profiles of He plasma jet during treatment of different PVA samples (no PVA, PVA, and PVA–W) under grounded and floating conditions. (**d**) Concentrations of H_2_O_2_ and NO_2_ formed in PVA–FLT–W and PVA–GND–W hydrogels with increasing treatment time. Reproduced with copyright permission from Ref. [63].

A 1 mm thick poly(vinyl alcohol) (PVA) hydrogel was used for plasma surface treatment. Figure 46a shows the experimental setup for PVA hydrogel treatment along with photographic images of the He plasma jet during operation. The PVA hydrogel was placed on an aluminum (Al) plate and treated using a He plasma jet operating at 9 kV_p–p_ and 30 kHz under two electrode configurations, namely grounded and floating. As shown in the images in Figure 46b, plasma discharge appeared stronger when the Al plate was grounded (PVA–GND–W) compared to the floating condition (PVA–FLT–W). This enhancement is attributed to the increased density of high-energy species in the plasma jet, which facilitates RONS generation. Figure 46c presents the voltage and current waveforms of the He plasma jet during treatment of different PVA configurations (no PVA, PVA, and PVA–W) under floated and grounded conditions. Compared to the floated PVA hydrogel, the grounded PVA hydrogel exhibited a significantly higher discharge current, likely due to the greater accumulation of surface charge and the formation of a direct electron flow channel. This resulted in more intense plasma discharge under the grounded condition. To assess the formation of H_2_O_2_ and NO_2_^−^ in the PVA hydrogel, solution pH was monitored as a function of plasma treatment time. As shown in Figure 46d, the concentrations of H_2_O_2_ and NO_2_^−^ in the PVA–GND–W hydrogels increased with longer plasma exposure times [63].

**Figure 47:**
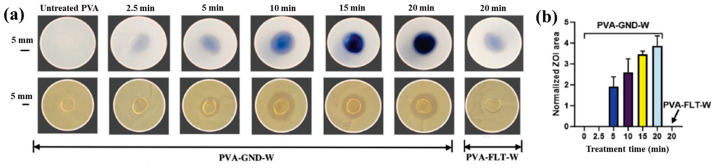
(**a**) Photographs of zone of inhibition (ZOI) demonstrating antibacterial properties. (**b**) Comparison of normalized ZOI areas for PVA–GND–W and PVA–FLT–W hydrogel films with increasing plasma treatment time. Reproduced with copyright permission from Ref. [63].

The antibacterial properties of untreated and plasma-treated PVA hydrogels were evaluated using oxidation measurements via an agarose–KI–starch film assay. PVA samples were placed at the center of bacterial cultures and incubated at 35 °C for 24 h. As shown in Figure 47a, the color intensity of the zone of inhibition (ZOI) area in the agarose film became a progressively darker purple with increasing plasma treatment time, indicating elevated levels of KI oxidation. In contrast, the PVA–FLT–W hydrogel film treated for 20 min exhibited a lighter purple ZOI compared to the PVA–GND–W hydrogel film treated under the same conditions. The quantified ZOI measurements shown in Figure 47b demonstrate that ZOI length increased with longer plasma treatment durations, suggesting enhanced antibacterial activity in the PVA–GND–W hydrogel films [63]. This improvement is likely due to the increased formation of H_2_O_2_ and NO_2_^−^ within the plasma-treated PVA hydrogel, generated by He plasma jet exposure. Table 30 summarizes the plasma surface treatment conditions and corresponding bacterial assay results related to Figure 46 and Figure 47.

**Figure 48:**
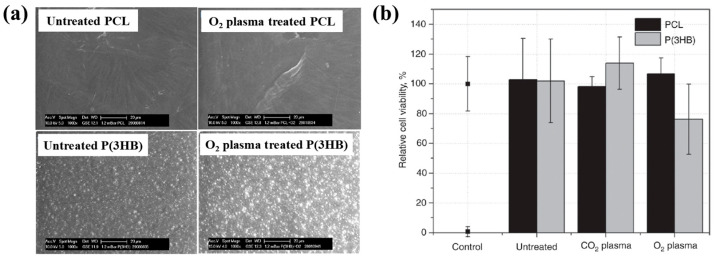
(**a**) FE-SEM images of poly(ε-caprolactone) (PCL) and poly(3–hydroxybutyrate) [P(3HB)] films before and after O_2_ plasma treatment. (**b**) Cell viability of mouse fibroblast cells (L929) after 48 h of incubation on untreated and plasma–treated PCL and P(3HB) films using CO_2_ and O_2_ plasma. Reproduced with copyright permission from Ref. [64].

Surface treatment of poly(ε–caprolactone) (PCL) and poly(3–hydroxybutyrate) [P(3HB)] was investigated by Teske et al. with the aim of enhancing the biocompatibility of scaffold material for bioartificial vessel prosthesis applications [64]. Surface modification of PCL and P(3HB) polymers was performed using CO_2_ and O_2_ plasma to introduce functional groups onto polymer surfaces. As shown in Figure 48a, there were no significant changes in surface morphology for either PCL or P(3HB) before and after O_2_ plasma treatment.

To assess cell viability, mouse fibroblast cells (L929) were cultured on untreated and plasma–treated polymer surfaces for 48 h. Figure 48b illustrates that the cell viability of L929 fibroblasts on plasma–treated surfaces ranged from 76% to 114%. Specifically, for PCL, cell viability remained comparable on both CO_2_ and O_2_ plasma–treated surfaces, ranging from 98% to 107%. In contrast, for P(3HB), cell viability slightly increased on O_2_ plasma–treated surfaces (down to 76%) compared to the untreated P(3HB) surface [64]. Table 31 summarizes the experimental plasma surface treatment conditions and corresponding biological results for Figure 48 in this section.

**Figure 49:**
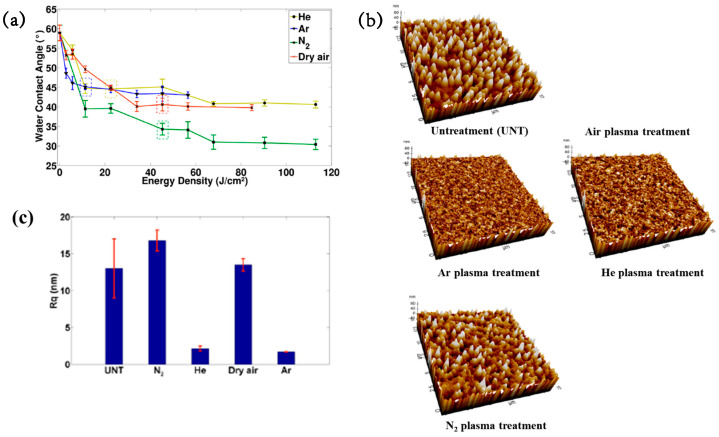
(**a**) WCA measurements of PEOT/PBT copolymer thin films as a function of energy density. (**b**) AFM images and (**c**) surface roughness (R_q_) before and after plasma treatment. Reproduced with copyright permission from Ref. [65].

To enhance cell surface interactions of the biomaterial, surface treatment of PEOT/PBT polymer thin films was conducted using a DBD-type plasma reactor under medium pressure (MP) with various gas environments (Ar, He, N_2_, and dry air), as reported by Cools et al. [65]. The PEOT/PBT copolymer thin films were first deposited by spin–coating at 2000 rpm with an acceleration of 400 rpm for 30 s, using a mixed solution of PEOT/PBT pellets and CHCl_3_ (2% *w*/*w*). Subsequently, the film surfaces were treated using a medium-pressure parallel–plate DBD reactor operated at an AC voltage of 50 kHz and a gas flow rate of 1 SLM under varying gas (Ar, He, N_2_, and dry air) atmospheres and energy densities. Figure 49a presents the wettability of PEOT/PBT thin films, as determined by WCA measurements, following plasma treatment with different gases and energy densities. For untreated PEOT/PBT films, the WCA was approximately 59°. With increasing plasma energy density, achieved by extending treatment time, the WCA decreased and eventually stabilized, indicating enhanced surface wettability. Furthermore, the WCA varied depending on the gas used during plasma treatment. To investigate the relationship between surface wettability and surface morphology, surface roughness was assessed using AFM. As shown in Figure 49b,c, films treated with He and Ar plasma exhibited reduced surface roughness compared to the untreated (UNT) films and those treated with N_2_ and dry air. These results indicate that surface roughness is also influenced by the type of plasma gas, and such changes can affect WCA properties [65].

**Figure 50:**
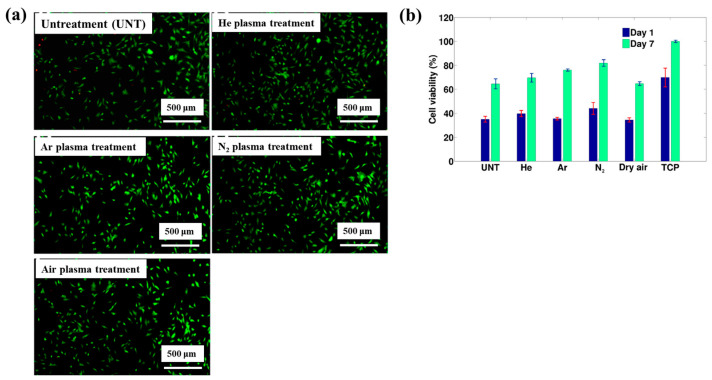
(**a**) Fluorescence microscopic images of human foreskin fibroblast (HFF) cells attached to plasma–treated PEOT/PBT films. (**b**) Cell viability of HFFs after 1 and 7 d of culture on plasma-treated PEOT/PBT films. Reproduced with copyright permission from Ref. [65].

Figure 50a shows the fluorescent micrographs of human foreskin fibroblast (HFF) cells attached to various plasma–treated PEOT/PBT films. In all cases, the attached cells were well distributed across the PEOT/PBT surface, which is attributed to the incorporation of polar groups (N_2_ or O_2_) introduced by plasma treatment [65]. Figure 50b presents the viability of HFF cells after 1 and 7 d of cultivation on plasma-treated PEOT/PBT films. Compared with the UNT sample, plasma treatment increased HFF cell viability by 25% after 1 week of cultivation. Table 32 summarizes the plasma surface treatment parameters and bacterial results corresponding to Figure 49 and Figure 50 in this section.

**Figure 51:**
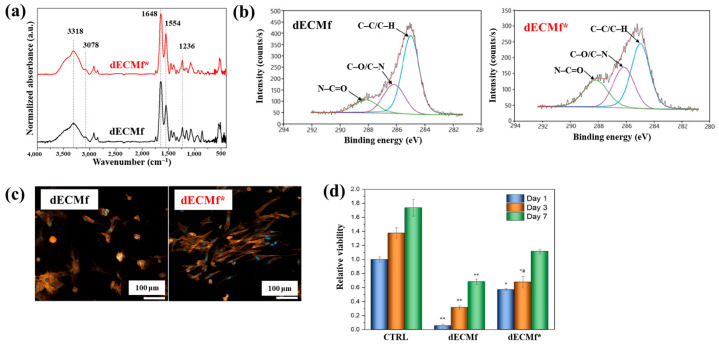
(**a**) FT–IR spectra, (**b**) XPS high–resolution C 1s spectra, (**c**) confocal microscopy images, and (**d**) cell viability of human dermal fibroblasts (HDFs) on decellularized extracellular matrix (dECM) films (dECMfs) and plasma–treated dECMf (dECMf*) after culturing for 1, 3, and 7 d. Reproduced with copyright permission from Ref. [66].

Lombardo et al. examined the surface treatment of a decellularized extracellular matrix (dECM) film (dECMf) using low–pressure cold plasma under N_2_/H_2_ mixed gas conditions [66]. To prepare the dECMf, 1.2 g of lyophilized dECMf was dissolved in 100 mL of 0.05 mol/L acetic acid. A 4 mL aliquot of the solution was cast into a Teflon mold, and the residual solvent was removed at room temperature (RT) by air–drying for 12 h. The dried film was then detached from the mold, yielding a thickness of approximately 50 μm. The samples were positioned 5 cm away from the plasma discharge region in a vacuum chamber and treated using microwave plasma at 100 kW, 300 mTorr, for 60 s under the N_2_/H_2_ gas mixture. These plasma–treated films are denoted as dECMf*. For both dECMf and dECMf* samples, FT–IR spectra exhibited 3318 and 3078 cm^−1^, corresponding to N–H bonds in the collagen amide structure. Peaks related to protein structures, including C=O and N–H combined with C–N bonds, were observed at 1648 and 1554 cm^−1^ (Figure 51a). XPS analysis further revealed changes in the chemical composition of the dECMf* surface. After plasma treatment, the N concentration and N/C ratio increased, as shown in Figure 51b. In addition, the dECMf* surface showed a higher O concentration and O/C ratio. The C 1s spectrum of both dECMf and dECMf* contained three peaks corresponding to C–C/C–H, C–O/C–N, and N–C=O bonds. Notably, for dECMf*, the proportion of C–C/C–H bonds decreased from 46.1% to 36.6%, while the C–N bond of the amine group increased from 17.8% to 21.2%, and the –N–C=O bond of the amide group increased from 10.4% to 15.6%. Cell adhesion and viability on dECMf and dECMf* were evaluated using human dermal fibroblasts (HDFs). Confocal micrographs obtained after 6 h of cultivation at 37 °C with 5% CO_2_ showed that HDFs adhered well to both surfaces (Figure 51c). Cell viability was assessed after 1, 3, and 7 d (Figure 51d). Although overall proliferation on both samples was lower than that of the CTRL, viability on dECMf* was higher at 1 d compared with dECMf. Furthermore, HDF proliferation on dECMf* increased steadily with incubation time from 1 to 7 d [66]. Table 33 summarizes the plasma surface treatment parameters and bacterial results corresponding to Figure 51 in this section.

**Figure 52:**
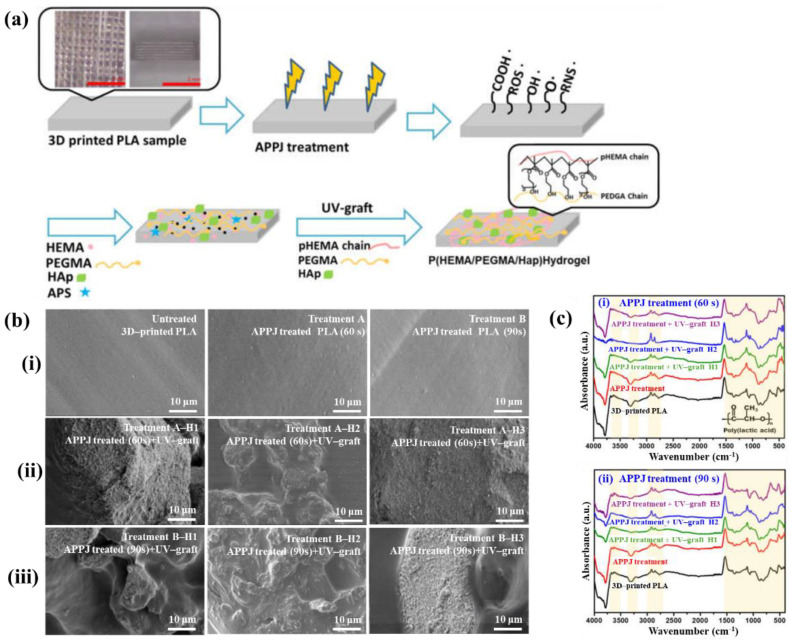
(**a**) Schematic of 3D–PLA surface treatment using APPJ method and UV–grafted hydrogels. (**b**) FE–SEM images of plasma–treated 3D–PLA samples (i–iii). (**c**) FT–IR spectra of plasma–treated 3D–PLA after (i) 60 s and (ii) 90 s. Reproduced with copyright permission from Ref. [67].

Liao et al. [67] investigated the cell viability of 3D-printed poly(lactic acid) (PLA) modified by APPJ treatment and UV–grafted hydrogels. Figure 52a shows a schematic of the 3D–PLA surface modification process using APPJ followed by UV–induced graft polymerization of hydrogels. The PLA substrate was first fabricated by 3D printing and then treated with APPJ. To form hydrogels on the surface, the APPJ-treated PLA was further subjected to UV surface graft polymerization. For this, the 3D–PLA samples were immersed in an aqueous monomer solution containing 10 wt% 2–hydroxyethyl methacrylate (HEMA), 5 wt% poly(ethylene glycol) methacrylate (PEGMA), 1 mol% ammonium persulfate (APS, (NH_4_)_2_S_2_O_8_), and 1 g hydroxyapatite (HAp) powder, as summarized in Table 34. After graft polymerization, the modified samples were washed with distilled water for 24 h to remove residual monomers. Detailed experimental conditions related to the surface treatments are summarized in Table 34 and Table 35. As shown in Figure 52b(i–iii), FE–SEM images illustrate the surface morphology of 3D–PLA samples after APPJ treatment and UV–grafted hydrogel modification. The pristine 3D–PLA exhibited a smooth surface, and no significant morphological changes were observed after APPJ treatment for 60 s and 90 s (Figure 52b(i)). In contrast, Figure 52b(ii,iii) shows that UV-grafted hydrogels formed continuous layers on the APPJ–treated 3D–PLA surface. For treatment conditions A and B, the H1 and H3 hydrogel coatings displayed surfaces with soft porous structures, whereas the H2 hydrogel exhibited a compact and dense morphology [67].

The chemical structures of APPJ–treated and UV–grafted hydrogel–modified PLA were analyzed using FT–IR spectroscopy. As shown in Figure 52c(i,ii), all modified samples exhibited peaks corresponding to PLA/HEMA/PEGMA/HAp components. In the pristine PLA, peaks were observed at 677, 997, 1117, 1700, and 2584–2920 cm^−1^, corresponding to C–O–C deformation vibrations, C–COO, C–O–C stretching, ester –C=O, and CH_3_ stretching, respectively. After APPJ treatment, peak intensity changes were observed, attributed to the incorporation of oxygen–containing functional groups, such as carbonyl (C=O) introduced by surface oxidation. Additional FT–IR peaks confirmed the presence of grafted polymers. Peaks associated with HEMA appeared at 1035, 1280, 1520–1530, 1642, 1738, and 3465 cm^−1^, corresponding to O–H bending, C–O stretching, CH_3_ asymmetric bending C=C methacrylate, C=O stretching, and C–O stretching, respectively. PEGMA-related peaks were identified at 1115 and 2855 cm^−1^, corresponding to ether and CH_2_ vibrations, respectively. Furthermore, Hap–related peaks were detected at 590, 1047, 1429, 1533, and 1644 cm^−1^, assigned to phosphate (PO_4_^3−^), carbonate (CO_3_^2−^), and O–H stretching bands [67].

**Figure 53:**
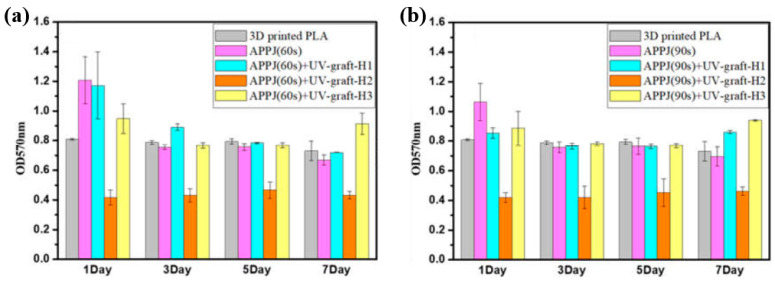
Cell viability of osteoblasts cultured on 3D–printed PLA surfaces after (**a**) treatment A and (**b**) treatment B, evaluated over 1 to 7 d. Reproduced with copyright permission from Ref. [67].

To evaluate cell adhesion on 3D–PLA samples treated with APPJ and UV–grafted hydrogels, osteoblast MG63 cells were seeded onto the modified substrates, and in vitro cytocompatibility was assessed. As shown in Figure 53a,b, after 7 d of cultivation, cell attachment and growth were significantly enhanced on APPJ–treated and UV–grafted 3D–PLA compared with untreated 3D–PLA [67]. Table 35 summarizes the experimental synthesis parameters and bacterial results corresponding to Figure 52 and Figure 53 in this section.

**Figure 54:**
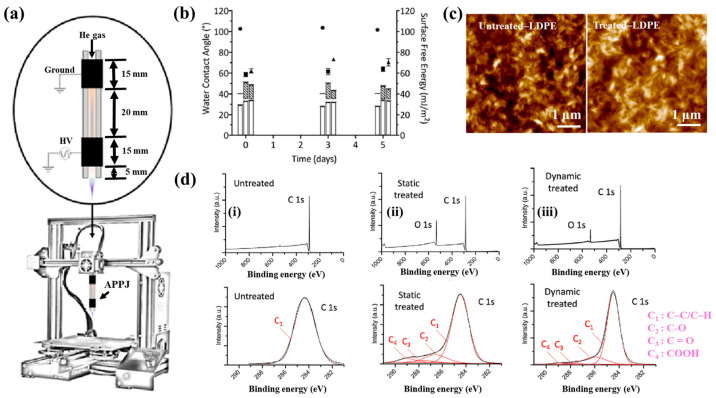
(**a**) Experimental setup of APPJ treatment combined with 3D printer. (**b**) WCA measurements of LDPE surfaces after different APPJ treatments. (**c**) AFM images of untreated and treated LDPE surfaces. (**d**) XPS survey and high–resolution C 1s spectra for various APPJ treatments. Reproduced with copyright permission from Ref. [68].

Lotz et al. [68] investigated the surface modification of low–density polyethylene (LDPE) using an APPJ system integrated into a 3D printer, as shown in Figure 54a. The LDPE surface was treated under plasma conditions consisting of He gas flow at 1.9 L/min, an applied voltage of 4.5 kV, and a frequency of 32.5 kHz, with the APPJ plasma nozzle positioned 5 mm from the polymer surface. Two APPJ treatment modes were applied: (i) static treatment, performed at a single point for 5 s, and (ii) dynamic treatment, performed by line scanning at 2500 mm/min with a 1 mm interval. Further details of the APPJ system were reported by Alavi et al. [68]. As shown in Figure 54b, the WCA values of the LDPE surface treated under two different APPJ conditions were measured. Both treatments yielded similar WCA values of approximately 60°. A slight increase in the average WCA was observed after 5 d of exposure to air. To investigate changes in surface morphology and roughness resulting from the two APPJ treatments, AFM analysis was conducted on untreated and APPJ–treated LDPE samples. Figure 54c presents the AFM images of LDPE samples treated under different APPJ conditions. The surface roughness of the dynamically treated sample was not significantly higher than that of the untreated sample.

The chemical properties of the treated surfaces were further analyzed using X-ray photoelectron spectroscopy (XPS). Figure 54d(i–iii) displays the XPS survey and high-resolution C 1s spectra for three sample types, namely untreated, statically treated, and dynamically treated samples. The oxygen concentrations of the untreated, statically treated, and dynamically treated surfaces were 0.6%, 15.9%, and 8.3%, respectively. The static APPJ treatment (5 s) resulted in approximately double the oxygen concentration on the polymer surface compared to the dynamic treatment (2.4 s). Relative to the untreated sample, APPJ treatment induced the incorporation of reactive oxygen functional groups on the polymer surface, and the extent of oxygen incorporation depended on treatment duration. The high–resolution C 1s spectra revealed the presence of carbon–containing functional groups (C–C/C–H, C–OH/R, C=O, and COOH), formed as a result of oxygen interaction during APPJ treatment [68].

**Figure 55:**
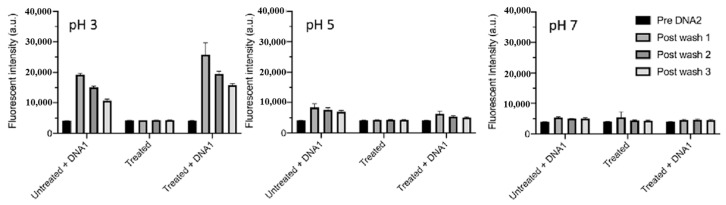
Fluorescence intensity of immobilized DNA on APPJ–treated LDPE surfaces under various pH conditions (pH 3, 5, and 7). Reproduced with copyright permission from Ref. [68].

To examine cell adhesion on the APPJ-treated LDPE polymer surface, two DNA molecules (DNA1 and DNA2) were seeded onto the plasma–treated surface and analyzed using fluorescence optical microscopy. In all experiments, DNA1 was immobilized first, followed by the introduction of DNA2 in combination with various proteins and hydrogels. Figure 55 presents the fluorescence intensity of immobilized DNA on the APPJ-treated LDPE surface under various pH conditions. The highest fluorescence intensity was observed at pH 3. This result is attributed to the increased number of free protons at lower pH levels, which renders the APPJ–treated surface more positively charged. As negatively charged DNA exhibits enhanced adhesion to positively charged surfaces, this electrostatic interaction likely explains the increased DNA binding observed at low pH. At pH 3, the “Treated + DNA1” samples exhibited significantly higher fluorescence intensity compared to the “Untreated + DNA1” samples, indicating that the APPJ–treated surface facilitates stronger biomolecular binding [68]. Table 36 summarizes the plasma surface treatment conditions and bacterial results corresponding to Figure 54 and Figure 55 in this section.

**Figure 56:**
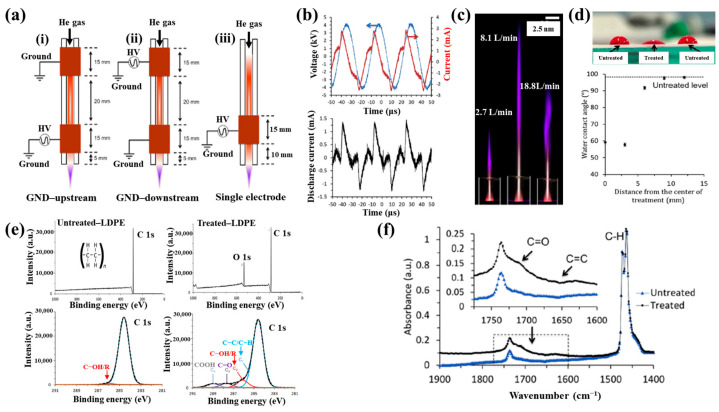
(**a**) Schematic of double– ((i) GND–upstream, (ii) GND–downstream) and (iii) single–electrode configurations for APPJ treatment system. (**b**) Voltage, total current, and discharge current of APPJ system using GND–downstream configuration. (**c**) Photographic image of plasma plums generated in double-electrode (GND–upstream) setup under different gas flow rates (2.7, 8.1, and 18.8 L/min). (**d**) WCA measurements of polymer surfaces before and after APPJ treatment. (**e**) XPS survey, high–resolution C 1s spectra, and (**f**) FT–IR spectra of untreated and plasma–treated LDPE surfaces. Reproduced with copyright permission from Ref. [69].

Alavi et al. [69] investigated the covalent immobilization of proteins on low-density polyethylene (LDPE) and poly–ε caprolactone (PCL) surfaces treated using an APPJ. Figure 56a illustrates the schematic diagram of the APPJ treatment system, including double-electrode configurations ((i) GND–upstream and (ii) GND–downstream) as well as (iii) a single-electrode configuration. Figure 56b presents the voltage, total current, and discharge current profiles of the plasma discharge generated by the APPJ system with the double-electrode (GND–downstream) configuration. A pronounced single spike peak was observed in each half–cycle of the current signal, indicating strong plasma discharge and an associated increase in current between the electrodes. Figure 56c shows a photographic image of the plasma plums generated under the double-electrode (GND–upstream) configuration with varying He gas flow (2.7, 8.1, and 18.8 L/min). The length of the plasam plume increased with higher He gas flow rates until turbulence appeared [69]. Based on plasma characterization under various flow conditions, an optimized gas flow rate of 1.9 L/min was selected for APPJ treatment. Figure 56d shows the WCA measurements of untreated and APPJ-treated LDPE surfaces. The WCA of the untreated LDPE surface was measured at 98.3°, which decreased to approximately 60° after APPJ treatment at a 3 mm distance, indicating increased surface wettability [69]. Figure 56e displays the XPS survey and high-resolution C 1s spectra of untreated and APPJ–treated LDPE surfaces. The elemental composition analysis revealed that the carbon concentration decreased from 100% (untreated) to 85% (treated), while the oxygen concentration increased from 0% to 15% following APPJ treatment. This rise in oxygen content is attributed to surface oxidation induced by the plasma. The high-resolution C 1s spectrum of the APPJ-treated LDPE surface identified the presence of C−C/C−H, C−OH/R, C=O, and COOH functional groups. Furthermore, as shown in Figure 56f, FT–IR spectroscopy confirmed the presence of polyethylene–related peaks on the untreated LDPE surface. In contrast, the APPJ–treated LDPE surface exhibited new absorption bands corresponding to carbonyl (C=O) and alkene (C=C) functional groups, indicating successful chemical modification through plasma treatment [69].

**Figure 57:**
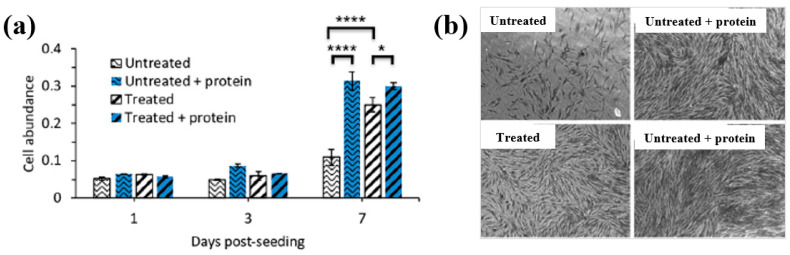
(**a**) Cell abundance on untreated and plasma–treated LDPE surfaces at 1, 3, and 7 d after seeding. (**b**) Cell images of untreated and treated LDPE surfaces after 7 d. Reproduced with copyright permission from Ref. [69].

To examine cell adhesion on the LDPE surface, GM3348 human dermal fibroblast cells were seeded onto the LDPE samples treated with different APPJ conditions and fibronectin coatings, and observed using fluorescence optical microscopy. As shown in Figure 57a,b, after 7 d of incubation, the cell numbers significantly increased on APPJ–treated and fibronectin–coated LDPE surfaces [69]. Table 37 summarizes the plasma treatment parameters and corresponding bacterial results related to Figure 56 and Figure 57 in this section.

Furthermore, additional information related to plasma surface treatments and biomedical experiments from recent studies, without copyright permission approval, is summarized and presented in Table 38 [70,71].

Table 39 summarizes the plasma surface treatments of polymer films for biomedical applications discussed in this section.

## 3. Plasma Synthesis and Surface Treatment of Metal NPs for Biomedical Applications

**Figure 58:**
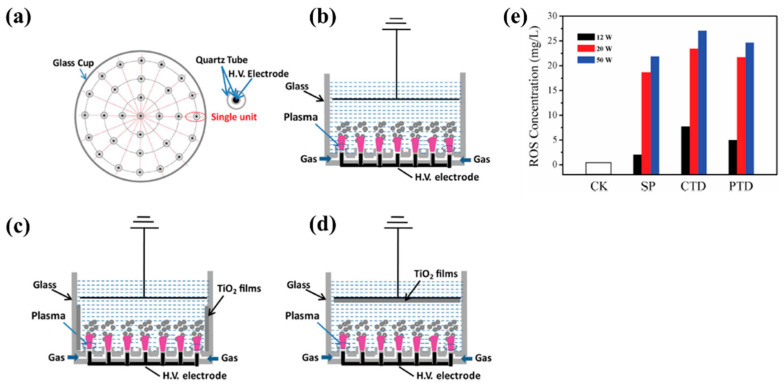
Schematic of air micro plasma jet array operated under AP conditions by Zhou et al. (**a**) Top view of micro plasma device, cross–section of (**b**) single–discharge (SD) system without TiO_2_, (**c**) SD system with circular TiO_2_ film (CTD), and (**d**) SD system with plate-like TiO_2_ film (PTD). (**e**) ROS concentrations in solutions treated with O_2_ plasma using SD, PTD, and CTD reactors at different plasma powers (12, 20, and 50 W). Reproduced with copyright permission from Ref. [72].

Zhou et al. [72] investigated the antibacterial efficiency of a plasma/TiO_2_ photocatalysis system using an APP solution process. Figure 58a,b illustrate the AP micro plasma jets used to inactivate *E. coli* in solution. The system consisted of 29 micro plasma jets arranged at the bottom of the reactor, with each plasma tube comprising an inner electrode, a quartz sleeve surrounding the inner electrode, and an outer quartz tube, separated by an 80 μm gap. Plasma was generated via DBD when various gases (He, nitrogen (N_2_), air, or oxygen (O_2_)) were introduced at a flow rate of 2 standard liters per minute (LPM). The ground electrode, a 30 mm stainless steel disk, was positioned 10 mm above the plasma tubes [72]. Plasma discharge was achieved using a peak voltage of 4.5 kV at a frequency of 9.0 kHz. TiO_2_ nanotube films were synthesized electrochemically and applied to three reactor configurations (SD, CTD, and PTD). The SD reactor lacked a TiO_2_ film. In the CTD reactor, a circular TiO_2_ film was affixed to the inner wall of the cylindrical plasma reactor, as shown in Figure 58c, whereas in the PTD reactor, a plate-like TiO_2_ film was attached to the surface of the stainless steel ground electrode, as depicted in Figure 58d. Figure 58e presents the total ROS concentrations in plasma–treated solutions for the three reactors (SD, CTD, and PTD) under increasing power conditions using O_2_ plasma. The CTD reactor exhibited the highest ROS concentration, reaching a 50 W plasma power, compared to 24.7 mg/L for the SD reactor and 21.8 mg/L for the PTD reactor. The reduced efficiency of the PTD reactor is attributed to the degradation of the TiO_2_ film structure due to thermal effects during repeated plasma treatments (six cycles). Since rutile TiO_2_ possesses lower photocatalytic activity than anatase, the phase transformation to rutile in the PTD reactor further reduced bacterial inactivation efficiency [72].

**Figure 59:**
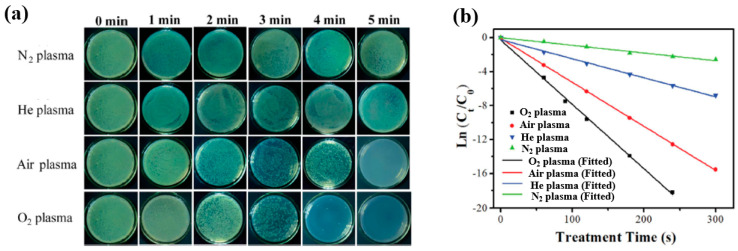
(**a**) Viability of *E. coli* treated with plasma under gas conditions (N_2_, He, air, and O_2_) for 0–5 min at V_p_ = 4.5 kV. (**b**) Comparison of experimental values with theoretical kinetic model over plasma treatment time. Reproduced with copyright permission from Ref. [72].

Figure 59a presents the viability of plasma–treated *E. coli* under varying plasma treatment durations (0, 1, 2, 3, 4, and 5 min) and gas conditions (N_2_, He, air, and O_2_) with fixed operating parameters (4.5 kV, 2 SLM flow rate, and an initial concentration of 10^6^ colony-forming units (CFU)/mL) [72]. The results show that *E. coli* treated with O_2_ plasma exhibited the highest inactivation efficiency compared to treatments using He or N_2_ plasma. Additionally, the decontamination effect improved significantly with increasing treatment duration, confirming that antibacterial efficacy is strongly dependent on both plasma exposure time and gas composition. Notably, plasma treatments with O_2_ and air achieved over 99% antibacterial performance. To further analyze the inactivation kinetics, a linear regression plot based on a kinetic model was generated. Figure 59b shows the experimental ln(C_t_/C_0_) values alongside theoretical predictions for different gases (N_2_, He, air, and O_2_) and plasma treatment durations. The ln(C_t_/C_0_) values decreased with increasing treatment time, with O_2_ plasma demonstrating the highest *E. coli* killing efficiency, attributed to its high ROS density [72]. Table 40 summarizes the experimental plasma surface treatment conditions and corresponding antibacterial results for Figure 58 and Figure 59 in this section.

**Figure 60:**
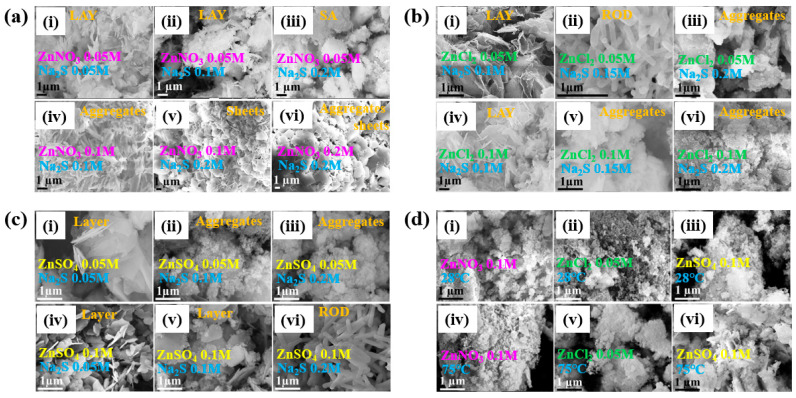
Surface morphology of zinc sulfide nanoparticles (ZnS NPs) synthesized using AP soft jet plasma under varying precursor conditions: (**a**(**i**–**vi**)) using zinc nitrate (Zn(NO_3_)_2_) and sodium sulfide (Na_2_S), (**b**(**i**–**vi**)) using zinc chloride (ZnCl_2_), and (**c**(**i**–**vi**)) using zinc sulfate (ZnSO_4_). (**d**(**i**–**vi**)) Synthesized ZnS was prepared using conventional wet chemical methods with different precursors (Zn(NO_3_)_2_, ZnCl_2_, and ZnSO_4_) at two temperatures (28 and 75 °C). Reproduced with copyright permission from Ref. [73].

**Figure 61:**
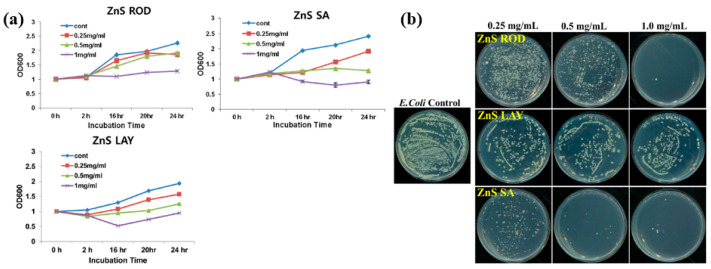
(**a**) Bacterial growth inhibition and (**b**) reduction in bacterial colony count of *E. coli* using ZnS NPs synthesized by using AP soft jet plasma. Reproduced with copyright permission from Ref. [73].

Ananth et al. [73] investigated the effect of various ZnS NP morphologies, synthesized using a plasma jet device, on bacterial cell growth inhibition, as shown in Figure 60 and Figure 61. ZnS NPs were first synthesized using an AP soft–jet plasma system. To characterize the influence of different zinc precursors on NP morphology, ZnS NPs were synthesized using both the AP soft–jet plasma and a conventional wet chemical method, employing three distinct zinc precursors including Zn(NO_3_)_2_, ZnCl_2_, and ZnSO_4_ [73]. The AP soft–jet plasma setup and detailed synthesis conditions were described previously by Ananth et al. [73]. In the synthesis procedure, 1 mL each of a zinc precursor solution and Na_2_S solution was mixed in a glass reactor and treated with AP soft–jet plasma for 1 h, maintaining a 10 mm gap between the solution surface and the electrode tip. After plasma treatment, ZnS NPs were collected, washed with deionized water, centrifuged, and dried in a heating oven at 300 °C. As shown in Figure 60a(i–vi), ZnS layers synthesized using different concentrations of Zn(NO_3_)_2_ and Na_2_S predominantly exhibited agglomerated spherical and two–dimensional (2D) sheet structures. The ZnS sheets measured over 1 mm in length and less than 100 nm in width (Figure 60a(i)), with some exceeding 2 μm along the edges (Figure 60a(v)). Similarly, ZnS NPs were synthesized using ZnCl_2_ precursors at varying concentrations. Figure 60b(i–vi) shows two dominant morphologies, namely rod-like structures (Figure 60b(ii)) and layered aggregated architectures (Figure 60b(iv)). Furthermore, ZnS NPs were synthesized with ZnSO_4_ as the precursor. Figure 60c(i–vi) shows the three major forms, namely large sheets (Figure 60c(i,iv,v)), aggregates (Figure 60c(ii,iii)), and rod–like structures (Figure 60c(vi)).

For comparison, Figure 60d(i–vi) shows ZnS NPs synthesized via a conventional wet chemical method using the same zinc precursors at temperatures of 28 and 75 °C. In all cases, the products consisted of agglomerated spherical NPs with a one–dimensional (1D) structural morphology [73].

Figure 61a illustrates the antibacterial inhibition effects of three ZnS NP morphologies (ZnS ROD, ZnS LAY, and ZnS SA) on *E. coli*. The sample without ZnS NP treatment served as the control group. All ZnS NP types demonstrated substantial inhibition of *E. coli* growth compared to the control [73]. The inhibition was most pronounced at a concentration of 1 mg/mL for all NP types, indicating a strong concentration–dependent antibacterial effect. The antibacterial efficacy was further evaluated using the spread plate method on agar plates inoculated with *E. coli*. As shown in Figure 61b, all ZnS NPs reduced bacterial colony counts, with a notable reduction observed at 1 mg/mL ZnS ROD and 0.5 mg/mL ZnS SA [73]. Table 41 summarizes the experimental plasma synthesis conditions and antibacterial results corresponding to Figure 60 and Figure 61 in this section.

**Figure 62:**
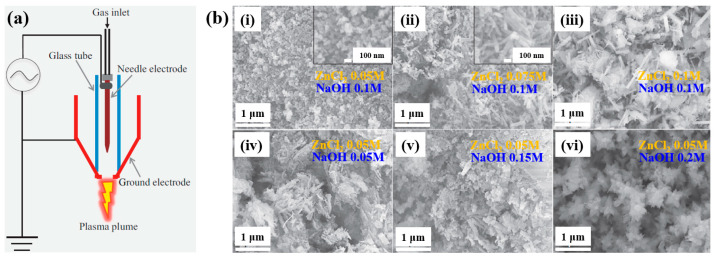
(**a**) Schematic of AP soft jet plasma setup used for ZnO NP synthesis from ZnCl_2_ and hydroxide (NaOH) precursors. (**b**) SEM images of ZnO NPs synthesized at different precursor concentrations, including (i–iii)) ZnCl_2_ concentrations (0.05, 0.075, and 0.1 M) at 0.1 M NaOH and (iv–vi) NaOH concentrations (0.05, 0.15, and 0.2 M) at 0.05 M ZnCl_2_. Reproduced with copyright permission from Ref. [74].

Ananth et al. investigated the morphology of ZnO NPs synthesized using AP soft jet plasma under conditions of varied precursor type and mixing ratio of ZnCl_2_ and NaOH [74]. The synthesis process was carried out in an AP soft jet plasma system operated in pulse mode at the Plasma Bioscience Research Center, Kwangwoon University, Korea. As shown in Figure 62a, an alternating current (AC) voltage was applied to the Cu needle electrode, while the outer electrode was grounded. The inner electrode was insulated within a glass tube, and an air flow of 2 L/min was maintained under pulsed power conditions [74]. SEM images of ZnO NPs synthesized with a zinc nitrate precursor are presented in Figure 62b. With increasing ZnCl_2_ (0.05, 0.075, and 0.1 M) at fixed 0.1 M NaOH, ZnO NPs exhibited distinct morphologies, including non-uniform cube-like structures, uniform particles, and aggregated spherical nanorods (RO) (Figure 62b(i–iii)). Particle size distribution was 400–450 nm. To further investigate the effect of NaOH concentration, ZnO NPs were synthesized using 0.05, 0.15, and 0.2 M NaOH at fixed 0.05 M ZnCl_2_. With increasing NaOH concentration, the ZnO NPs developed progressively denser ROs, nanosheets (SH), and flower-like structures (FL) (Figure 62b(iv–vi)) [74].

**Figure 63:**
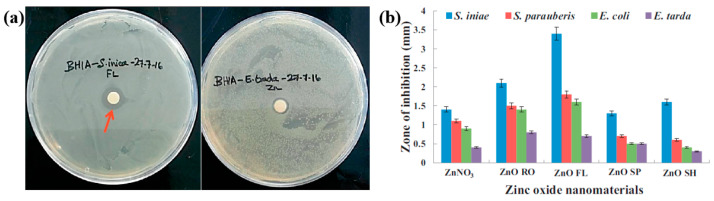
(**a**) Photographic images of zone of inhibition (ZOI) and (**b**) comparison of ZOI lengths for various ZnO NPs against four bacterial strains. Reproduced with copyright permission from Ref. [74].

The antibacterial properties of the synthesized ZnO NPs were evaluated against four bacterial strains, namely *E. coli*, *S. iniae*, *S. parauberis*, and *E. tarda*. Figure 63a presents photographic images of the zone of inhibition (ZOI) measured using the disc diffusion method [74]. As shown in Figure 63b, the ZOI lengths were compared across the different ZnO NP morphologies and bacterial strains. The ZnO FL sample exhibited the highest ZOI length of 3.4 mm, while the ZnO RO sample showed a ZOI length of 2.1 mm against *S. iniae*. Overall, both ZnO FL and RO samples demonstrated significant antibacterial activity against all tested strains compared with ZnO SH and SP samples [74].

A summary of the plasma synthesis conditions and antibacterial results corresponding to Figure 62 and Figure 63 is provided in Table 42.

**Figure 64:**
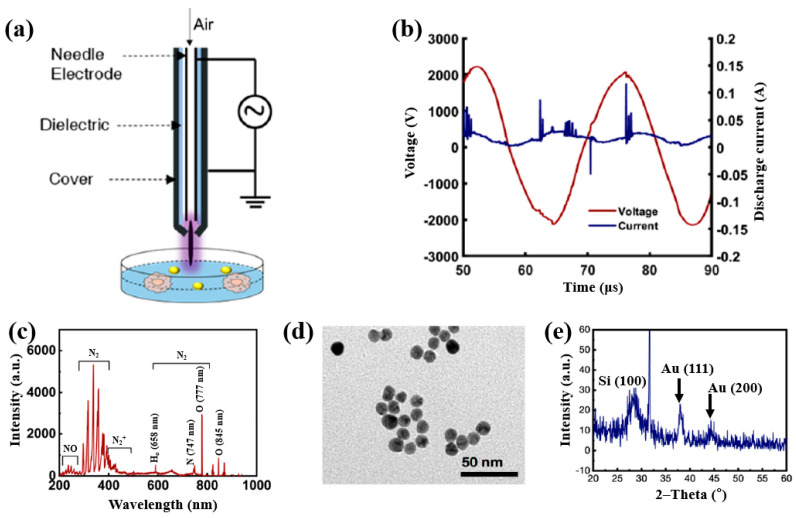
(**a**) Schematic of CAP–based soft jet plasma system for gold quantum dot (AuQD) synthesis. (**b**) Current–voltage waveform and (**c**) OES spectrum of plasma discharge during AuQD synthesis. (**d**) TEM images and (**e**) XRD analysis of AuQDs on silicon wafers. Reproduced with copyright permission from Ref. [75].

Kaushik et al. investigated the cytotoxic effects of gold quantum dots (AuQDs) combined with CAP on brain cancer cells (U373) [75]. Figure 64a shows a schematic of the soft jet plasma system used for AuQD synthesis and CAP co–treatment prior to the cell studies. The system comprised a needle electrode placed inside a cylindrical glass tube and encased in a 3D–printed plastic shell. Air was used as the process gas at a flow rate of 1.0 lpm. AuQDs were synthesized by using soft–jet plasma with H[AuCl_4_]·3H_2_O and trisodium citrate dihydrate (N_3_C_6_H_5_O_7_, 1%). The current–voltage waveform and the OES spectrum of plasma discharge during AuQD synthesis are presented in Figure 64b,c. Plasma discharge was generated under an 11% pulse duty cycle at a peak voltage of 2.2 kV, a frequency of 42 kHz, and a current of 100 mA [75]. The OES spectrum revealed peaks corresponding to ROS, RNS, and oxygen species. TEM analysis confirmed that the synthesized AuQDs were spherical with an average particle size of 4–5 nm (Figure 64d). XRD analysis (Figure 64e) revealed peaks at 38.17° and 44.45°, confirming the Au crystal phase with a face-centered cubic (FCC) structure [75].

**Figure 65:**
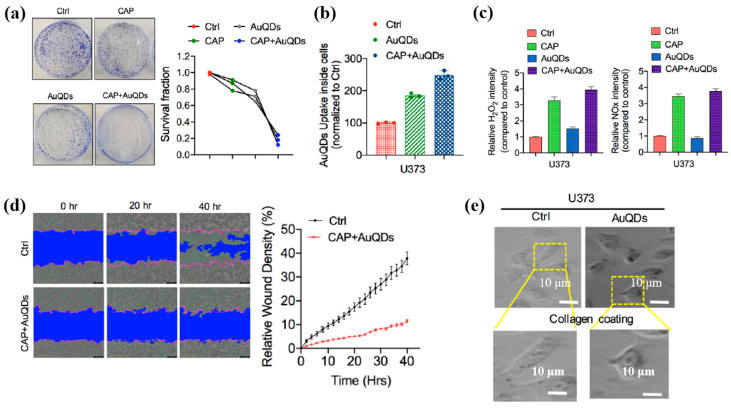
(**a**) Clonogenic formation and survival fraction of U373 brain cancer cells treated with AuQDs (25 nM) or CAP for 200 s. (**b**) Quantification of AuQD or CAP uptake by U373 cells. (**c**) Comparison of nitrogen species (NO_X_) and H_2_O content in U373 cells. (**d**) Wound healing behavior of U373 cells co-treated with AuQDs (25 nM) and CAP (200 s) at 20 h and 40 h. (**e**) SEM images of untreated (Ctrl) and AuQD–CAP co-treated U373 cells after 11 d. Reproduced with copyright permission from Ref. [75].

To further investigate the cytotoxic effects on brain cancer cells (U373), cells were seeded in the AuQD and CAP co–treatment sample following individual treatments with AuQDs and CAP. For this combined co-treatment, cells were incubated with 25 nM AuQDs for 5 h, followed by exposure to air soft–jet plasma for 200 s. As shown in Figure 65a, colony formation in U373 cells was significantly reduced after treatment with either AuQDs (25 nM) or CAP (200 s). To assess the biocompatibility and impact of the co–treatment on cancer cell behavior, a wound scratch assay was conducted to evaluate cell migration. As illustrated in Figure 65b, cellular uptake in brain cancer cells increased under the combined AuQD and CAP treatment compared to treatment with AuQDs alone [75]. To elucidate the involvement of RONS, intracellular levels of RONS and H_2_O_2_ were measured post treatment. Figure 65c demonstrates that the intensities of nitrogen species (NO_X_) and H_2_O_2_ were elevated in the co–treatment group relative to the untreated control (ctrl). Additionally, Figure 65d reveals a significant reduction in wound healing, indicating suppressed cancer cell migration following co–treatment with AuQDs and CAP compared to the control (ctrl). As shown in Figure 65e, the co–treatment also contributed to a decrease in both cell size and proliferation [75]. These findings suggest that enhanced cellular uptake may be mediated by RONS scavengers induced by the combined AuQD and plasma treatment. Furthermore, the observed inhibition of cell migration may be attributed to reduce cell adhesion caused by the co–treatment, thereby suppressing metastatic potential. Table 43 summarizes the experimental parameters for plasma synthesis and the associated cellular results corresponding to Figure 64 and Figure 65.

Table 44 summarizes the plasma-based synthesis and plasma surface treatments of metal NPs for biomedical applications as discussed in this section.

## 4. Recent Research Trends of CAP-Based APPJ Technique

The CAP process can be mainly generated under vacuum, low pressure, or APP. In recent years, the APP method has received significant attention for the plasma synthesis and surface modification of polymer films and metal NPs across various industrial applications [76,77,78,79,80,81]. In particular, APPJ devices are mainly used in the fields of polymer and metal NP plasma synthesis and polymer surface treatment [82,83,84,85]. Table 45 summarizes the recent review articles related to CAP–based APPJ technique in this section.

## 5. Conclusions and Future Perspectives

In summary, the plasma synthesis and surface treatment of polymer films and metal NPs are mainly performed using the CAP process due to its unique advantages, such as simple equipment, low cost, short process time, and lack of heating. In this review, we examined and summarized recent studies related to plasma synthesis and surface treatment for the surface functionalization of polymer and metal NPs in plasma biomedical applications. First, we focused on the plasma synthesis of polymer films using various CAP techniques. For applications involving cell adhesion and antifouling in biomedical fields, plasma–deposited polymer films must incorporate diverse functional groups, such as hydroxyl, carboxyl, and amino groups, through the selection of appropriate precursors and plasma gas compositions. Second, we examined the plasma surface treatment of polymer films using CAP to enhance cell adhesion and proliferation in biomedical contexts. This is achieved by controlling surface properties, such as roughness, wettability, and the incorporation of oxygen–containing functional groups. In addition, polymer surfaces must exhibit superhydrophilicity and high surface energy, which can be attained by generating nanostructured surfaces via plasma surface treatment. Finally, we provided an overview of several studies on the plasma synthesis and surface modification of metal NPs for biomedical applications. These NPs can serve as reactive scavengers for cancer cell diagnosis and treatment, owing to their unique physicochemical properties, surface area–to–volume ratios, elevated surface energy, strong surface plasmon resonances (SPRs), and high density of dangling bonds. Accordingly, various CAP processes related to plasma biomedical applications, including, DBD and plasma jet (APPJ) systems, were discussed in relation to their role in the plasma synthesis and surface functionalization of both polymer and metal NPs for controlling various surface properties (roughness, wettability, and functionalization). Furthermore, this review summarized the effects of CAP treatment on material properties and the biomedical performance of CAP-treated polymer and metal NPs.

In the near future, CAP technology will likely experience strong growth in various biomedical fields. This CAP–based plasma technology has been commercialized in tissue treatment for skin care. However, it is not yet commercialized in medical fields such as cancer therapy and dental clinics. For the industrialization of plasma technology in the medical field, several challenges preventing biomedical applications of this CAP process must be overcome, i.e., the low plasma density, industry upscaling, and biocompatibility for safety and medical approval. Thus, it is important to encourage and assist further investigations and research on the CAP process in order to overcome these challenges.

## Figures and Tables

**Table 1:** Summary of experimental plasma synthesis and antibacterial results corresponding to Figure 1, Figure 2 and Figure 3 [34,35].

Precursor	Mixture with CS powder, 2% acetic acid solution, and AgNO_3_ (10 mM)
Synthesis method	APM on the solution
Applied voltage (kV)	4
Gas type	He
Gas flow (sccm)	25
Plasma process time (min)	10
AgNP/CS film fabrication	Drop-casting
Functional group of AgNP/CS	N–H (amid), ROS (–OH, C–O)
Bacteria type	*E. coli*, S. aureus
Inhibition zone	9 mm at 4 mM AgNPs/CS under *E.coli* 6 mm at 4 mM AgNPs/CS under *S.aureus*

**Table 2:** Summary of experimental plasma synthesis and bacterial results corresponding to Figure 4 and Figure 5 [12].

Precursor	3-(Aminopropyl)triethoxysilane (APTES)
Chemical formula	(CH_3_CH_2_O)_3_–Si–CH_2_CH_2_CH_2_NH_2_
Synthesis method	APPJ
Plasma power (W)	300
Frequency (kHz)	68
Plasma gas	N_2_
Plasma gas flow (SLM)	80
Precursor carrier gas	N_2_
Precursor carrier gas flow (SLM)	1.5
Number of plasma passes	0, 4, 8, 12, 24, 48, 72, 96
Substrate speed (mm/s)	50
Functional group of pAPTES film	Amine (NH_2_), and oxygenated carbon groups
Roughness (R_a_, nm)	S0p (4.8) and S72p (55.1)
WCA (°)	S0p (54.7) and S72p (56.6)
Bacterial type	*P. aeruginosa*
Bacterial inoculum (CFU/mL)	10^6^

**Table 3:** Summary of experimental plasma synthesis and antibacterial results corresponding to Figure 6 and Figure 7 [36].

Precursor	Essential oil (D–limonene, C_10_H_16_)
Synthesis method	APPJ
Applied voltage (kV)	3
Plasma gas	Ar
Plasma gas flow (sccm)	130
Plasma deposition time (min)	1, 3, 5, 7, 7, 9
Deposition rate (nm/s)	0.8
Functional group of AP–PP–lim thin film	Oxygenated carbon groups (C–O, C=O, O–C=O)
Roughness (R_q_, nm)	0.27
Roughness (R_a_, nm)	0.23
WCA (°)	90 at 1 min
Bacterial type	Gram-negative (*E. coli*)
Bacterial inoculum (CFU/mL)	10^5^
Number of te attached bacteria	4.3 × 10^5^ (substrate (control)) 2.4 × 10^4^ (AP–PP–lim film)

**Table 4:** Summary of experimental plasma synthesis and antibacterial results corresponding to Figure 8 and Figure 9 [37].

Precursor	AAc, AAm, AAOH, and OD
Synthesis method	LPP
Plasma power (W)	3 (ppAAc, ppAAOH, ppOD), 5 (ppAAm)
Precursor gas flow (sccm)	4 (ppAAc, ppAAOH, ppOD), 5 (ppAAm)
Plasma deposition time (min)	20 (ppAAc, ppAAOH), 35 (ppAAm)
Deposition rate (nm/min)	1.3 (ppAAc), 1 (ppAAm), 2 (ppAAOH)
Functional group of PP films	COOH/R (carboxyl or ester) at ppAAc COH/R (alcohol/ether) at ppAAOH
WCA (°)	72 (ppAAc at 25%AAc), 56 (ppAAm), 51 (ppAAOH)
Cell type	keratinocytes, fibroblasts, and endothelial cells

**Table 5:** Summary of plasma synthesis parameters and antibacterial test results corresponding to Figure 10 and Figure 11 [21,38].

	Ref. [21]	Ref. [38]
Precursor	2–ethyl–2–oxazoline (C_5_H_9_NO)	
Synthesis method	APTD (DBD–type)	
Plasma power (W)	55	
Frequency (kHz)	6	
Plasma gas	N_2_	
Plasma gas flow (sccm)	500	
Precursor gas flow (sccm)	50–500	
Plasma deposition time (min)	23	
Functional group of POx film	N–H bonds	
POx film thickness (μm)	0.6 (150 °C), 1.2 (500 sccm)	
R_q_ and R_a_ roughness (nm) of POx film	3.2, 2.1	4.8, 3.7
WCA (°)	33.4 (bare substrate), 24.1 (POx film)	
Bacterial type	*S. aureus*, *E. coli*	
Number of injected bacterial (CFU/mL)	1.7 × 10^5^ (*S. aureus*) 2.4 × 10^6^ (*E. coli*)	8.9 × 10^5^ (*S. aureus*) 3.4 × 10^6^ (*E. coli*)
Number of attached bacteria (CFU/cm^2^) at 150 °C, 500 sccm	1.3 × 10^6^ (substrate) 1.7 (*S. aureus*) 3.3 (*E. coli*)	2.0 × 10^5^ (substrate) 1.6 (*S. aureus*) 5.4 (*E. coli*)

**Table 6:** Summary of experimental plasma synthesis and antibacterial results corresponding to Figure 12 and Figure 13 [39].

Precursor	Mixture solution (2 mL) of 0.2 mM H[AuCl_4_] and 0.05 mM PDA
Synthesis method	AP solution plasma
Applied voltage (kV)	4.8
Frequency (kHz)	28
Plasma gas	Ar
Plasma gas flow (sccm)	700
Plasma synthesis time (min)	5
Size of PDA coated Au NPs (nm)	42
Thickness of PDA coating (nm)	4
Cell type	Breast carcinoma cells
Number of cells (×10^4^ cells/mL)	100–150 (before treatment) <20 (after treatment at 200 μM)

**Table 7:** Summary of experimental plasma synthesis and antibacterial results corresponding to Figure 14 and Figure 15 [41].

Precursor	Heptylamine (H_3_C(CH_2_)_5_CH_2_NH_2_)
Synthesis method	APPJ
Applied voltage (kV_p–p_)	8
Frequency (kHz)	10
Plasma gas	He
Plasma gas flow (sccm)	500
Precursor vapor gas flow (sccm)	20, 50, 100 by He gas
Plasma synthesis time (min)	10
WCA (°)	77.2 (UT–PS) >40 (edge) or <20 (center) for ppHEPTYL–HE–PS
Roughness (R_q_, nm)	0.6 (UT–PS), 0.4 (ppHEPTYL–HE–PS)
Cell type	Human LEC line
Number of cells (cells/cm^2^)	1 × 10^4^

**Table 8:** Summary of experimental plasma synthesis and antibacterial results corresponding to Figure 16 and Figure 17 [42].

Precursor	Acetic acid
Substrate	PP, PET, PLGA
Synthesis method	LPP
Plasma power (W)	10, 30, 50
Frequency (MHz)	13.56
Plasma synthesis time (min)	10
Functional groups of PP film	C–H, C=O, C–C
WCA (°)	85.5 (Untreated PP), 58.0 (Untreated PLGA) 12.1 (PP with AAP), 28.8 (PLGA with AAP)
Cell type	Mouse embryonic stem (ES)
Number of incubated cells (μL)	100

**Table 9:** Summary of experimental plasma synthesis and antibacterial results corresponding to Figure 18 [43].

Precursor	Allylamine, hexamethyldisiloxane (HMDSO)
Synthesis method	PECVD
Substrate	Metal—Al foil
Plasma powder (W)	10 (HMDSO), 100 (allyamine)
Frequency (MHz)	13.56
Plasma synthesis time (min)	20
Cell type	Mouse fibroblasts

**Table 10:** Summary of experimental plasma synthesis and antibacterial results corresponding to Figure 19a–d [44].

Precursor	Cyclopropylamine (CPA, C_3_H_7_N)
Synthesis method	LPP–RF capacitively coupled plasma
Plasma power (W)	100
Frequency (MHz)	13.56
Plasma discharge mode	Pulse (CPA40, CPA43), continuous (CPA42)
Plasma gas	Ar
Plasma gas flow (sccm)	28
Plasma synthesis time (min)	60
Functional groups of PP film	Amide (N–C=O, C=N), imine/nitride (C=N)
Cell type	Human dermal fibroblasts (HDFs), endothelial cells
Number of incubated cells (cells/mm^2^)	15

**Table 11:** Summary of experimental plasma synthesis and antibacterial results corresponding to Figure 20 and Figure 21 [45].

Precursor	2–methyl–2–oxazoline (C_4_H_7_NO)
Synthesis method	LPP
Plasma gas	Air
Plasma deposition time (s)	30, 50
Gas for plasma treatment	O_2_, air
Plasma treatment time (s)	30
Functional groups of POx film	Oxygen containing polar groups (COOH)
Cell type	Prostate carcinoma

**Table 12:** Summary of experimental plasma synthesis and antibacterial results corresponding to Figure 22 and Figure 23 [46].

Precursor	Acetylene (C_2_H_2_)
Synthesis method	LPP RF–PECVD
Plasma power (W)	10, 20, 30
Frequency (MHz)	13.56
Plasma gas	N_2_
Plasma gas flow (sccm)	40
Precursor vapor gas flow (sccm)	20
Functional groups of L–PPA:N film	Amine (NH_2_), alkyl, carboxyl
Roughness (R_q_, nm) of L–PPA:N film	1.0 (10 W), 2.9 (20 W), 3.8 (30 W)
Cell type	Mesenchymal stem cells (MSCs)

**Table 13:** Summary of experimental plasma synthesis and antibacterial results corresponding to Figure 24 and Figure 25 [47].

Precursor	Acetylene (C_2_H_2_)
Synthesis method	RF plasma
Plasma power (W)	50
Substrate	Titanium foil
Plasma gas	Ar
Plasma gas flow (sccm)	15
N_2_/C_2_H_2_ gas flow ratio	0, 0.5, 1, 2, 5
Functional groups of IPP film	Amide
Cell type	Osteoblast cells (mouse long bones)
Number of incubated cells (cells/well)	10^3^∼10^4^

**Table 14:** Summary of experimental plasma synthesis and antibacterial results corresponding to Figure 26 [48].

Polymer materials	pPMeO_x_, pPEtO_x_, pPPrO_x_, and pPBuO_x_
Precursor	Four 2–alkyl–2–oxazolines (MeO_x_, EtO_x_, PrO_x_, and BuO_x_)
Applied peak voltage (kV)	9.5
Substrate	Polypropylene (PP)
Plasma gas	Ar
Plasma gas flow (SLM)	3
Vapor gas flow for monomer (SLM)	7
Plasma treatment time (min)	5
Functional groups of polymer film	Amide, imine
Cell type	Human foreskin fibroblasts (HFFs)
Number of incubated cells (cells/1000 μL)	10.000

**Table 15:** Summary of experimental synthesis and antibacterial results corresponding to Figure 27 [49].

Precursor	3–methylphenol (M–cresol, C_7_H_8_O)
Synthesis method	DBD–plasma
Plasma voltage (V_p–p_, kV)	30
Frequency (kHz)	40
Substrate	Silicon wafer
Plasma gas	Open air
Plasma synthesis time (s)	30
Functional groups of polymer film	Benzene ring with a hydroxyl functional group
Cell type	*E. coli*
Number of incubated cells (μL)	10

**Table 16:** Experimental conditions of AP PE–CVD across three steps for PEOA and PEOB deposition [50].

Deposition Step	Sample Notification	C2H4 (sccm)	He–TEGDME (SLM)	He (SLM)	Voltage (kV_p–p_)	Frequency (kHz)	Deposition (s)
Step 1		8		8	8.5	16, 26	60
Step 2		8	3.15	4.85	8.5	16, 26	10
Step 3	PEOA PEOB		3.15 3.15	4.85 6.85	6.5, 8.5 6.5, 8.5	16, 26 26	300 300

**Table 17:** Summary of experimental synthesis and antibacterial results corresponding to Figure 28 and Figure 29 [50].

Precursor	Vinyl acetate (VAc), Diethylene glycol dimethyl ether (DEGDME)
Synthesis method	LP and AP PE–CVD
Substrate	Ti, polycarbonate (PC)
Plasma gas	He
Functional groups of polymer film	C–OH, Ester/carboxyl groups (–COOR (H))
Cell type	Human dermal fibroblasts (HDFs), osteoblasts
Number of incubated cells (cells/mL)	1 × 10^5^

**Table 18:** Experimental conditions for PEO deposition [51].

Sample Notification		DEGDME (sccm)	Ar (sccm)	O_2_ (mTorr)	Power (W)	Deposition (min)
PEO1		0.4	5		5	60
PEO2		0.4	5		10	60
Ox			100	20	200	15
Ox+PEO1	Step 1		100	20	200	15
	Step 2	0.4	5		5	60
Ox+PEO2	Step 1		100	20	200	15
	Step 2	0.4	5		10	60

**Table 19:** Summary of experimental synthesis and bacterial results for Figure 30 and Figure 31 [51].

Polymer	PEO
Synthesis method	LPP–RF plasma
Plasma power (W)	5 (PEO 1), 10 (PEO 2)
Plasma gas	Ar, O_2_
Substrate	PCL scaffolds
Functional groups of polymer	Oxygen-containing functionalities
Cell type	Saos–2 osteoblast cells
Number of incubated cells (cells/well)	5 × 10^4^

**Table 20:** Summary of experimental synthesis and antibacterial results corresponding to Figure 32 and Figure 33 [52].

Polymer	Mg contained 3D–PCL scaffolds
Synthesis method	LPP–RF sputtering
Plasma power (W)	50
Plasma gas	Ar, H_2_O, H_2_, Ar/H_2_O, and Ar/H_2_
Total gas flow (sccm)	20
Deposition time (min)	60
Cell type	Saos–2 osteoblast cells
Number of incubated cells (cells/well)	5 × 10^4^

**Table 21:** Summary of experimental synthesis and antibacterial results corresponding to Figure 34 and Figure 35 [53].

Precursor	EGDMA, DOMAm monomers
Polymer	Poly(EGDMA–co–DOMAm) copolymer
Synthesis method	APP–DBD plasma
Discharge waveform	Sinusoidal pulse
Frequency (kHz)	10
Plasma power (W/cm^2^)	1.6
Substrate	Zirconia
Plasma gas	Ar
Deposition time (s)	8
Plasma gas flow (SLM)	20
Functional groups of PP film	Amide (–CONH), Ester group (–COO–)
Cell type	Human osteoblast–like MG–63 cells
Number of incubated cells (cells/well)	2 × 10^4^

**Table 22:** Identification of plasma–deposited samples [54].

Identification	Sample Conditions
PCL–ref	Electrospinning PCL NFs
PCL–COOH	Plasma polymer coating on PCL–ref
PCL–P1	PRP treatment on PCL–ref
PCL–COOH–P2	PRP treatment on PCL–COOH
PCL–COOH–P3	DCC treatment on PCL–COOH

**Table 23:** Summary of experimental plasma synthesis and antibacterial results corresponding to Figure 36 and Figure 37 [54].

Precursor	Acetylene (C_2_H_2_)
Synthesis method	LPP–RF plasma
Plasma power (W)	500
Substrate	Polycaprolactone nanofibers (PCL NFs)
Plasma gas	Ar/CO_2_/C_2_H_4_
Plasma gas flow (sccm)	50/16.2/6.2
Deposition time (min)	15
Functional groups of PP film	Amides (N–C=O), –COOH, C=O
Cell type	Bone marrow (BM) mesenchymal stromal cells (MSCs)
Number of incubated cells (cells/well)	5 × 10^3^

**Table 24:** Summary of plasma synthesis and biomedical experiments reported in recent studies without copyright permission approval [55,56,57,58].

No	Object	Plasma Source	Application	Year	Author Reference
1	Furfuryl methacrylate (FMA)	LPP—RF plasma (13.56 MHz)	Cell Adhesion	2016	Shirazi et al. [57]
2	APTES	APP–DBD Ar plasma	Cell Adhesion	2020	Chen et al. [58]
3	Polydopamine (PDA)	APP–PECVD	Cell Adhesion	2018	Czuba et al. [59]
4	Maleic anhydride (MA) and cetylene	APP–DBD RF plasma (13.56 MHz)	Cell Adhesion	2017	Manakhov et al. [60]

**Table 25:** Summary of plasma-based synthesis methods for polymer films for biomedical applications.

No	Object	Plasma Source	Application	Year	Author Reference
1	AgNP/CS nanocomposite	APP solution plasma	Antibacterial	2020	Sun et al. [34]
2	Au, AgNP/PVA nanocomposite	APP solution plasma	Antibacterial	2018	Nolan et al. [35]
3	3–(Aminopropyl)triethoxysilane (APTES)	APPJ	Cell adhesion	2022	Sainz–García et al. [12]
4	PP–lim	APPJ	Antibacterial	2023	Masood et al. [36]
5	ppAAc, ppAAm, ppAAOH	APP plasma RF (13.56 MHz)	Cell adhesion	2016	Smith et al. [37]
6	Polyoxazoline (pPO_x_)	APP–DBD plasma, (6 kHz, 55 W)	Antibacterial	2020	Mazánková et al. [21]
7	Polyoxazoline (pPO_x_)	APP–DBD plasma, (6 kHz, 55 W)	Antibacterial	2019	St’ahel et al. [38]
8	Au NP/PDA nanocomposite	APP solution plasma	Medical	2021	Nguyen et al. [39]
9	Au NP/PDA nanocomposite	APP solution plasma	Medical	2020	Nguyen et al. [40]
10	PpHEPTYL	APPJ (8 kV_p–p_, 10 kHz)	Cell adhesion	2019	Doherty et al. [41]
11	Acetic acid	LPP plasma RF (13.56 MHz)	Cell adhesion	2019	Liao et al. [42]
12	polyHMDSO, polyallyamine	PECVD RF (13.56 MHz)	Cell adhesion	2019	Teske et al. [43]
13	Cyclopropylamine (CPA)	LPP–RF plasma (13.56 MHz)	Cell adhesion	2016	Štrbková et al. [44]
14	Polyoxazoline (pPO_x_) thin films	LPP–RF plasma (13.56 MHz)	Medical	2021	Gheorghiu et al. [45]
15	L–PPA:N film	LPP–PECVD (13.56 MHz)	Cell adhesion	2017	Ghafouri et al. [46]
16	N_2_–rich acetylene polymer film	LPP–RF plasma (13.56 MHz)	Cell adhesion	2021	Sharifahmadian et al. [47]
17	pPMeO_x_, pPEtO_x_, pPPrO_x_, and pPBuO_x_	LPP–DBD Ar plasma (50 kHz)	Cell adhesion	2019	Van Guyse et al. [48]
18	M–cresol	APP–DBD plasma	Antibacterial	2022	Hartl et al. [49]
19	Polyethylene oxide (PEO)	LPP, APP–PECVD (13.56 MHz)	Cell adhesion	2020	Sardella et al. [50]
20	Polyethylene oxide (PEO) scaffolds	LPP–DBD RF plasma (13.56 MHz)	Cell adhesion	2017	Sardella et al. [51]
21	Mg coated poly-ε caprolactone (PCL)	LPP–DBD RF plasma (13.56 MHz)	Cell adhesion	2020	Armenise et al. [52]
22	Polydopamine (PDA)/acrylate copolymer	APP–PECVD	Cell adhesion	2021	Hod’asov’a et al. [53]
23	Polycaprolactone (PCL) NFs	LPP–RF plasma (13.56 MHz)	Cell Adhesion	2017	Solovieva et al. [54]
24	Furfuryl methacrylate (FMA)	LPP–RF plasma (13.56 MHz)	Cell Adhesion	2016	Shirazi et al. [55]
25	3–(Aminopropyl)triethoxysilane (APTES)	APP–DBD Ar plasma	Cell Adhesion	2020	Chen et al. [56]
26	Polydopamine (PDA)	APP–PECVD	Cell Adhesion	2018	Czuba et al. [57]
27	Maleic anhydride (MA) and acetylene	APP–DBD RF plasma (13.56 MHz)	Cell Adhesion	2017	Manakhov et al. [58]

**Table 26:** Summary of experimental surface treatment conditions and antibacterial results corresponding to Figure 38 and Figure 39 [59].

Polymer	POx
Surface treatment method	APP–DCSBD
Applied voltage (kV)	20
Frequency (kHz)	15
Plasma gas	Air, Ar
Plasma gas flow rate (L/min)	1
Functional groups of POx film	Amines, ester, carbonyl and amide
Roughness (nm)	0.7 (PMEOx–air), 25.8 (after PMEOx deposition)
WCA (°)	59.5 (PMEOx–air), 61.4 (PMEOx–Ar)
Cell type	Mammalian (mice fibroblasts) cells

**Table 27:** Summary of experimental surface treatment conditions and antibacterial results corresponding to Figure 40 and Figure 41 [60].

Polymer	PLA
Surface treatment method	In situ APP (DBD type) with 3D printing technique
Plasma voltage (kV)	15
Plasma gas	Ar
Plasma gas flow rate (L/min)	1.5
Functional groups of 3D-printed PLA	C=O, C–O bonds
Roughness (R_q_) of PLA (nm)	1.5 (untreated), 70 (Ar–treated)
WCA (°)	92.5 (untreated), 42.2 (Ar–treated)
Cell type	Human adipose-derived stem cells (hADSCs)

**Table 28:** Summary of experimental surface treatment conditions and antibacterial results corresponding to Figure 42 and Figure 43 [61].

Polymer	Cyclic olefin copolymer (COC)
Surface treatment method	LPP, HPP inductive plasma
Plasma power (W)	7.2 (LPP), 29.6 (HPP)
Plasma gas	O_2_
Plasma treatment time (s, min)	10 s (LPP), 10 s, 10 min (HPP)
Roughness (R_q_) of COC (nm)	2.5 (O_2_–treated)
WCA (°)	110 (untreated), 20 (O_2_-treated)
Cell type	Human breast cancer cells
Number of incubated cells	10 mL containing 5 × 10^5^ cells

**Table 29:** Summary of experimental plasma synthesis and antibacterial results corresponding to Figure 44 and Figure 45 [62].

Precursor	CS scaffolds
Surface treatment method	DBD, soft–jet plasma (APPJ)
Applied peak voltage (V_p_, kV)	2 (DBD), 0.5 (soft–jet)
Plasma gas	N_2_
Plasma gas flow (lpm)	1.5
Plasma treatment time (min)	5
Functional groups of CS scaffolds	Amino and hydroxyl groups
Cell type	Bone marrow (BM)–derived stem cells (BMSCs)
Number of incubated cells (cells)	1 × 10^5^

**Table 30:** Summary of experimental plasma surface treatment conditions and antibacterial results corresponding to Figure 46 and Figure 47 [63].

Material	Poly(vinyl alcohol) (PVA)
Plasma method	DBD–APPJ
Discharge voltage (kV)	9 V_p–p_
Frequency (kHz)	30
Plasma gas	He
Plasma gas flow (SLM)	0.5
Plasma treatment time (min)	20
Functional groups of PVA film	RNS, H_2_O_2_
Cell type	*E. coli*, *P. Aeruginosa*, and *S. aureus*
Number of incubated cells (cells/mL)	1 × 10^6^
Zone of inhibition (ZOI) (mm)	>10 (He plasma treatment at 20 min)

**Table 31:** Summary of experimental plasma surface treatment conditions and antibacterial results corresponding to Figure 48 [64].

Material	PCL, P(3HB)
Plasma gas	O_2_, CO_2_
Functional groups of PCL and P(3HB)	Amino, oxygen group
WCA (°)	PCL: 71 (untreated), 50 (CO_2_), 51 (O_2_) P(3HB): 69 (untreated), 44 (CO_2_), 44 (O_2_)
Cell type	Mouse fibroblast cells (L929)
Cell viability (%)	PCL:98 (CO_2_), 107 (O_2_) P(3HB): 114 (CO_2_), 76 (O_2_)

**Table 32:** Summary of experimental plasma surface treatment conditions and antibacterial results corresponding to Figure 49 and Figure 50 [62].

Polymer material	PEO–PEOT/PBT thin film
Plasma treatment method	MP–DBD
Plasma power (W)	3 (Ar, Dry air), 6 (He, N_2_)
Frequency (kHz)	50
Plasma gas	Ar, He, N_2_, Dry air
Plasma gas flow (SLM)	3
Plasma treatment time (min)	3
Functional groups	C–O, C–N
WCA (°)	59 (Untreated) 45 (Ar), 31 (N_2_)
Cell type	Human foreskin fibroblast (HFF)
Number of incubated cells (cells/mL)	40,000

**Table 33:** Summary of experimental plasma surface treatment conditions and antibacterial results corresponding to Figure 51 [66].

Polymer material	dECM thin film
Plasma method	Microwave–LPP
Plasma power (kW)	100
Plasma gas	N_2_, H_2_
Plasma gas pressure (mTorr)	300
Plasma treatment time (s)	60
Functional groups	Amine and amide groups (C–O/C–N, –N–C=O)
WCA (°)	92.4 (Untreated), 86.1 (plasma–treated)
Cell type	Human dermal fibroblasts (HDFs)
Number of incubated cells (cells/cm^2^)	5 × 10^4^

**Table 34:** Identification of APPJ–treated and UV–grafted hydrogels on 3D–printed PLA [67].

Sample Label	APPJ Treatment	UV–Graftrf Hydrogels	
	Time (s)	HEMA (mL)	PEGMA (mL)
Untreated			
Treatment A	60 s		
Treatment B	90 s		
Treatment A—H1	60 s	15	15
Treatment A—H2	60 s	10	20
Treatment A—H3	60 s	20	10
Treatment B—H1	90 s	15	15
Treatment B—H2	90 s	10	20
Treatment B—H3	90 s	20	10

**Table 35:** Summary of experimental plasma treatment conditions and bacterial evaluation results corresponding to Figure 52 and Figure 53 [67].

Polymer	Poly(lactic acid) (PLA), HEMA, PEGMA
Plasma treatment method	APPJ
Plasma power (W)	600
Plasma gas	Ar
Plasma gas flow (SLM)	20
Plasma treatment time (s)	60, 90
Functional groups of PP film	C–O–C, carbonyl peaks (C=O)
Cell type	Osteoblast MG63 cells

**Table 36:** Summary of experimental plasma surface treatment conditions and antibacterial results corresponding to Figure 54 and Figure 55 [68].

Polymer	Low–density polyethylene (LDPE) film
Plasma treatment method	APPJ plasma
Voltage waveform	AC sinusoidal signal
Applied voltage (kV_p–p_)	9
Frequency (kHz)	36
Plasma gas	He
Plasma gas flow (L/min)	8.1
Plasma treatment time (s)	10
Functional groups of PP film	C–OH/R, C–O, COOH, and C–O–O–C
Cell type	DNA
Number of incubated cells (μL)	160

**Table 37:** Summary of plasma treatment conditions and antibacterial evaluation results corresponding to Figure 56 and Figure 57 [69].

Polymer	LDPE, poly–ε–caprolactone (PCL)
Plasma treatment method	APPJ plasma
Voltage waveform	AC sinusoidal signal
Average plasma power (W)	3.2
Frequency (kHz)	31.1
Plasma gas	He
Plasma gas flow (L/min)	1.9
Plasma treatment time (s)	5
Functional groups of PP film	C=O, C=C
Cell type	GM3348 human dermal fibroblast cells
Number of incubated cells (cells/cm^2^)	5000

**Table 38:** Summary of plasma surface treatments and biomedical experiments from recent studies without copyright permission approval [70,71].

No	Object	Plasma Source	Application	Year	Author Reference
1	Polyvinyl alcohol/chitosan (PVA/Cs) films	APP–DBD Ar plasma	Antibacterial	2020	Paneru et al. [70]
2	Polystyrene (PS) films	DBD Air plasma (APPJ)	Cell adhesion	2018	Bitara et al. [71]

**Table 39:** Summary of plasma surface treatments of polymer films for biomedical applications.

No	Object	Plasma Source	Application	Year	Author Reference
1	PTFE, POx coating layer	APP plasma	Cell adhesion	2020	Šrámková et al. [59]
2	PLA scaffolds	In situ APP plasma	Cell adhesion	2023	Zarei et al. [60]
3	Cyclic olefin copolymer (COC)	LPP plasma	Cell adhesion	2022	Al-Azzam et al. [61]
4	CS scaffolds	DBD, soft jet plasma (APPJ)	Cell adhesion	2022	Han et al. [62]
5	PVA hydrogel film	APPJ (He, 9 kV_p–p_, 30 kHz)	Antibacterial	2024	Sabrin et al. [63]
6	PCL, P(3HB) film	CO_2_, O_2_ plasma	Antibacterial	2017	Teske et al. [64]
7	PEOT/PBT copolymer film	DBD plasma (50 kHz)	Cell adhesion	2018	Cools et al. [65]
8	dECMfs	LPP microwave plasma (100 kW)	Antibacterial	2024	Lombardo et al. [66]
9	3D-printed poly(lactic acid) (PLA)	APPJ	Cell adhesion	2023	Liao et al. [67]
10	Low-density polyethylene (LDPE)	APPJ	Cell adhesion	2024	Lotz et al. [68]
11	Low-density polyethylene (LDPE) Poly–ε caprolactone (PCL)	APPJ	Cell adhesion	2020	Alavi et al. [69]
12	Polyvinyl alcohol/chitosan (PVA/Cs) films	APP–DBD Ar plasma	Antibacterial	2020	Paneru et al. [70]
13	Polystyrene (PS) films	DBD Air plasma (APPJ)	Cell adhesion	2018	Bitara et al. [71]

**Table 40:** Summary of experimental plasma surface treatment conditions and antibacterial evaluation results corresponding to Figure 58 and Figure 59 [72].

Material	TIO2 NPs film
Plasma method	APP
Applied voltage (kV)	4.5
Frequency (kHz)	9
Plasma gas	O_2_, Air, He, N_2_
Plasma gas flow (SLM)	2
Plasma treatment time (min)	0, 1, 2, 3, 4, and 5
Cell type	*E. coli*
Number of incubated cells (CFU/mL)	10^6^

**Table 41:** Summary of experimental plasma synthesis and antibacterial evaluation results corresponding to Figure 60 and Figure 61 [73].

Precursor	Zn(NO_3_)_2_, ZnCl_2_, ZnSO_4_, and Na_2_S
Synthesis method	APPJ
Plasma synthesis time (h)	1
Cell type	*E. coli*, *S. aureus*
Number of incubated cells (CFU/mL)	10^6^

**Table 42:** Summary of experimental plasma synthesis conditions and antibacterial evaluation results corresponding to Figure 62 and Figure 63 [74].

Precursor	ZnCl_2_, NaOH
Synthesis method	AP soft jet (APPJ)
Applied voltage (kV)	0.5
Current (A)	0.1
Plasma gas	Air
Plasma gas flow (L/min)	2
Plasma synthesis time (hr)	1
Cell type	*E. coli*, *S. iniae*, *S. parauberis*, and *E. tarda*
Number of incubated cells (CFU/mL)	10^6^
Length of ZOI (mm)	3.4 (ZnO RO at *S. iniae*)

**Table 43:** Summary of experimental plasma synthesis procedures and antibacterial evaluation results corresponding to Figure 64 and Figure 65 [75].

Precursor	Chloroauric acid trihydrate (H[AuCl_4_]·3H_2_O)
Plasma synthesis	AP soft jet plasma (APPJ)
Applied voltage (kV)	2.2
Frequency (kHz)	42
Plasma gas	Air
Plasma gas flow (L/min)	1
Plasma treatment time (s)	200
Functional groups	Free radicals (RONS, H_2_O_2_)
Cell type	Brain cancer cells (U373)
Number of incubated cells (cells/mL)	5 × 10^4^

**Table 44:** Summary of plasma-based synthesis and surface treatment techniques for metal NPs for biomedical applications.

No	Object	Plasma Source	Plasma Process	Application	Year	Author Reference
1	TiO_2_ NPs	APP solution plasma	Plasma surface treatments	Antibacterial	2016	Zhou et al. [72]
2	ZnS NPs	Soft jet plasma (APPJ)	Plasma synthesis	Antibacterial	2020	Ananth et al. [73]
3	ZnO NPs	Soft jet plasma (APPJ)	Plasma synthesis	Antibacterial	2017	Ananth et al. [74]
4	AuQDs	APP soft jet plasma (APPJ)	Plasma synthesis	Medical	2020	Kaushik et al. [75]

**Table 45:** Summary of recent review articles related to CAP–based APPJ technique.

No	Object	Plasma Process	Year	Author Reference
1	Synthesis of polymer film and NPs	CAP, APPJ	2021	Jang et al. [76]
2	Plasma synthesis and surface treatment of polymer film and NPs for nanogenerators and sensors	CAP, APPJ	2024	Jung et al. [77]
3	Plasma techniques in biomedical field	CAP, APPJ	2024	Karthik et al. [78]
4	Plasma treatment in agriculture field	CAP, APPJ	2022	Waskow et al. [79]
5	Plasma techniques for NP synthesis	CAP	2022	Radetić et al. [80]
6	Plasma surface technology of polymer in industrial field	CAP	2022	Förster et al. [81]
7	Plasma polymerization of biogenic precursors	CAP, APPJ	2023	Loesch–Zhang et al. [82]
8	Plasma surface treatment of polymer	APPJ	2024	Bertin et al. [83]
9	Plasma synthesis of polymer in biomedical field	APPJ	2024	Rahman Khan et al. [84]
10	Plasma techniques in medical field	CAP, APPJ	2024	O’Neill et al. [85]

## Data Availability

No new data were created or analyzed in this study.

## Abbreviations

ALP	Alkaline phosphatase
Al	Aluminum
APP	Atmospheric pressure plasma
AP	Atmospheric pressure
AgNPs/CS	Silver nanoparticles/chitosan
APM	AP microplasma
Ar	Argon
AP–PP–lim	AP plasma–polymerized D–limonene
APPJ	Atmospheric pressure plasma jet
AFM	Atomic force microscope
AAc	Acrylic acid
AAm	Allyl amine
AAOH	Allyl alcohol
AAP	Acetic acid plasma
AC	Alternating current
APM	Atmospheric pressure microplasma
APTES	3–aminopropyl)triethoxysilane
APTD	Atmospheric pressure Townsend–like discharge
APS	Ammonium persulfate
AuQDs	Gold quantum dots
BM	Bone marrow
BMSCs	Bone marrow–derived stem cells
CAP	Cold atmospheric pressure plasma
CCHM	Coherence–controlled holographic microscopy
CFU	Colony–forming units
CPA	Cyclopropylamine
Cu	Copper
COC	Cyclic olefin copolymer
CS	Chitosan
DI	Deionized water
DBD	Dielectric barrier discharge
DCSBD	Diffuse coplanar surface barrier discharge
DS	Downstream
DCC	Dicyclohexylcarbodiimide
dECM	Decellularized extracellular matrix
dECMf	Decellularized extracellular matrix film
*E. coli*	*Escherichia coli*
ES	Embryonic stem
EGDMA	Ethylene glycol dimethacrylate
FCC	Face-centered cubic
FE–SEM	Field emission scanning electron microscopy
FT–IR	Fourier–transform infrared
FMA	Furfuryl methacrylate
GND	Ground
GO	Graphene oxide
HADSCs	Human adipose–derived stem cells
HDFs	Human dermal fibroblasts
He	Helium
HFF	Human foreskin fibroblast
H[AuCl_4_]·3H_2_O	Gold(III) chloroauric acid trihydrate
HEMA	2–hydroxyethyl methacrylate
H_2_O_2_	Hydrogen peroxide
HMDSO	Hexamethyldisiloxane
HAp	Hydroxyapatite
HV	High voltage
HPP	High–power plasma
OH	Hydroxyl radical
IPP	Ion–assisted plasma polymerization
LDPE	Low density polyethylene
LP	Low pressure
LPP	Low–power plasma
lPM	Liters per minute
LECs	Lens epithelial cells
LA–APPiP	Liquid–assisted atmospheric pressure plasma induced polymerization
M–cresol	3–methylphenol
MgO	Magnesium oxide
MP	Medium pressure
MA	Maleic anhydride
MSCs	Mesenchymal stem cells
MSCs	Mesenchymal stromal cells
NHDF	Normal human dermal fibroblast
NPs	Nanoparticles
NTCP	Non–thermal cold plasma
Na_2_S	Sodium sulfide
N_2_	Nitrogen
NO	Nitric oxide
NO_2_	Nitrogen dioxide
NaOH	Sodium hydroxide
O_2_	Oxygen
OES	Optical emission spectroscopy
OD	1,7, octadiene
PBS	Phosphate buffered saline
PRP	Platelet-rich plasma
ppHEPTYL–HE–PS	Plasma polymerized–heptylamine
PC	Polycarbonate
PCL	Poly–ε caprolactone
P(3HB)	Poly(3–hydroxybutyrate)
PDA	Polydopamine
PET	Polyethylene terephthalate
PE–CVD	Plasma enhanced chemical vapor deposition
PI	Propidium iodide
PLA	Polylactic acid
PP	Plasma polymer
PEO	Polyethylene oxide
POx	Poly(2–oxazolines)
PMEOx	Poly(2–methyl–2–oxazoline)–stat–(2–(3–butenyl)–2–oxazoline)
PPOx	Polyoxazoline
PEGMA	Poly(ethylene glycol) methacrylate
PLGA	Poly(DL–lactide–co–glycolide)
PR	Photoresist
PS	Polystyrene
Pt	Platinum
PVA	Polyvinyl alcohol
PTFE	Polytetrafluoroethylene
RF	Radio frequency
RT	Room temperature
ROS	Reactive oxygen species
RNS	Reactive nitrogen species
RONS	Reactive oxygen and nitrogen species
R_a_	Average roughness
R_q_	Root mean square roughness
Ag	Silver
*S. aureus*	*Staphylococcus aureus*
SPR	Surface plasmon resonance
SEM	Scanning electron microscope
SLM	Standard liter per minute
ToF–SIMS	Time–of–flight secondary ion mass spectrometry
TEM	Transmission electron microscope
TCPS	Tissue culture PS
Ti	Titanium
UT–PS	Untreated PS
V_p_	Peak voltage
V_p–p_	Peak–to–peak voltage
WCA	Water contact angle
XPS	X–ray photoelectron spectroscopy
XRD	X–ray diffraction
ZnS NPs	Zinc sulfide NPs
ZnS	Zinc sulfide
Zn(NO_3_)_2_	Zinc nitrate
ZnCl_2_	Zinc chloride
ZnSO_4_	Zinc sulfate
ZOI	Zone of inhibition
1D	One–dimensional
2D	Two–dimensional
